# Activation volumes associated with excited-state electron transfer across amidinium-carboxylate bridge

**DOI:** 10.1039/d6sc00291a

**Published:** 2026-04-01

**Authors:** Daniel Langford, René Weiß, Marcel Krug, Maxence Urbani, Achim Zahl, Carolin Müller, Timothy Clark, Tomás Torres, Dirk M. Guldi

**Affiliations:** a FAU Profile Center Solar, Department of Chemistry and Pharmacy & Interdisciplinary Center for Molecular Materials (ICMM), Friedrich-Alexander-Universität Erlangen-Nürnberg Egerlandstr. 3 Erlangen 91058 Germany dirk.guldi@fau.de; b Department of Chemistry and Pharmacy, Computer-Chemistry Center, Friedrich-Alexander-Universität Erlangen-Nürnberg Nägelsbachstr. 25 Erlangen 91052 Germany; c Instituto Madrileño de Estudios Avanzados (IMDEA) – Nanociencia C/ Faraday 9 Madrid 28049 Spain; d Department of Chemistry and Pharmacy, Chair of Chemistry of Thin Film Materials, Friedrich-Alexander-Universität Erlangen-Nürnberg Cauerstr. 3 Erlangen 91058 Germany; e Departamento de Química Orgánica & Institute for Advanced Research in Chemical Sciences (IAdChem), Universidad Autonoma de Madrid Spain

## Abstract

Pressure was used to modulate interactions in an electron donor–acceptor system, composed of a zinc porphyrin (ZnP) and a fullerene (C_60_), held together by an amidinium-carboxylate salt-bridge (ZnP-H⋯C_60_). Two different trends evolved in steady-state absorption assays. Volume compression causes an absorbance intensification, and a solvatochromic-like red shift that stems from increased E-field density. Pressure-dependent femtosecond and nanosecond transient-absorption experiments were performed to investigate the activation volumes of the excited-state deactivation processes in ZnP-H⋯C_60_. Solvent relaxation of S_1_ is found to have a highly positive Δ*V*_k_2__^‡^. The pressure-induced rate attenuation for this process is assumed to be linked to the solvent's viscosity increase. Intersystem crossing to the porphyrin-centered T_1_ state is free of intrinsic and extrinsic reorganizations and, as such, the activation volume is close to zero. The same applies for the subsequent ground-state deactivation from T_1_ to S_0_. Charge-separation to afford (ZnP)˙^+^⋯H⋯(C_60_)˙^−^ is linked to a volume compression towards the activated state with Δ*V*_k_3__^‡^ = −5.7 ± 2.2 cm^3^ mol^−1^. The charge-recombination undergoes, within the experimental margins of error, an equal volume expansion with Δ*V*_k_4__^‡^ = +8.6 ± 0.7 cm^3^ mol^−1^. This effect is linked to the generation and/or neutralization of charges, best described by the Jung equation for electrostrictive volume changes in dipolar zwitterionic entities. Importantly, volumetric contributions from a possible PT towards the activated state were not observed.

## Abstract 2

Pressure was used to modulate interactions in an electron donor–acceptor system, composed of a zinc porphyrin (ZnP) and a fullerene (C_60_), held together by an amidinium-carboxylate salt-bridge (ZnP-H⋯C_60_).

## Graphical abstract

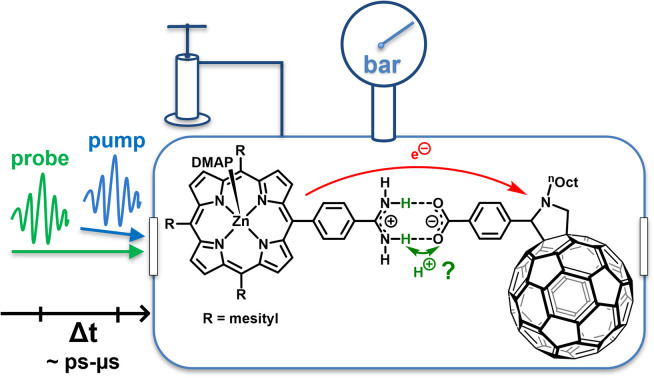

## Introduction

Nature has developed various photoenergy–driven reactions that build up the world around us.^1–5^ Attempts, for example, to mimic nature's light harvesting systems have received remarkable attention in recent research.^6–9^ one of the cornerstones in the pursuit of transforming our fossil fuel-based society towards renewables. Nature's sophistication in converting photoenergy to chemical fuels in photosynthesis has inspired research on porphyrins^10^ and porphyrinoid-based^11^ materials and their integration into electron donor–acceptor systems.

Their capability to donate electrons in their photoexcited states renders them a perfect building block for artificial electron donor–acceptor systems. The electron acceptors of choice are fullerenes:^12^ Various compositions and configurations have been investigated over the years.^12^ Bridges that connect electron donors and acceptors ranging from covalent bonds,^13,14^ and through supramolecular binding like π–π interaction,^15–19^ to neutral hydrogen bonds^20–23^ and hydrogen bonded salt bridges^24–27^ have been found to be important.

Electron transfer (ET) in biological systems like proteins is often coupled to a proton transfer (PT) in the hydrogen-bonded peptide environment. Leading examples are cytochrome-c oxidase in cell respiration,^28^ or photosystems I^29^ and II^30,31^ in photosynthesis. Investigations are, however, challenging because of the complexity of the protein's structure and dynamics. Therefore, mimics have been developed and investigated to gather insight into the fundamental processes. Among others,^21,32^ the amidinium-carboxylate salt bridge stands out. Its chemical versatility, combination with a plethora of electron donors and acceptors, and its structural similarity to arginine–aspartic acid dimers in proteins, are of utmost importance.^33^

**Fig. 1:**
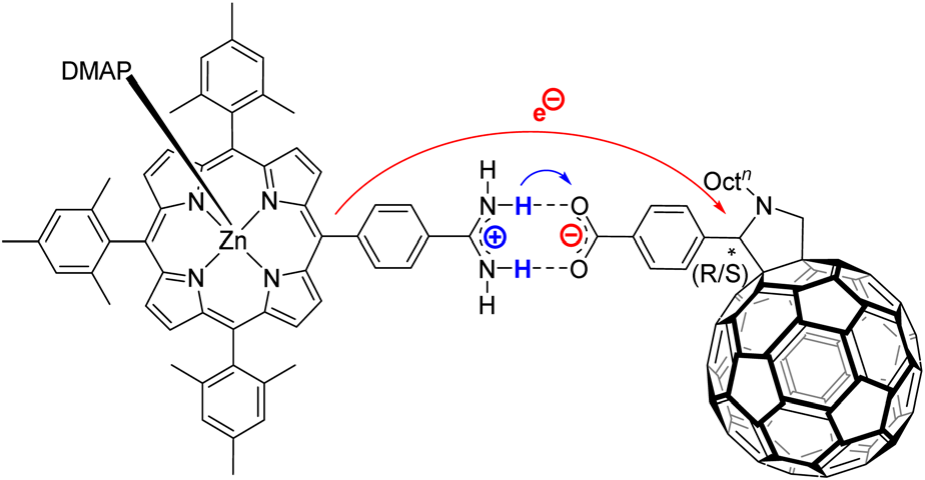
Molecular structure of (ZnP-H⋯C_60_): Zinc porphyrin (ZnP) connected to fullerene (C_60_) *via* an amidinium-carboxylate bridge. Coordination of ZnP was saturated by addition of 4-dimethylaminopyridine (DMAP) to avoid inter-porphyrin interactions.

The amidinium-carboxylate salt bridge was used as a blueprint to describe proton-coupled electron transfer (PCET) reactions,^34–38^ while they were studied with time-resolved spectroscopy experiments.^24–26,39–42^ Weak electronic couplings between the respective electron donors and acceptors, as seen in temperature-dependent transient-absorption experiments, suggests that the hydrogen-bonding interface is the bottleneck for electron transfer reactions.^43^ Additionally, the orientation of the salt-bridge was identified to influence the electron transfer (ET) rate significantly. If the salt-bridge is oriented as shown in Fig. 1, that is, amidinium on the electron donor and carboxylate on the electron acceptor, the electron transfer rate was attenuated compared to a hydrogen-bonded heterodimer with carboxylic acids on both the electron donor and electron acceptor.^24,39–42^ If the salt-bridge is oriented the other way round, that is, the carboxylate on the electron donor and the amidinium on the electron acceptor, a higher electron transfer rate was observed.^24,25^ The rationales for the attenuation are twofold. First, the adverse effect of the electric field across the bridge reduces the ET rate.^33^ Second, a coupling between the charge-separation and -recombination rate with the bridging protons' vibrational wavefunction has been reported.^43^ The notion of ET across the amidinium-carboxylate bridge being coupled to the proton configuration has been identified in temperature-dependent kinetic isotope effects (KIE). In particular, the thermal population of different vibrational modes alters the ET rate.^40,42^

Questions related to any ET coupling that goes beyond the bridging proton's vibrational configuration – implying that ET is coupled to a proton transfer towards the electron acceptor – could not be finally answered.^43^ In this notion, the photoexcitation of the electron donor, and/or the charge-separation forming to the one-electron oxidized porphyrin and one-electron reduced fullerene, are expected to come together with a significant change of the amidinium's and carboxylate's acidity/basicity properties.^33^ Most of these investigations were limited by the spectroscopic features associated with the electron transfer. Proton transfer across the amidinium-carboxylate bridge is often not spectroscopically observable. It is the lack of characteristic features in the observable region that hampers an unambiguous characterization. To date, clarification of the role of the bridging protons during the charge-separation and recombination through amidinium-carboxylate salt bridges remains a challenging task. The key factor for identifying the role of the bridging proton during the observable ET has been postulated to be embodied within reorganization- and solvation free energy changes, as ET or a PCET reaction are expected to yield differently charged products, which can be linked to differently charged intermediate states.^35,36,43^

According to Marcus–Hush theory, electron-transfer reaction rates k depend on both the free-energy difference Δ*G*^0^ and the reorganization energy *λ* of the reactants (eqn (1)). Reorganization in ET or PCET reaction is, meanwhile, best described by transition-state theory,^44^ which assumes that an electron donor A and an electron acceptor B are in equilibrium with an activated complex [AB]^‡^. Charge separation proceeds from [AB]^‡^ and generates C (eqn (2)). The reaction steps involved, H-bond dissociation, H-bond formation, and redistribution of electronic charges, are traceable by means of the corresponding activation volumes Δ*V*^‡^ (eqn (3)). Δ*V*^‡^ is governed by two contributions: first, intrinsic volume changes due to contraction, expansion, and reorientation of bonds within electron donating A and electron accepting B.^45^ Second, electrostrictive volume changes by reason of contraction, expansion, and reorientation of the solvation shell upon charge-separation.^46^ In general, Δ*V*^‡^ is a powerful tool to characterize ligand–substitution reactions in metal complexes.^47^ Using Δ*V*^‡^ to investigate reorganization processes has been a useful tool for characterizing ET reaction, but has found little attention for characterizing PCET reactions.^47–49^1
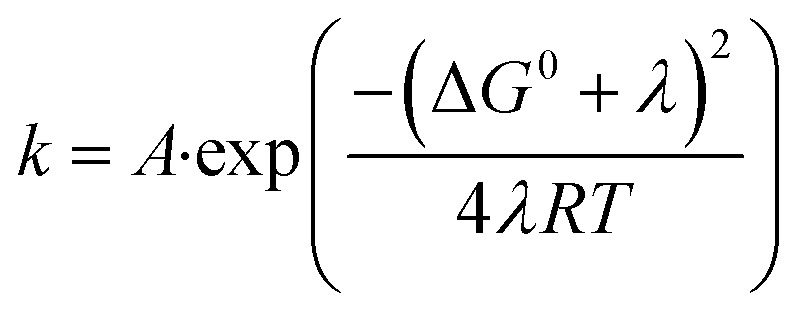

2 A + B ⇌ [AB]^‡^ → C

3 Δ*V*^‡^ = *V*^‡^_[AB]_ − (*V*_A_ + *V*_B_)

4
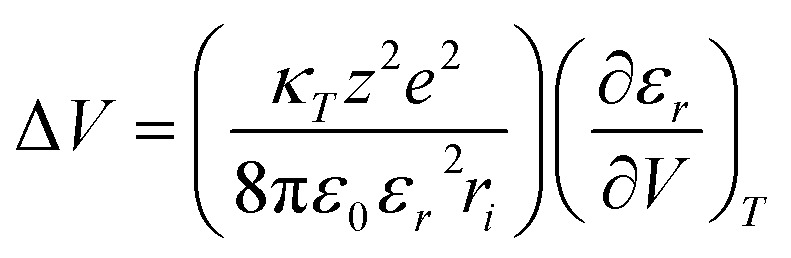
5
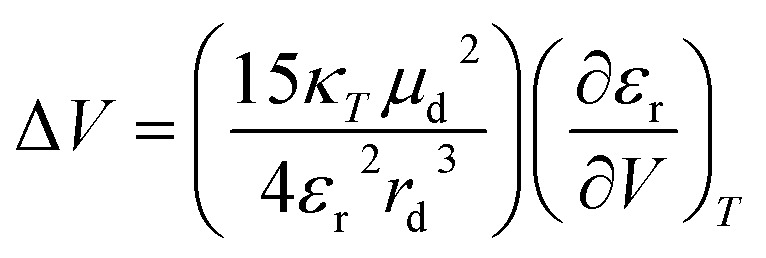
In previous work, we investigated the activation volumetric effect of an excited-state PCET reaction, which took place between a non-preorganized electron donor–acceptor couple that is freely dissolved in solution.^49^ In particular, we studied Haga-type [Ru(bpy)_2_pyimH]^2+^ as an excited-state electron and proton donor, together with *N*-methyl-4,4′bipyridinium as electron and proton acceptor in a buffered aqueous medium.^49^ Any volumetric effects were treated by the Drude–Nernst expression, which describes electrostrictive volume changes that are induced by charged ions in water (eqn (4)).^46,50^ In their formalism, volume changes depend on the charge of the ion sphere *z*_*e*_ and the ion radius *r*_*i*_, while compressibility of the medium *κ*_*T*_, relative permittivity *ε*_*r*_, and (*∂ε_r_*/∂*V*)_*T*_ remain constant.^46,50^ Volumetric characteristics together with the Drude–Nernst relation for electrostriction effect allowed insights into the PCET mechanism, that is, a stepwise PT-ET *versus* a stepwise ET-PT *versus* a concerted process prevails.^49^

Freely dissolved ions are, however, not formed upon charge-separation in covalently linked or preorganized electron donor–acceptor couples. Instead, the electron donor–acceptor couples become dipolar with zwitterionic character. Here, the Jung formulism is applicable, where volumetric changes induced by a formally spherical zwitterion depend on the radius *r*_*d*_ of the dipolar molecule and the dipole moment *µ*_*d*_, as described in eqn (5).^46,51^ Our present work aims to test the effects described by the Jung formulation by determining the activation volumes associated with an excited-state ET reaction across an amidinium-carboxylate bridged zinc porphyrin-fullerene (ZnP-H⋯C_60_) couple (Fig. 1). This work advances beyond our previous study, which focused on activation volume effects in independently dissolved electron donors and acceptors using Drude–Nernst theory, by investigating a preorganized electron donor–acceptor system through the Jung formalism.^49^ We establish a methodology to investigate pressure-induced effects in equilibria of supramolecular complexation based on pressure-dependent steady-state absorption and emission experiments. We gather a fully-fledged description of how hydrostatic pressure influences the electronic ground and excited states of (ZnP-H⋯C_60_) and how pressure influences their dynamics.

## Results and discussion

### Basic characterization

Previous work on electron donor–acceptor couples featuring porphyrins (ZnP) and fullerenes (C_60_) has shown that photoexciting ZnPs results either in an electron-transfer or an energy-transfer reaction to C_60_.^13,14,52,53^ The solvent polarity is decisive here. In low-polarity solvents like toluene, energy-transfer dominates over electron transfer. In high-polarity solvents, electron-transfer is observed exclusively.^13,14,52,53^ Importantly, solvents of high polarity like tetrahydrofuran, benzonitrile, or dimethylformamide interfere with intermolecular hydrogen-bonds.^10*a*,54^ As such, we took anisole for the current studies. Anisole is sufficiently polar to direct the photoreactivity to electron transfer and allows an effective formation of hydrogen-bonded amidinium-carboxylate bridge.

**Fig. 2:**
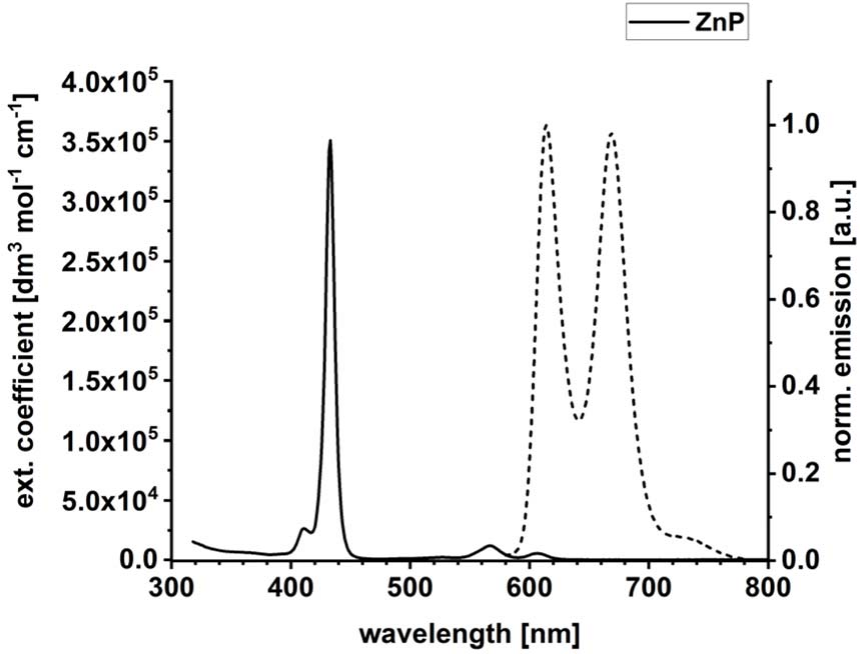
Absorption (solid) and emission (dashed) spectrum of ZnP in anisole. A 1000-fold excess of DMAP *vs.* ZnP was used in all optical spectroscopy experiments.

Steady-state absorption and fluorescence spectra of ZnP in anisole under ambient conditions are shown in Fig. 2, while the absorption spectrum of C_60_ is depicted in Fig. S21 in the SI. For all experiments with ZnP, a 1000-fold excess of 4-dimethylaminopyridine (DMAP) was necessary to suppress any π-stacking (Fig. S22 in the SI). Superimposed on the Soret-band absorption of ZnP at 432 nm (351 000 dm^3^ mol^−1^ cm^−1^) is a well-defined, blue-shifted shoulder with a maximum at 411 nm. Two Q-band absorption maxima evolve at 567 nm (11 900 dm^3^ mol^−1^ cm^−1^) and 607 nm (5400 dm^3^ mol^−1^ cm^−1^). The Soret-band absorption of ZnP at 432 nm is about 100-times stronger than the absorptions of C_60_ with 4000 dm^3^ mol^−1^ cm^−1^ (Fig. S21 in the SI). Hence, the Soret-band absorptions were used to photoexcite predominantly ZnP for all pressure-dependent experiments. Fluorescence maxima at 612 and 669 nm were recorded for ZnP upon photoexcitation. The ZnP fluorescence quantum yield was determined using the integrating sphere methodology resulting in values of 4.0% and 3.0% following Soret-band photoexcitation at 430 nm and Q-band photoexcitation at 567 nm, respectively. The fluorescence quantum yields from the two excitations are equal within experimental error.

**Fig. 3:**
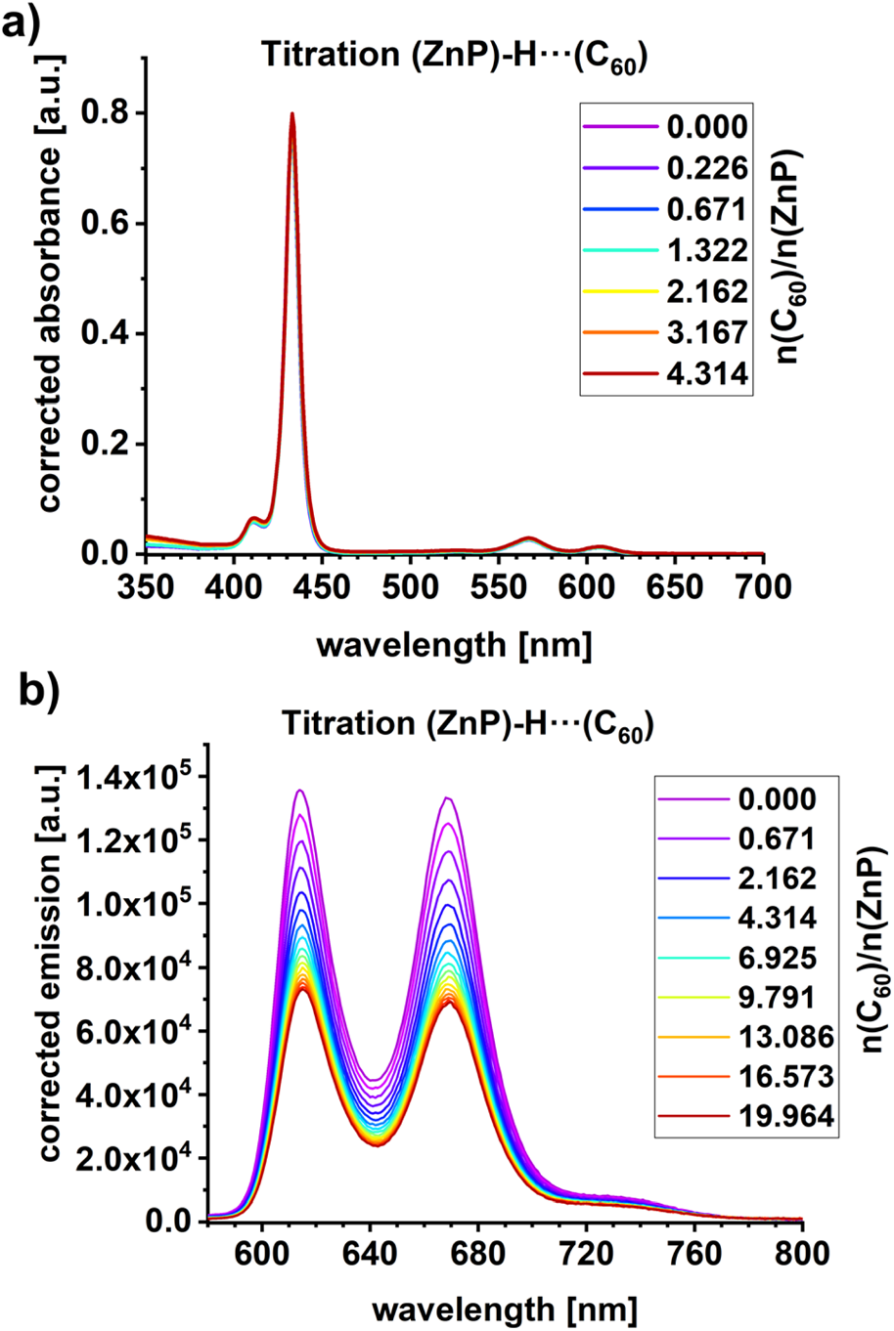
(a) Absorption spectra of ZnP with different amounts of C_60_. The corresponding C_60_ spectra were subtracted for visualization of ZnP-centered absorbance changes upon complexation. (b) Fluorescence spectra of ZnP photoexcited at 567 nm with different amounts of C_60_. Fluorescence spectra of C_60_ were subtracted for visualization of ZnP-centered fluorescence changes upon complexation. A 1000-fold excess of DMAP *vs.* ZnP was used in all optical spectroscopy experiments.

Titration experiments were conducted to investigate the complexation of C_60_ with ZnP (Fig. 3). Only subtle changes were observed in the absorption spectra upon gradual addition of C_60_, up to a 4-fold excess relative to ZnP. The mostly unaffected ZnP absorption is attributed to the fact that C_60_ lacks significant impact on the electronic properties of the Franck–Condon region. Notably, the Q- and B-band transitions are primarily associated with dipole-allowed π–π* excitations localized on ZnP. Time-dependent density functional theory (TDDFT) calculations support this interpretation (SI). Nearly identical π–π* transitions for the ZnP Q- and B-band transitions (S_1/2_: 2.16 eV; S_3/4_: 3.12 eV) and ZnP-H⋯C_60_ (S_1/2_: 2.17 eV; S_3/4_: 3.12 eV) are predicted (Fig. S25–S28 in the SI). In contrast, the fluorescence gave rise to a 50% reduction in intensity (Fig. 3b). We take the fluorescence quenching to indicate the presence of a new deactivation pathway in the ZnP-H⋯C_60_ complex that is not present in ZnP.

Using the steady-state fluorescence titrations (Fig. 3b), the binding constant was determined to be 1.9 × 10^5^ M^−1^ (Fig. S23 in the SI). This binding constant and the 50% reduction in fluorescence intensity are in line with previously reported data on comparable supramolecular amidinium-carboxylate salt bridged systems.^55–57^

Job's method of continuous variation was applied to confirm the ZnP-H⋯C_60_ stoichiometry. Parabolic maximum at a molar ratio of 0.5 (Fig. S24 in the SI) was identified, which confirms a 1 : 1 stoichiometry. Based on these results, we opted to use a 10-fold excess of C_60_ for all further experiments, to ensure that, according to the law of mass action, at least 90% of all photoexcited porphyrins are present as ZnP-H⋯C_60_, as depicted in Fig. 1.

To identify the most stable configuration in solution, we performed DFT calculations considering two primary conformations (see SI for computational details). First, ZnP-H⋯C_60_ with both bridging protons residing on the porphyrin, forming a positively charged amidinium adjacent to a negatively charged carboxylate (*η*^2^-binding motif between the porphyrin amidine group and C_60_). Second, ZnP⋯H-C_60_, in which the bridging hydrogen atoms are positioned on either side, forming a charge-neutral amidine-carboxylic acid bridge. DFT predicts ZnP-H⋯C_60_ to be 4.31 kcal mol^−1^ more stable than in ZnP⋯H-C_60_ (see Fig. S29, S30, and Tables S1–S3 in the SI).

### Pressure-dependent absorption and emission

**Fig. 4:**
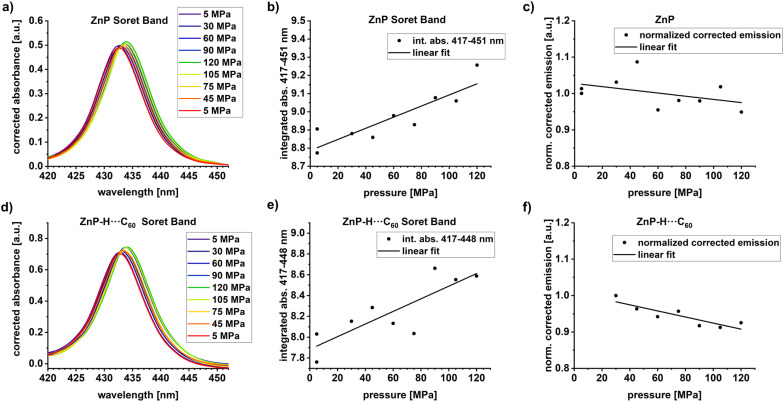
(a) Magnification of the ZnP Soret-band absorption in pressure dependent steady-state absorption measurements. (b) Plot of integrated Soret-band absorption area *vs.* pressure. (c) Integral of the corrected ZnP fluorescence from pressure dependent experiments using Soret-band photoexcitation at 430 nm, normalized to the first data point at 5 MPa. (d) Magnification of the ZnP-H⋯C_60_ Soret-band absorption from pressure dependent steady-state absorption measurements, and e) corresponding plot of integrated Soret-band absorption area *vs.* pressure. (f) Integral of the corrected fluorescence of ZnP-H⋯C_60_ recorded in the pressure dependent experiments using Soret-band photoexcitation at 430 nm, normalized to the first data point at 30 MPa. A 1000-fold excess of DMAP *vs.* ZnP was used in all optical spectroscopy experiments.

To evaluate the effect of pressure, we started our investigation with pressure dependent steady-state absorption assays.^58^ Our experimental setup used is described in the SI.^49,58^ We used 5 × 10^−6^ M ZnP in anisole and a 10-fold molar excess of C_60_. Each series of measurements was conducted in the pressurization and depressurization direction to check for the reversibility of the pressure effects. After each new pressure was set, the system was allowed to equilibrate for 15 minutes. All individual spectra were corrected by subtracting either the spectrum of the pure solvent in the ZnP experiments or that of C_60_ in the experiments with ZnP-H⋯C_60_. The full absorption spectrum under the given conditions is shown in Fig. S31 in the SI. Magnifications of the Soret-band absorptions of ZnP and ZnP-H⋯C_60_ are shown in Fig. 4a and d.

Pressure induced two different effects on ZnP-H⋯C_60_ and ZnP. On the one hand, absorption increases as a function of pressure, and, on the other hand, a bathochromic shift from 432 to 434 nm is seen. Neither of these effects is, however, linked to any perturbations of the underlying equilibrium. Instead, we believe that they are caused by a volumetric compression and a density dependent analogue of the solvatochromic effect.^59,60^ Any volume reduction associated with increasing pressure leads to higher dielectric constants and bathochromic shifts of the S_0_–S_2_ transitions of ZnP.^59,60^ The low intensity/signal-to-noise ratio of the Q-band absorptions under our experimental conditions impedes any meaningful evaluation of a pressure-induced perturbation of the S_0_–S_1_ transition.

When analyzing the pressure dependent steady-state fluorescence data it is, however, crucial to consider the absorption intensification. The objective is to subtract any effects due to volumetric compression. We quantified the absorption intensification by linear fits (*y* = *a*_*K*_ + *b*_*K*_*·x*) of the integrated Soret-band absorption. In doing so, we determined the compression factor *K* as described in eqn (6), with the highest applied pressures (*p*_max_ = 120 MPa) and lowest applied pressure (*p*_min_ = 5 MPa for ZnP, 30 MPa for ZnP-H⋯C_60_).6
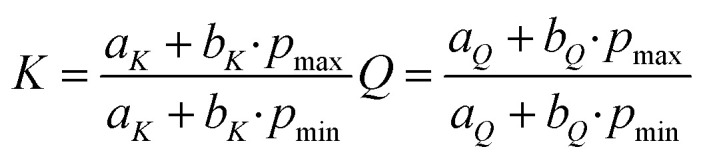


The compression factor *K* was used to calculate the fluorescence correction factor *E*_*i*_ for each individual measurement *i* as outlined in eqn (7).7
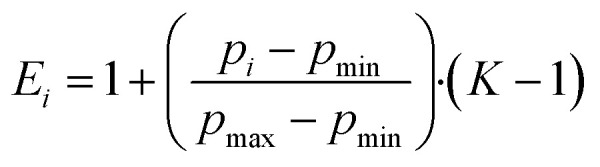


The fluorescence intensities *I*_0,*i*_, as obtained by integrating the measured fluorescence (Fig. S32 and S33 in the SI), were corrected by dividing them with the fluorescence correction factor *E*_*i*_ and gave the corrected fluorescence intensities *I*_*i*_.8
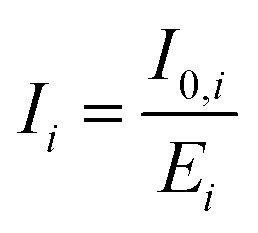


The pressure induced fluorescence quenching factor *Q* was then calculated from the linear fits (*y* = *a*_*Q*_ + *b*_*Q*_*·x*) of *I*_*i*_*versus* the applied pressure with the same procedure as used for the compression factor *K*, shown in eqn (6). All results for *K* and *Q* are listed in Table 1 and the corresponding plots are shown in Fig. 4.

**Table 1:** Pressure induced compression factor *K* and quenching factor *Q* from pressure dependent absorption and fluoresence experiments

	ZnP	ZnP-H⋯C_60_
*K*	1.04 ± 0.008	1.09 ± 0.02
*Q*	0.95 ± 0.04	0.90 ± 0.03

The compression factor *K* = 1.04 for ZnP represents a 4% increase of the integrated Soret-band absorption area upon increasing the pressure from 5 to 120 MPa. Correspondingly a *K* of 1.09 for ZnP-H⋯C_60_ represents a 9% increase. The fluorescence intensity of ZnP at 120 MPa corresponded to 95% of the intensity at 5 MPa, which relates to lower fluorescence quantum yields; 4.0% at ambient pressure *versus* 3.8% at 120 MPa. The emission observed for ZnP-H⋯C_60_ was lowered to 90%. The slightly stronger pressure-induced fluorescence quenching in ZnP-H⋯C_60_ compared with ZnP will be revisited in the discussions for the activation volumes.

Statistical noise impedes direct data interpretation. At this point, noise-correction was deemed necessary to evaluate any pressure-induced effects. We employed a multi-Gaussian fitting to model the emission spectra. The cumulated fit peaks of the multi-Gaussian fitting – Fig. S35–S41 – resemble the noise-subtracted emission spectra. A closer examination of the normalized cumulative high-energy fluorescence fits reveals a pressure induced bathochromic shift of the 611.4 nm maximum at 30 MPa to 612.2 nm at 120 MPa (Fig. S42). In other words, the emissive S_1_–S_0_ transition is also subject to a pressure-induced bathochromic shift, albeit less pronounced than the absorptive S_0_–S_2_ transition.^59,60^

### Transient absorption spectroscopy

**Fig. 5:**
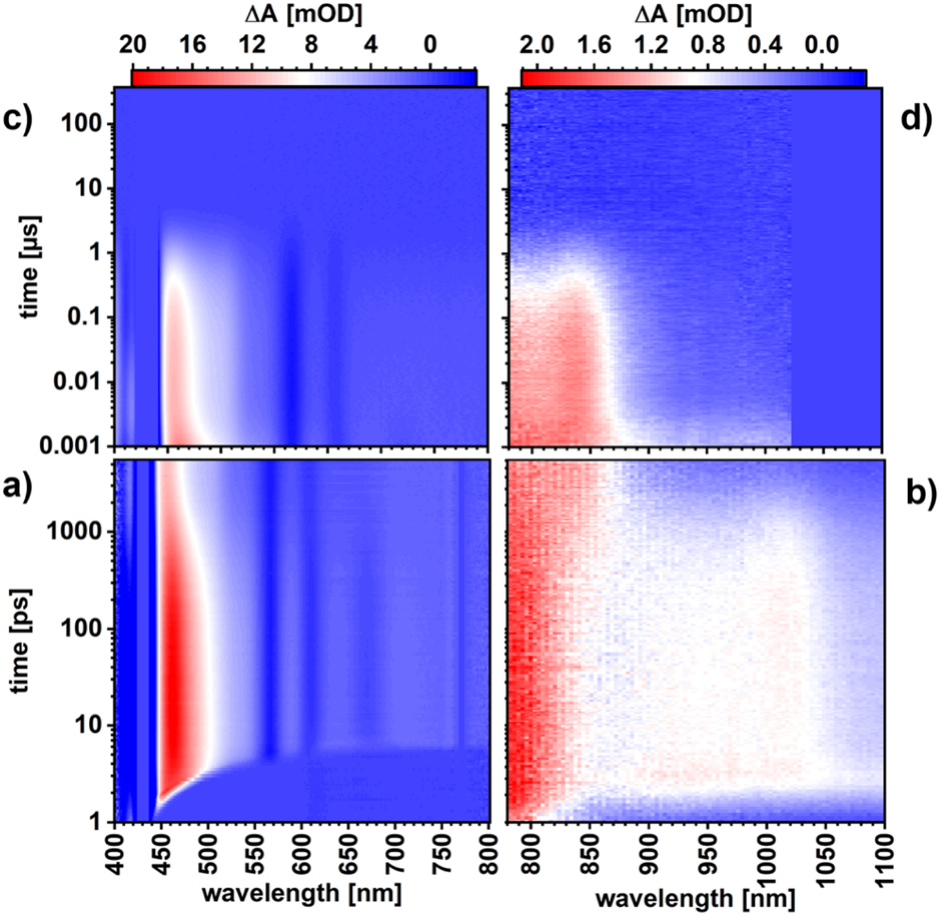
fs-TAS spectra in the visible (a) and NIR region (b) of ZnP-H⋯C_60_ at 5 MPa in anisole, after photoexcitation at 430 nm with 200 nJ pulse energy. ns-TAS spectra in the visible (c) and NIR (d) under identical condition. A 1000-fold excess of DMAP *vs.* ZnP was used in all optical spectroscopy experiments.

**Fig. 6:**
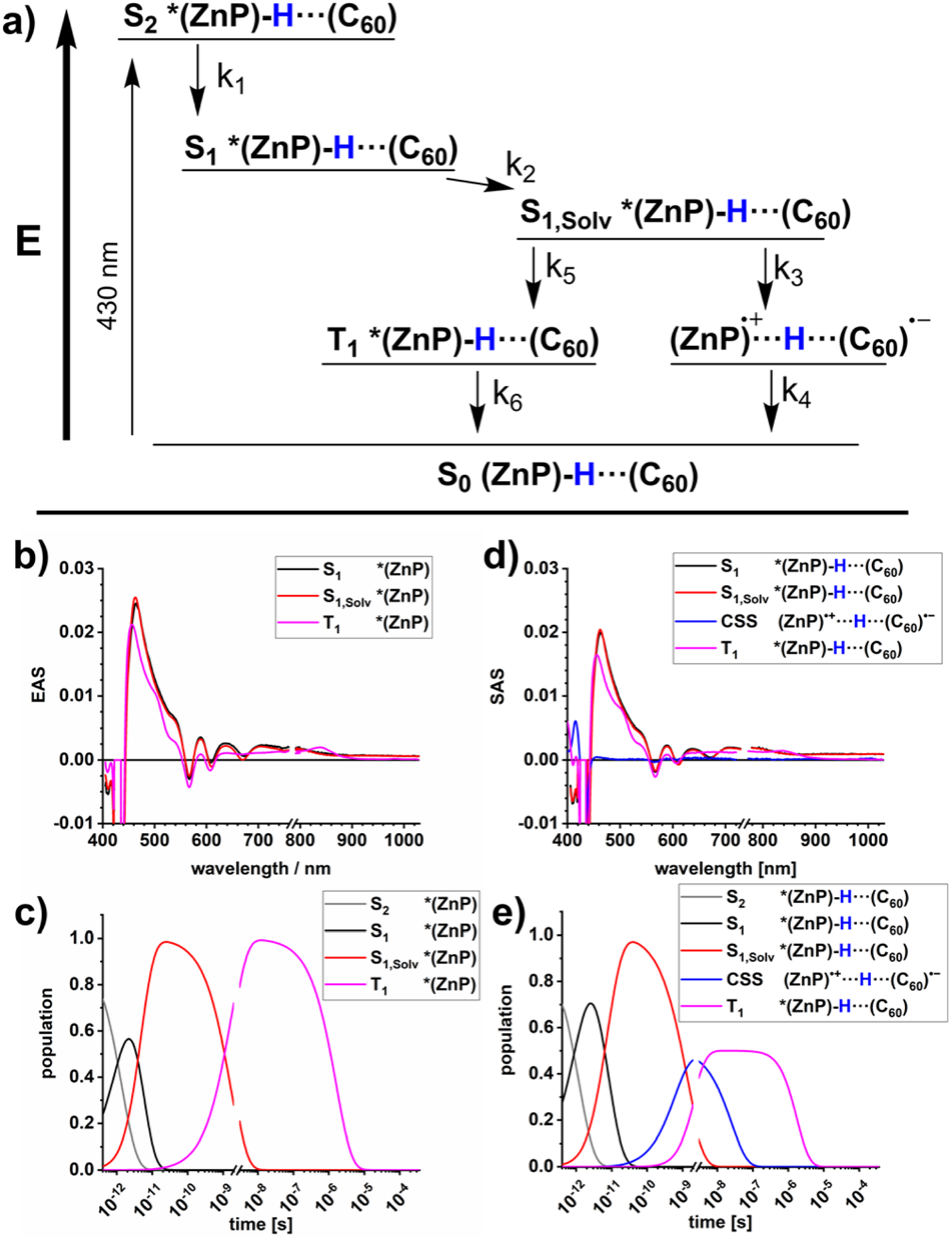
(a) Analysis model used for evaluating the transient-absorption spectra of ZnP and ZnP-H⋯C_60_. General note: Soret-band excitation results in the initial population of S_2_. Modelling the S_2_–S_1_ internal conversion was necessary for a good fit. However, the long optical dispersion through the pressure cell does not allow to assign a clear EAS or SAS for S_2_.^61^ (b) Evolution associated spectra (EAS) from a four exponential global analysis of ZnP in anisole at 5 MPa.^61^ (c) Time profiles depicting the time-resolved population of the corresponding EAS of ZnP from global analysis.^61,62^ (d) Species associated spectra (SAS) from branched six exponential target analysis of ZnP-H⋯C_60_ in anisole at 5 MPa.^61,62^ (e) Time profiles depicting the time-resolved population of the corresponding SAS of ZnP-H⋯C_60_, from target analysis. A 1000-fold excess of DMAP *vs.* ZnP was used in all optical spectroscopy experiments.^61,62^

The excited-state characteristics of ZnP and ZnP-H⋯C_60_ were probed in anisole *via* femtosecond- and nanosecond transient absorption spectroscopy (fs-TAS and ns-TAS) involving photoexcitation into the Soret-band absorption at 430 nm. The fs-TAS and ns-TAS spectra of ZnP-H⋯C_60_ and ZnP are shown in Fig. 5 and S43 (in the SI), respectively. The model used for global or target analysis is shown in Fig. 6a.^61^ The reference experiments with ZnP were evaluated with global analysis based on a sequential four-species model without transitions k_3_ and k_4_.^61^ These transitions were required to fit the raw data of the ZnP-H⋯C_60_ in a branched target analysis model with five species.^61^ Corresponding evolution associated species (EAS) from global analysis of ZnP and the species associated spectra (SAS) for ZnP-H⋯C_60_, together with the time-resolved population profiles are shown in Fig. 6.^61,62^

The short-lived second singlet excited state (S_2_) that is populated following Soret-band photoexcitation could not be resolved fully in our pressure dependent fs-TAS. The 3 ps optical dispersion caused by the aqueous medium in the pressure cell (Fig. 5) hampered its full deconvolution. Nevertheless, a fast decay corresponding to an S_2_–S_1_ internal conversion with a characteristic lifetime of 1.3 ps for ZnP and 1.2 ps for ZnP-H⋯C_60_ was required to describe the evolution of the singlet excited state (S_1_) (Fig. 6). Thus, the first resolved species following Soret-band photoexcitation corresponds to the ZnP-centered S_1_ state. The corresponding fingerprints are excited-state absorption (ESA) maxima at 464, 588, 639, and 707 nm, a shoulder at 535 nm next to ground state bleaching (GSB) and stimulated emission (SE) minima at 564, 614, and 668 nm (Fig. 6b). This state follows a relaxation process towards the solvated singlet excited state (S_1,Solv._). S_1,Solv._ is discernible by a slight intensification of the 464 nm ESA maximum, which goes together with a reduction of the 464 nm full-width at half maximum (FWHM) from 53 to 50 nm. In the absence of an electron-accepting C_60_, S_1,solv._ undergoes intersystem crossing (ISC) to afford the triplet excited state (T_1_). The T_1_ fingerprints are a shoulder at 502 nm in the visible region and a new ESA maximum at 838 nm, while the former SE minimum at 668 nm is diminished. T_1_ deactivates to the ground state by means of oxygen quenching, as the pressure apparatus did not allow these experiments to be performed under deoxygenated conditions. When C_60_ is present, an additional deactivation pathway from S_1_,_Solv._ is present (Fig. 6d and e). It competes with ISC and is identified as charge-separation that affords a charge-separated state (CSS): formation of the radical ion pair state (ZnP)˙^+^⋯H⋯(C_60_)˙^−^. CSS is followed through the characteristic ESA of (ZnP)˙^+^ at 415 nm,^57^ while formation of C_60_˙^–^ at 1050 nm is hampered by the optical absorption of water that was used as pressure medium in the pressure cell with the 6 cm optical path length.^14,49^

Both the lifetimes at 5 MPa and the activation volumes of the corresponding transitions are summarized in Table 2. The S_1_ lifetime is 3.8 ps, while for ZnP-H⋯C_60_ we observed a longer time of 7.3 ps. At this point, we are unable to explain the significantly longer lifetime of S_1_. S_1,Solv_ features reveal comparable lifetimes of 1.80 ns in ZnP and 1.67 ns in ZnP-H⋯C_60_. The same applies for the subsequently formed T_1_, which decays with 1.61 µs or 1.82 µs, respectively. The S_1,Solv_ state lifetime, which reflects the charge-separation to generate (ZnP)˙^+^⋯H⋯(C_60_)˙^−^, is much shorter with 634 ps. The lifetime of CSS is 25.1 ns, during which the singlet ground state (S_0_) is recovered by means of charge-recombination.

**Table 2:** Lifetimes at 5 MPa and activation volumes from pressure-dependent transient absorption experiments

	Lifetimes						Activation volumes [cm^3^ mol^−1^]					
System	S_2_	S_1_	S_1,Solv._	CSS	S_1,Solv._	T_1_	S_2_–S_1_	S_1_–S_1,Solv._	S_1,Solv._–CSS	CSS–S_0_	S_1,Solv._–T_1_	T_1_–S_0_
	*τ*_1_ [ps]	*τ*_2_ [ps]	*τ*_3_ [ps]	*τ*_4_ [ns]	*τ*_5_ [ns]	*τ*_6_ [µs]	Δ*V*_k_1__^‡^	Δ*V*_k_2__^‡^	Δ*V*_k_3__^‡^	Δ*V*_k_4__^‡^	Δ*V*_k_5__^‡^	Δ*V*_k_6__^‡^
ZnP	1.3	3.8	—	—	1.80	1.61	−3.1 ± 0.6	+26 ± 3	—	—	0.0 ± 0.3	−1.9 ± 1.0
ZnP-H⋯C_60_	1.2	7.3	634	25.1	1.67	1.82	−2.6 ± 0.6	+12 ± 3	−5.7 ± 2	+8.6 ± 0.7	−0.6 ± 1.4	+0.5 ± 0.4

### Activation volumes

**Fig. 7:**
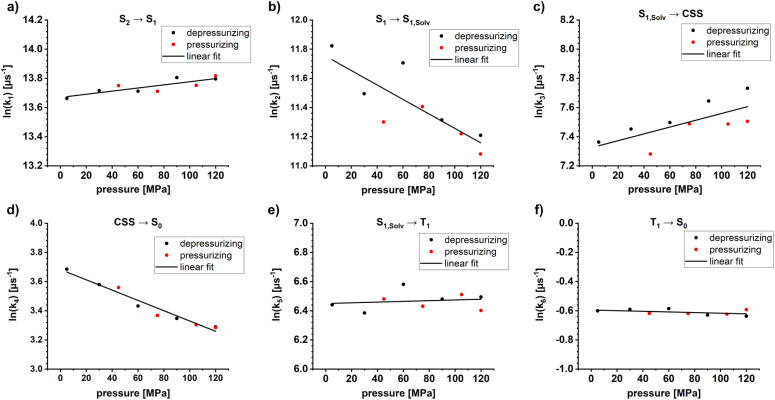
(a) Pressure effect on the reaction rate k_1_ of ZnP-H⋯C_60_ for S_2_–S_1_ conversion (b) Pressure effect on k_2_, associated with solvent relaxation from S_1_ to the solvated S_1,Solv_. (c) Pressure effect on k_3_, associated with the charge-separation from S_1,Solv._ towards the charge-separated state (CSS). (d) Pressure effect on k_4_, associated with the charge-recombination from CSS to S_0_. (e) Pressure effect on k_5_, associated with the intersystem crossing from S_1,Solv._ towards T_1_. (f) Pressure effect on k_6_, associated with the triplet excited state deactivation by means of oxygen quenching from T_1_ to S_0_. A 1000-fold excess of DMAP *vs.* ZnP was used in all optical spectroscopy experiments.

Insights into activation volumes Δ*V*^‡^ that correspond to these processes come from investigating the reaction kinetics k as a function of pressure *p*, as described by the Maxwell relation in eqn (9). We determined the activation volumes by performing fs-TAS and ns-TAS experiments under pressure. Δ*V*^‡^ values were calculated as described in the SI.^49^ The slopes of the ln(k)-*p* plots for ZnP-H⋯C_60_ are shown in Fig. 7, and those for ZnP in Fig. S48–S51. All relevant Δ*V*^‡^ values are listed in Table 2. The initial process in fs-TAS, the S_2_–S_1_ transition, is linked to a slightly negative Δ*V*_k_1__^‡^ (Fig. 7a and S48 in the SI). However, the 3 ps optical dispersion failed to capture S_2_ following photoexcitation into the Soret-band absorption fully – *vide supra*. Consequently, an unambiguous interpretation of the effects observed is not possible. A positive Δ*V*_k_2__^‡^ of more than +10 cm^3^ mol^−1^ (Fig. 7b and S49 in the SI) was determined for the subsequent solvent-relaxation process to yield S_1,Solv._. This corresponds to a significant rate deceleration as the pressure is increased. We link this effect to the pressure-induced increase in solvent viscosity.^59^ The spectral features of S_1_ and S_1,Solv._ differ, however, only marginally (Fig. 6b and e). This challenges the interpretation of any observable pressure-induced effects. ISC from S_1,Solv._ to T_1_ is an activation volume-free process, with Δ*V*_k_5__^‡^ values close to zero (Fig. 7e and S50 in the SI). It is porphyrin-centered and not subject to a change in charge density. As such, the intrinsic reorganization should be low and the influence on the solvation shell should be subtle. Deactivation to the ground state should also occur activation volume-free with Δ*V*_k_6__^‡^ close to zero (Fig. 7f and S51 in the SI). Charge-separation in ZnP-H⋯C_60_ occurs with a negative Δ*V*_k_3__^‡^ of −5.7 ± 2 cm^3^ mol^−1^, while charge-recombination has a comparable but positive Δ*V*_k_4__^‡^ of +8.6 ± 0.7 cm^3^ mol^−1^ (Fig. 7c and d). A negative activation volume is a result of a pressure-induced acceleration of the charge-separation. This is in accordance with the pressure-induced fluorescence quenching seen for ZnP-H⋯C_60_ (Fig. 4f and Table 1). An accelerated charge-separation leaves a lower ratio of photoexcited ZnP to undergo fluorescent decay.9
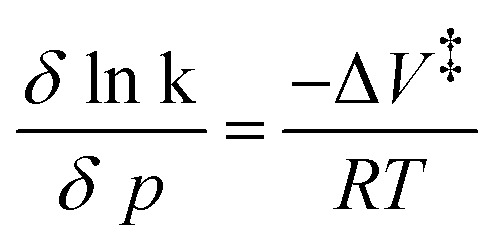


The following factors are considered to evaluate the mechanistic implications of Δ*V*_k_3__^‡^ and Δ*V*_k_4__^‡^. First, ZnP and C_60_ are rigid in nature.^63^ As such, significant bond-length alterations within these electron donors and acceptors are ruled out. A reorganization within the amidinium-carboxylate bridge in the activated state by means of, for example, a proton transfer would result in a neutralization of the charges and to weaker attractive forces across the bridge.^24,41^ One might expect, at this point, longer N⋯H⋯O bond lengths with positive activation volumes. Neutralization of charges across the amidinium-carboxylate bridge implies a lower dipole moment *µ*_*d*_, which is well treated the Jung expression (eqn (5)), and which leads to a volume expansion of the solvation shell.^46,51^ This conflicts, however, with the negative activation volumes seen in the experiments for Δ*V*_k_3__^‡^. Instead, the volume reduction *en route* to the CSS is likely associated with any of the two following factors. First, oxidation of the porphyrin is linked to a lower electron density at the Zn-center and, in turn, a higher affinity to DMAP as donor ligand. The net effect would be a ZnP-DMAP bond length contraction. Second, charge-separation is linked to the formation of the one-electron oxidized form of the porphyrin and the one-electron reduced form of C_60_. Considering that electrostrictive volume differences and radii of the dipolar moiety *r*_*d*_ are inversely proportional to each other, both hypothetically possible reactions, that is, formation of charged ZnP and C_60_ and charge-neutralization within the amidinium-carboxylate bridge, contribute to the observable activation volumes. Oppositely charged ZnP and C_60_ create a dipole moment and, in turn, increase the electrostriction.^46,51^ Stronger interactions with the solvent dipole moments is the direct consequence and this leads to a volume compression following the Jung equation (eqn 5).^46,51^ If both charge-carriers are transferred in a single kinetic step, as, for example, *via* a PCET reaction the opposing volumetric effects will balance out and activation volumes will be negligible.^49^ Considering these contributions in concert, we conclude that the transition state of charge-separation across the amidinium-carboxylate bridge is dictated exclusively by electron transfer. Herby, the charge-separated state (ZnP)˙^+^⋯H⋯(C_60_)˙^−^ is formed without any detectable volumetric contributions stemming from a proton transfer across the amidinium-carboxylate bridge.

The same arguments are applicable for the charge-recombination. Any increase in electron-density at the ZnP-core leads to a lower DMAP affinity and to a ZnP-DMAP bond elongation. Additionally, the neutralization of the opposite charges at the ZnP and C_60_, which is one-electron oxidized and reduced forms, lowers the previous electrostriction and interactions with the solvent dipole moments as described by the Jung equation.^46,51^ Both effects result in the observable positive activation volume Δ*V*_k_4__^‡^. Consequently, effects from a transfer of electrons also govern the activated state as the bottleneck in charge-recombination. Volumetric contributions from a proton transfer remain non-observable.

## Summary

A supramolecular electron donor–acceptor system, consisting of trimesitylene-phenylamidine-zinc porphyrin (ZnP) as electron-donor and ^*n*^octyl-fulleropyrrolidine-benzoic acid (C_60_) as electron-acceptor, was synthesized. Studies necessitated a 1000-fold excess of DMAP to avoid intermolecular ZnP π-stacking. DFT calculations suggest that these building blocks form a charged *η*^2^-binding motif in their ground state, in which both bridging H-atoms are located at ZnP.

Steady-state experiments unveiled a 1 : 1 stoichiometry with a binding constant of 1.9 × 10^5^ M^−1^. In the steady-state absorption experiments, two pressure-induced effects were seen. On one hand, an increase in optical density is linked to a volume compression. On the other hand, a bathochromic shift of the absorption is due to an increasing dielectric constant. Fluorescence experiments similarly showed a subtle bathochromic shift together with a small pressure-induced quenching for ZnP-H⋯C_60_ compared to ZnP.

Pressure-dependent fs-TAS and ns-TAS were carried out to investigate the activation volumes (Δ*V*^‡^) of the excited-state deactivation in ZnP-H⋯C_60_. S_2_, which was populated through photoexcitation into the Soret-band absorption, could not be fully registered due to the 3 ps optical dispersion. Transition from S_1_ towards S_1,Solv_ has a highly positive activation volume. The underlying rate attenuation is linked to a pressure induced viscosity increase. ISC to yield T_1_ and subsequent ground-state deactivation to S_0_ are free of any molecular reorganization and the corresponding activation volumes are essentially zero. Charge-separation is linked to a negative activation volume of −5.7 ± 2 cm^3^ mol^−1^. Charge-recombination has a comparable, but positive activation volume of +8.6 ± 0.7 cm^3^ mol^−1^.

Pressure-dependent reaction kinetics were used to investigate intrinsic as well as electrostrictive volume effects for charge-separation and -recombination in a ZnP-H⋯C_60_ system across an amidinium-carboxylate bridge, by using the Jung equation for dipolar molecules. Volumetric effects in the activated states for either charge-separation or -recombination are mostly determined by electron transfer. Volumetric contributions from a proton movement are non-observable. Consequently, both activation volumes are determined by electron-transfer processes, while effects induced by a proton-transfer during charge-separation and -recombination are absent.

Our presented work highlights the possibility of using pressure-dependent reactions kinetics to investigate the intrinsic and electrostrictive volume effects in pre-organized electron donor–acceptor systems. This extends our previously reported scope of high-pressure kinetics for studying charge-separation and -recombination reactions based on the Drude–Nernst theory for freely-dissolved reactants to covalently/non-covalently pre-organized electron donor–acceptor systems forming dipolar zwitterions as intermediates, based on the Jung theory.^49–51^

## Conclusions

The activation volumes of the observable electron transfer between ZnP and C_60_ across an amidinium-carboxylate bridge occur without any volumetric contributions from a proton transfer within the bridging unit towards the formation of the respective activated states. Congruently, the transition states of charge-separation and -recombination are determined by contributions from electron rather than proton transfer.

## Author contributions

D. L. conducted complexation experiments by means of steady-state absorption and emission spectroscopy. D. L. conducted pressure dependent experiments by means of steady-state absorption, emission, fs- and ns-TA experiments. D. L. main-authored the manuscript. R. W., M. K., C. M. and T. C. contributed with theoretical calculations. R. W. co-authored the manuscript. M. U. synthesized the investigated compounds and characterized them with NMR, MALDI-TOF, ESI-TOF, UV-vis and fluorescence. M. U. co-authored the manuscript. A. Z. contributed with technical support for the pressure equipment. T. T. and D. M. G conceived the project and co-authored the manuscript.

## Conflicts of interest

There are no conflicts to declare.

## Supplementary Material

SC-OLF-D6SC00291A-s001

## Data Availability

Experimental data for this article, including ambient pressure steady state absorption and emission, pressure-dependent steady state absorption and emission, and pressure-dependent transient absorption data are available at Zenodo at https://doi.org/10.5281/zenodo.18648422. Calculation data for this article is available at Zenodo at https://doi.org/10.5281/zenodo.19388636. Supplementary information is available. See DOI: https://doi.org/10.1039/d6sc00291a.
