# Supplementary material for: Activation volumes associated with excited-state electron transfer across amidinium-carboxylate bridge
Source: Chem Sci. 2026 Apr 1;17(21):10479–88. doi: 10.1039/d6sc00291a (PMC13088429; doi:10.1039/d6sc00291a)
Supplement: SC-017-D6SC00291A-s001 [file SC-017-D6SC00291A-s001.pdf]

Supplementary Information (SI) for Chemical Science.
This journal is © The Royal Society of Chemistry 2026

Activation Volumes Associated with Excited-State Electron Transfer Across Amidinium-Carboxylate Bridge

Supporting Information

Daniel Langford,[a] René Weiß,[a,b] Marcel Krug,[a,b] Maxence Urbani,[c] Achim Zahl,[d] Carolin Müller,[b] Timothy Clark,[b] Tomás Torres,*[c,e] Dirk M. Guldi*[a]

[a] FAU Profile Center Solar, Department of Chemistry and Pharmacy & Interdisciplinary Center for Molecular Materials (ICMM), Friedrich-Alexander-Universität Erlangen-Nürnberg, Egerlandstr. 3, 91058 Erlangen, Germany

[b] Department of Chemistry and Pharmacy, Computer-Chemistry Center, Friedrich-Alexander-Universität Erlangen-Nürnberg, Nägelsbachstr. 25, 91052 Erlangen, Germany

[c] Instituto Madrileño de Estudios Avanzados (IMDEA) – Nanociencia, c/ Faraday 9, Madrid 28049, Spain

[d] Department of Chemistry and Pharmacy, Chair of Chemistry of Thin Film Materials, Friedrich-Alexander-University Erlangen-Nürnberg, Cauerstr. 3, 91058 Erlangen, Germany

[e] Departamento de Química Orgánica & Institute for Advanced Research in Chemical Sciences (IAdChem), Universidad Autonoma de Madrid, Spain

Table of Content

1 General Remarks for Optical Spectroscopy Experiments 1

1.1 Ambient Pressure Steady-State Optical Experiments 1

1.2 Transient Absorption Spectroscopy 1

1.3 Pressure Apparatus for Optical Spectroscopy 2

2 General Remarks for Synthesis 3

2.1 Analysis Techniques for Synthesis 3

2.2 Synthesis Scheme 3

2.3 4-(10,15,20-trimesityl-free-base-porphyrin)benzonitrile 5

2.4 4-(10,15,20-trimesityl-zincporphyrin)benzonitrile 6

2.5 4-(10,15,20-trimesityl-zincporphyrin)benzamidinium chloride 7

2.6 4-(10,15,20-trimesityl-zincporphyrin)benzamidine 8

2.7 *N*-Octyl-fulleropyrrolidine-benzoic acid 9

3 NMR Spectra 10

4 Mass Spectra 15

5 Atmospheric Pressure Absorption and Emission Spectra 20

6 Theoretical Calculations 22

7 Pressure Dependent Steady State Experiments 27

8 Pressure Dependent Transient Absorption Spectroscopy 33

9 Literature 38

1 General Remarks for Optical Spectroscopy Experiments

1.1 Ambient Pressure Steady-State Optical Experiments

The steady-state absorption spectra at ambient pressure were recorded with a Perkin Elmer Lambda 2 dual beam absorption spectrometer with 1 nm spectral bandwidth and a scan rate of 300 nm/min. A ZnP concentration of $2.4 \cdot 10^{-6}$ M was used for all titration experiments. The emission spectra were recorded with a FS5 spectrofluorometer from Edinburgh Instruments with 0.3 s integration time, excitation at 567 nm with a slit width of 5 nm and emission slit width of 4 nm. The samples were placed in a 10 × 10 mm quartz cuvette. The fluorescence quantum yields following 567 nm and 430 nm photoexcitation were determined using the SC 30 integration sphere module with the FS5 spectrofluorometer.

1.2 Transient Absorption Spectroscopy

All transient absorption (TA) measurements were carried out on HELIOS femtosecond spectrometer (~200 fs to 5.5 ns) and EOS nanosecond spectrometer (1 ns to 360 μs) from Ultrafast Systems. The light source was a Clark MXR CPA 2110 Ti:Sapphire amplifier with an output of 150 fs light pulses at 775 nm and a repetition rate of 1050 Hz. The excitation pulses at 430 nm, were generated via nonlinear optical pulse amplification. The probe pulse was generated by focusing the 775 nm fundamental into a 2 mm (405 nm–760 nm) or 10 mm (800 nm–1100 nm) sapphire disk. For the nanosecond (ns) experiments, a supercontinuum laser source (370–1200 nm) with a 2100 Hz repetition rate and a pulse width of 1 ns was used.

All fs and ns-TA spectra were fitted using the R-software package TIMP, together with the GloTarAn graphical user interface.[1] Sequential global analysis was applied for the ZnP reference experiments and branched target analysis for the ZnP-H···C_{60} experiments. The applied model is depicted in Figure 6. The respective visible (400-700 nm) and NIR spectra (780-1100 nm) recorded under identical pressure condition were fitted with a common set of kinetic parameters. The natural logarithm of the obtained kinetic values were plotted against the applied pressure in MPa, giving a linear fit. The activation volumes $\Delta V^{\ddagger}$ were calculated according to the following equation with a constant value for T of 293 K:

$$\Delta V^{\ddagger} =- [slope] \cdot R \cdot T$$

1.3 Pressure Apparatus for Optical Spectroscopy

An in-house developed setup was used for recording pressure dependent steady-state spectra (see Figure S1.1 in the SI).[2] For absorption measurements, Avantes AvaLight DH-S-BAL white light source was coupled into an optical fiber and aligned to the center of the optical windows of the pressure cell. The transmitted light was coupled into an optical fiber and directed to the Avantes AvaSpec UV-Vis spectrometer. For pressure-dependent emission measurements, the light pulses at 430 nm from the Clark MXR CPA 2110 after non-linear optical pulse amplification was used for exciting the sample. The excitation light was filtered out after the pressure cell with a 550 nm long pass filter and the transmitted light was coupled into an optical fiber connected to an Avantes AvaSpec UV-Vis spectrometer.

A cuvette with an optical pathlength of 6 mm and a ZnP concentration of $5 \cdot 10^{-6}$ M was used for all pressure-dependent experiments. The pressure apparatus was equipped with the sample and pressurized to 5 MPa. The system was continuously checked for sudden drop of pressure and leakage at the joints. The pressure was typically changed with 30 MPa steps up to 120 MPa and left for equilibration before the measurement was started. All systems were measured once on the pressurization direction and depressurization direction for checking the reversibility of the pressure effect on the spectra and kinetics.

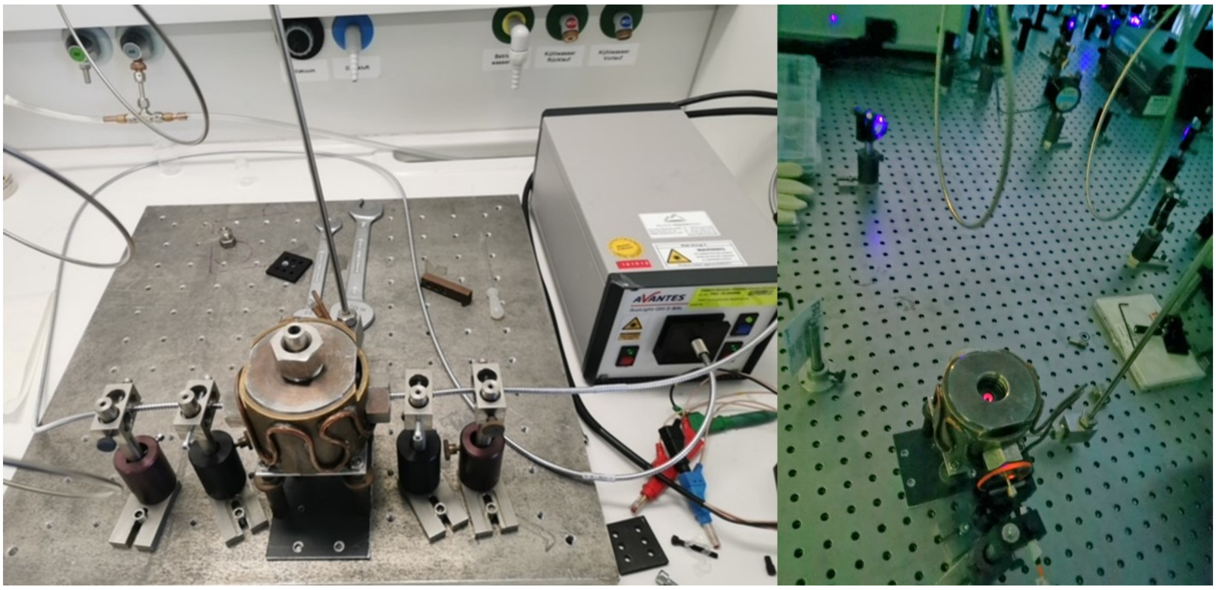

Figure S1. Experimental setup for measuring pressure-dependent steady state absorption (left) and emission (right). The applied procedure is described in the methods section in the main manuscript. The sample was placed in the pressure cell through the top screw. The hydrostatic pressure was transferred to the cell through the vertical tube on the back side of the cell with water as the pressure medium.

2 General Remarks for Synthesis

2.1 Analysis Techniques for Synthesis

^{1}H (400 MHz) and ^{1}H-decoupled ^{13}C (100 MHz) NMR spectra were recorded on a Bruker TopSpin AV-400 spectrometer at 298 K. Chemical shifts are reported in parts per million (ppm) relative signal(s) of the partially deuterated solvents as internal standards. Multiplicities are denoted as follows: s = singlet, d = doublet, dd = doublets of doublet, t = triplet, m = multiplet, and the coupling constants (J_H-J_H) are given in Hz.

MS experiments were performed by the Servicio Interdepartamental de Investigacion (SIdI) at the Autonoma University of Madrid. Matrix assisted laser desorption ionization time of flight (MALDI-TOF MS) experiments were recorded on a Bruker Ultraflex III. High resolution electrospray ionization time of flight (HR-ESI-TOF) mass spectra were recorded on a QSTAR-TOF hybrid mass spectrometer.

2.2 Synthesis Scheme

Synthetic procedures were carried out under an inert argon atmosphere, in dry solvents unless otherwise noted. Anhydrous solvents used for reaction (reagent grade; Scharlau) were used as received, except chlorobenzene that was dried over molecular sieves (3 Å) and kept under argon prior to use. Reagents (Reagent grade, Alfa Aesar and Sigma-Aldrich), deuterated solvents for NMR (Sigma-Aldrich) and solvents for chromatography columns and purifications (ISO reagent grade, Scharlau) were used as purchased. Pyrole (Sigma-Aldrich) was passed through a plug of aluminum oxide (activated, neutral) prior to use. Chromatographic purifications were performed using silica gel 60, 0.040–0.06 mm (230-400 mesh ASTM; Scharlau). Analytical thin-layer chromatography (TLC) was performed using aluminium coated Merck Kieselgel 60 F254. Synthesis and characterization of ZnP[3], and N-Octyl-fulleropyrrolidin-benzoic acid[4] were previously reported elsewhere in the literature and synthetized according to modified procedures.

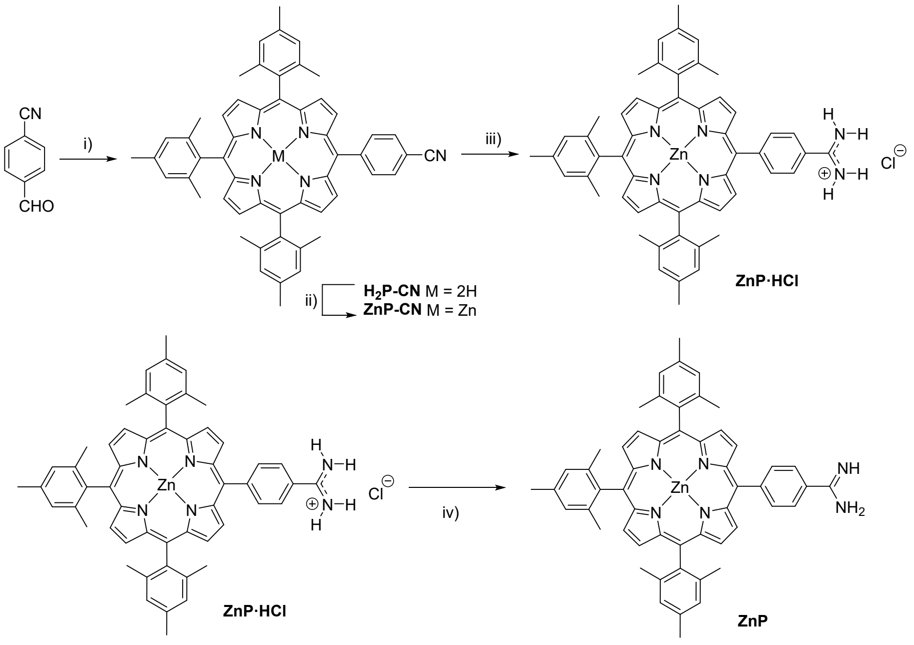

Scheme S1. Synthetic route to amidino-porphyrins ZnP. Reagents and conditions: i) mesitaldehyde, pyrrole, BF_3•Et_2O, $CHCl_3$, rt, 3 h, followed by DDQ, rt, 1 h (19.5%). ii) $Zn(OAc)_2$, $CHCl_3$/MeOH (9:1), reflux, 2 h (91%). iii) $AlCl(Me)NH_2$, toluene, 80 ºC, 3 days (73%). iv) NaOH 0.1 M, $CHCl_3$/MeOH (20:1), rt, 30 min then extraction with $CHCl_3$ (quantitative).

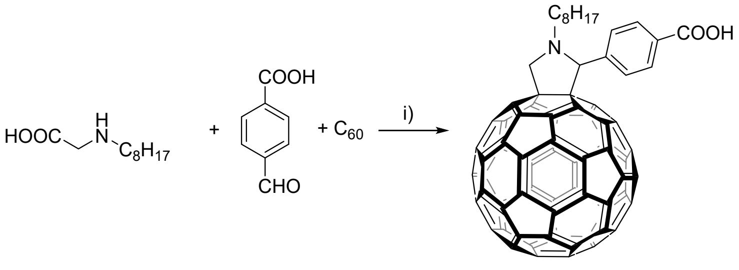

2

Scheme S2. Synthesis of N-Octyl-fulleropyrrolidine-benzoic acid. Reagents and conditions: chlorobenzene, reflux, 16 h (71%).

2.3 4-(10,15,20-trimesityl-free-base-porphyrin)benzonitrile

Synthesized according to modified procedures previously described.[3] A solution of cyanobenzaldehyde (590.5 mg, 4.51 mmol) and mesitaldehyde (2 mL; 2.01 g, 13.6 mmol) and pyrrole (1.22 g, 18.2 mmol) in $CHCl_3$ containing 0.75% of EtOH (1.8 L) was degassed by bubbling argon for 30 min. Afterwards, a 7.93 M solution of BF_3•Et_2O in Et_2O (0.75 mL, 5.95 mmol) was added and the resulting mixture stirred for 3 h at rt under argon. DDQ (3.06 g, 2.24 mmol) was then added and the mixture stirred for an additional hour. Finally, few drops of Et_3N were added to neutralize the acidic medium. The mixture was then filtered through a plug of SiO_2 ($CHCl_3$) and the solvents evaporated. The resulting solid was purified by gel permeation chromatography (Biobeds, Biorads SX-1; eluent: THF), followed by column chromatography on SiO_2 ($CHCl_3$/hexanes, 1:1), to afford H_2P-CN (677 mg, 0.88 mmol) as a dark purple solid (Yield 19.5%).

^{1}H NMR (400 MHz, $CDCl_3$, 25 °C) δH = 8.96 (d, $^3J(\beta_1,\beta_2)$ = 4.8 Hz, 2H; H-β_1), 8.91–8.87 (m (bs+d), 6H; H-β_{2-4}), 8.53 (d, ^3J(X,Y) = 8.2 Hz, 2H; H-*X*), 8.22 (d, ^3J(Y,X) = 8.2 Hz, 2H; H-*Y*), 7.48 (s, 4H; H-*R*), 7.47 (s, 2H; H-*R'*), 2.81 (s, 6H; H-a; ArC*H*$_3$), 2.80 (s, 3H; H-a'; ArC*H*$_3$), 2.09 (s, 6H; H-b'; ArC*H*$_3$), 2.09 (s, 12H; H-b; ArC*H*$_3$), -2.30 ppm (s, 2H; N*H*).

^{13}C and DEPT135 NMR (100 MHz, $CDCl_3$, 25°C) δ_C= 153.8–141.6 (br; α–*Cpyrrolic*), 147.3, 139.4, 138.2, 138.0, 137.9, 134.9 (Ar*C*H), 130.5 (Ar*C*H), 127.9 (Ar*C*H), 119.1, 118.6, 118.5, 116.4, 111.8, 21.8 (Ar*C*H_3), 21.7 (Ar*C*H_3), 21.5 ppm (Ar*C*H_3).

MS (MALDI-TOF; *DCTB*): *m/z* (%) found: 765.4 (100) $[M]^+$.

HRMS (MALDI-TOF; DCTB + PPGNa 790): *m/z* calc. for $C_{54}H_{47}N_5$: 765.3826; found 765.3837 $[M]^+$.

UV/Vis (THF; 25°C): λ_{max} (ε)= 417 (358 400), 513 (15 940), 546 (5 630), 591 (4 540), 647 nm (3 130 dm^3•mol^{-1}•cm^{-1}).

Fluorescence (2.71×10^{-6} mol/L in THF; $\lambda_{exc.}$= 417 nm; 25°C): $\lambda_{max,em.}$ = 651, 719 nm.

Data is consistent with those previously reported for this compound.[3]

2.4 4-(10,15,20-trimesityl-zincporphyrin)benzonitrile

Synthesized according to a modified procedure previously described.[3] A solution of H_2P-CN (303.5 mg, 0.396 mmol) and anhydrous $Zn(OAc)_2$ (363 mg, 1.98 mmol) in a 9:1 mixture of $CHCl_3$ (18 mL) and MeOH (2 mL) was heated to reflux for 2 h. After cooling to rt, few drops of Et_3N were added, and then solvents evaporated to dryness. The crude residue was purified by column chromatography on SiO_2 ($CHCl_3$/hexanes, 1:1 + 0.1% Et_3N) to afford ZnP-CN (299.6 mg, 0.361 μmol) as a dark pink solid (Yield 91%).

^{1}H NMR (400 MHz, $CDCl_3$, 25°C) δ_H = 8.82 (d, $^3J(\beta_1,\beta_2)$ = 4.6 Hz, 2H; H-β_1), 8.76 (d, $^3J(\beta_2,\beta_1)$ = 4.6 Hz, 2H; H-β_2), 8.75 (d, $^3J(\beta_3,\beta_4)$ = 4.6 Hz, 2H; H-β_3), 8.74 (d, $^3J(\beta_4,\beta_3)$ = 4.6 Hz, 2H; H-β_4), 8.38 (d, ^3J(X,Y) = 8.2 Hz, 2H; H-*X*), 8.06 (d, ^3J(Y,X) = 8.2 Hz, 2H; H-*Y*), 7.30 (s, 4H; H-*R*), 7.31 (s, 2H; H-*R'*), 2.65 (s, 6H; H-a; ArCH_3), 2.66 (s, 3H; H-a'; ArCH_3), 1.88 (s, 6H; H-b'; ArCH_3), 1.87 ppm (s, 12H; H-b; ArCH_3).

^{13}C NMR (100 MHz, $CDCl_3$, 25°C) δ_C= 150.2, 150.0, 149.7, 148.9, 148.2, 139.2, 138.9, 138.8, 137.5, 134.9, 131.4, 131.3, 131.2, 131.1, 130.3, 127.7, 119.2, 117.2, 111.4, 21.8 (Ar*C*H_3), 21.7 (Ar*C*H_3), 21.5 ppm (Ar*C*H_3).

MS (MALDI-TOF; *DCTB*): *m/z* (%) found: 827.3 (100) $[M]^+$.

HRMS (MALDI-TOF; DCTB + PPGNa 790): *m/z* calc. for $C_{54}H_{45}N_5Zn$: 827.2961; found 827.2954 $[M]^+$.

UV/Vis (THF; 25°C): λ_{max} (ε) = 425 (573 200), 557 (19 900), 597 nm (4 900 dm^3•mol^{-1}•cm^{-1}).

Fluorescence (1.51×10^{-6} mol/L in THF; $\lambda_{exc.}$= 420 nm; 25°C): $\lambda_{max,em.}$ = 605, 655 nm.

Data is consistent with those previously reported for this compound.[3]

2.5 4-(10,15,20-trimesityl-zincporphyrin)benzamidinium chloride

Synthesized according to modified procedures previously reported.[5,6] A freshly prepared $AlCl(CH_3)(NH_2)$ solution (from addition of a 2 M $AlMe_3$ solution in toluene (7 mL; 0.140 mmol) over NH_4Cl (750 mg 0.140 mmol)) was added dropwise and under argon to a solution of ZnPor-CN (89.6 mg, 0.108 mmol) in dry toluene (100 mL). The solution was heated at 80 °C for 3 days. After cooling to rt, SiO_2 (*ca.* 10 g) was added, and the mixture stirred for 15 min. Hexane (100 mL) was added and the mixture filtrated over a plug of SiO_2. The SiO_2 purple filtrate was washed with $CHCl_3$ as eluent until the disappearance of the remaining starting material (ZnP-CN), followed by a 2:1 mixture of $CHCl_3$ and MeOH to eluate the desired product. Purification was achieved by chromatography column on SiO_2 ($CHCl_3$/MeOH, 10:0.5 to 8:2) to afford ZnP•HCl(70.0 mg, 0.079 mmol) as a dark pink solid (Yield 73%).

^{1}H NMR (400 MHz, MeOD-[d4], 25°C) δ_H = 8.72 (d, $^3J(\beta_1,\beta_2)$ = 4.6 Hz, 2H; H-β_1), 8.65 (d, $^3J(\beta_2,\beta_1)$ = 4.6 Hz, 2H; H-β_2), 8.60 (d, $^3J(\beta_3,\beta_4)$ = 4.8 Hz, 2H; H-β_3), 8.59 (d, $^3J(\beta_4,\beta_3)$ = 4.8 Hz, 2H; H-β_4), 8.45 (d, ^3J(X,Y) = 8.3 Hz, 2H; H-*X*), 8.19 (d, ^3J(Y,X) = 8.3 Hz, 2H; H-*Y*), 7.24 (s, 4H; H-*R*), 7.22 (s, 2H; H-*R'*), 2.56 (s, 6H; H-a; ArCH_3), 2.54 (s, 3H; H-a'; ArCH_3), 1.82 ppm (s, 18H; H-b+H-b'; ArCH_3).

^{13}C and DEPT-135 NMR (100 MHz, $CDCl_3$, 25°C): δ_C= 168.8 ($C(NH_2)_2^+$), 151.5, 151.4, 151.2, 151.1, 150.5, 140.8, 140.8, 140.4, 138.7, 136.3 (Ar*C*H), 132.1(Ar*C*H), 132.0 (Ar*C*H), 131.8 (Ar*C*H), 131.6 (Ar*C*H), 128.6 (Ar*C*H), 128.4, 127.1 (Ar*C*H), 120.0, 119.9, 118.4, 22.1 (Ar*C*H_3), 22.0 (Ar*C*H_3), 21.6 ppm (Ar*C*H_3).

HRMS (ESI$^+$; matrix: MeOH): *m/z* (%) calc. for $C_{54}H_{49}N_6Zn$: 845.3305; found 845.3283 (100) $[M-Cl]^{+\bullet}$.

UV/Vis (THF; 25°C): λ_{max} (ε) = 425 (527 500), 558 (18 630), 597 nm (5 140 $dm^3 \cdot mol^{-1} \cdot cm^{-1}$).

Fluorescence (1.51×10^{-6} mol/L in THF; $\lambda_{exc.}$= 420 nm; 25°C): $\lambda_{max,em.}$ = 604, 655 nm.

2.6 4-(10,15,20-trimesityl-zincporphyrin)benzamidine

To a solution of ZnP•HCl (106 mg, 0.12 mmol) in $CHCl_3$ (20 mL) and MeOH (1 mL), was added a 0.1 M NaOH solution in H_2O (30 mL). The mixture was vigorously stirred for 30 min then transferred into a separation funnel. After phase separation, the aqueous phase was extracted twice with $CHCl_3$. The organic phases were combined, dried over Na_2SO_4, filtrated, and evaporated to dryness. Purification was achieved by precipitation with *n*-pentane from a $CHCl_3$ solution (minimum amount), to afford ZnP (102 mg, 0.12 mmol) as dark pink solid (Yield: quantitative).

^{1}H NMR (400 MHz, MeOD-[d4], 25°C): δ_H = 8.69 (d, $^3J(\beta_1,\beta_2)$ = 4.6 Hz, 2H; H-β_1), 8.64 (d, $^3J(\beta_2,\beta_1)$ = 4.6 Hz, 2H; H-β_2), 8.61 (d, $^3J(\beta_3,\beta_4)$ = 4.6 Hz, 2H; H-β_3), 8.60 (d, $^3J(\beta_4,\beta_3)$ = 4.6 Hz, 2H; H-β_4), 8.37 (d, ^3J(X,Y) = 8.2 Hz, 2H; H-*X*), 8.09 (d, ^3J(Y,X) = 8.2 Hz, 2H; H-*Y*), 7.27 (s, 6H; H-(*R*+*R'*)), 2.60 (s, 9H; H-(a+a'); ArCH_3), 1.86 (s, 6H; H-b'; ArCH_3), 1.84 ppm (s, 12H; H-b; ArCH_3).

^{13}C NMR (100 MHz, $CDCl_3$, 25°C) δ_C= 168.6 (C=NH(NH_2)), 151.4, 151.2, 151.1, 150.6, 140.9, 140.8, 140.4, 138.7, 136.1, 132.2, 131.9, 131.8, 131.5, 128.6, 126.8, 119.9, 119.8, 118.7, 22.1 (Ar*C*H_3), 22.0 (Ar*C*H_3), 21.6 ppm (Ar*C*H_3).

MS (MALDI-TOF; *DCTB*): *m/z* (%) found: 844.3 (100) $[M]^+$.

HRMS (MALDI-TOF; DCTB + PPGNa 790): *m/z* calc. for $C_{54}H_{48}N_6Zn$: 844.3226; found 844.3218 $[M]^+$.

UV/Vis (THF; 25°C): λ_{max} (ε) = 425 (451 700), 557 (18 200), 599 nm (6 540 $dm^3•mol^{-1}•cm^{-1}$).

Fluorescence (1.65×10^{-6} mol/L in THF; $\lambda_{exc.}$= 420 nm; 25°C): $\lambda_{max,em.}$ = 604, 655 nm.

2.7 *N*-Octyl-fulleropyrrolidine-benzoic acid

This compound was synthetized and purified according to modified procedures previously reported for this compound in the literature.[4] A solution of C_{60} (351 mg, 0.487 mmol), 4-carboxybenzaldehyde (62.5 mg, 0.416 mmol) and N-octylglycine (312 mg, 0.167 mmol) was degassed with Ar through a needle/septum for 30 min. Afterwards, the solution was kept under argon and heated to reflux for 16 h. After cooling to rt, PhCl was removed under vacuum by rotary evaporation. The crude residue was dissolved in CS_2 and passed through a plug of silica, using first CS_2 as eluent to remove the excess of unreacted pristine C_{60}, then $CHCl_3$/MeOH 8:2 to recover the different products. The residue was then purified by gel permeation chromatography (Biodeds SX-1, Biorad; eluent: $CHCl_3/CS_2$ 1:1), followed by chromatography column on SiO_2 (eluent: $CS_2/CHCl_3$ 1:1 + 0.25% MeOH) to afford compound 3 (118 mg; 0.118 mmol) as a brown powder (racemic form; Yield: 71%).

^{1}H NMR (400 MHz, $CS_2/CDCl_3$ (1:1), 25°C): δ_H = 8.17 (d, ^3J = 8.4 Hz, 2H; H-*Y*, Ph*H*), 7.95 (bs, 2H; H-*X*, Ph*H*), 5.16 (s, 1H; H-*b,* NC*H*), 5.15 (d, ^2J(a-a') = 9 Hz, 1H; H-*a*, NC*H*H'), 4.17 (d, ^2J(a-a') = 9 Hz, 1H; H-*a'*, NCH*H'*), 3.22 (td, $^2J(c\text{-}c')$ = 12 Hz, $^3J(c\text{-}d)$ = 8.4 Hz, 1H; H-c, αC*H*H'), 2.61 (td, $^2J(c\text{-}c')$ = 12 Hz, $^3J(c'\text{-}d)$ = 8.4 Hz, 1H; H-c', αCH*H'*), 2.08–1.97 (m, 1H; H-d; βCH*H'*), 1.97–1.85 (m, 1H; H-d'; βCH*H'*), 1.75–1.62 (m, 1H; H-e, γC*H*H'), 1.62–1.52 (m, 1H; H-e', γCH*H'*), 1.52–1.29 (m, 8H; H-f→i; CH_2), 0.95 (t, ^3J = 6.8 Hz; 3H; H-j, CH_3).

MS (MALDI-TOF; DCTB + NaI): *m/z* (%) found: 994.2 (39) $[M\text{-}H]^{+\bullet}/[M]^{+\bullet}$, 1018.2 (28) $[M+Na]^{+\bullet}$ 1040.1 (100) $[M\text{-}H+2Na]^{+\bullet}$.

HRMS (MALDI-TOF; DCTB + NaI + PPGNa 790): *m/z* calcd for $C_{77}H_{24}NO_2Na$: 1040.1597; found 1040.1619 $[M\text{-}H+2Na]^{+\bullet}$.

UV/Vis (THF; 25°C): λ_{max} (ε) = 307 (sh; 33 570), 325 (br sh, 31 900), 430 nm (35 320 $dm^3 \bullet mol^{-1} \bullet cm^{-1}$).

Data is consistent with those previously reported for this compound.[4]

3 NMR Spectra

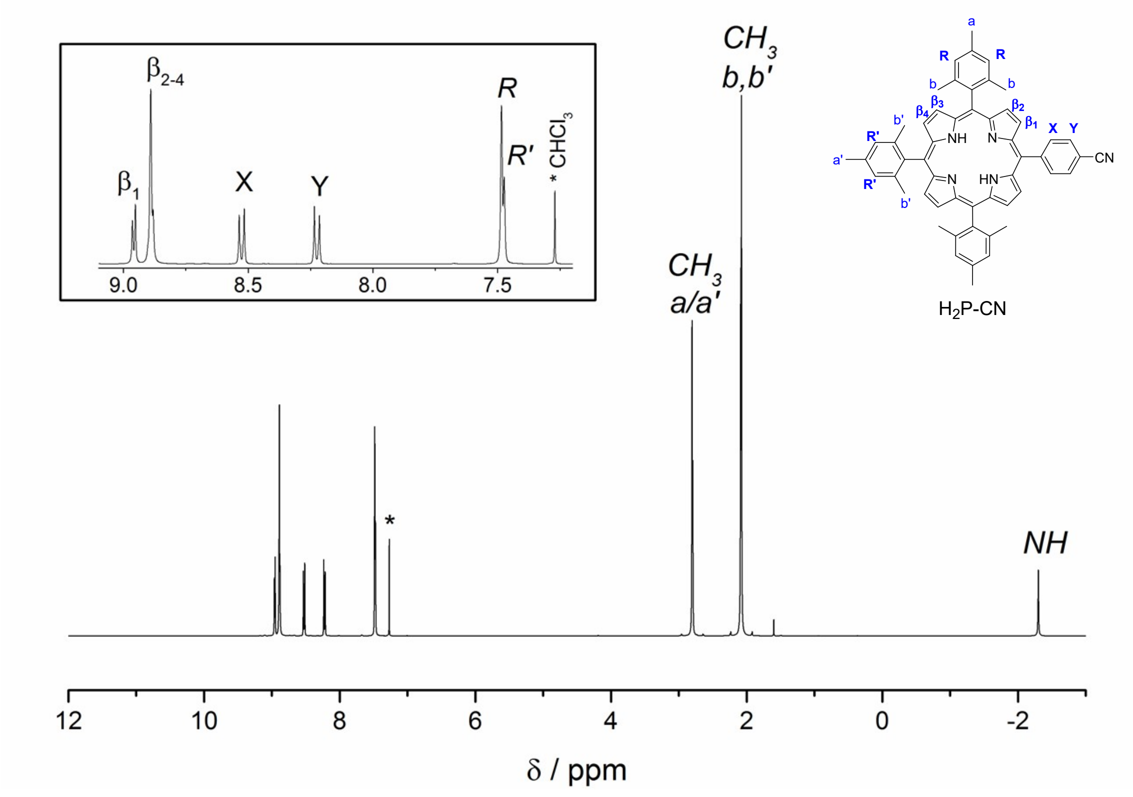

Figure S2. ^{1}H NMR (400 MHz; $CDCl_3$) of H_2P-CN (* denotes the signal of residual $CHCl_3$ from the $CDCl_3$ solvent).

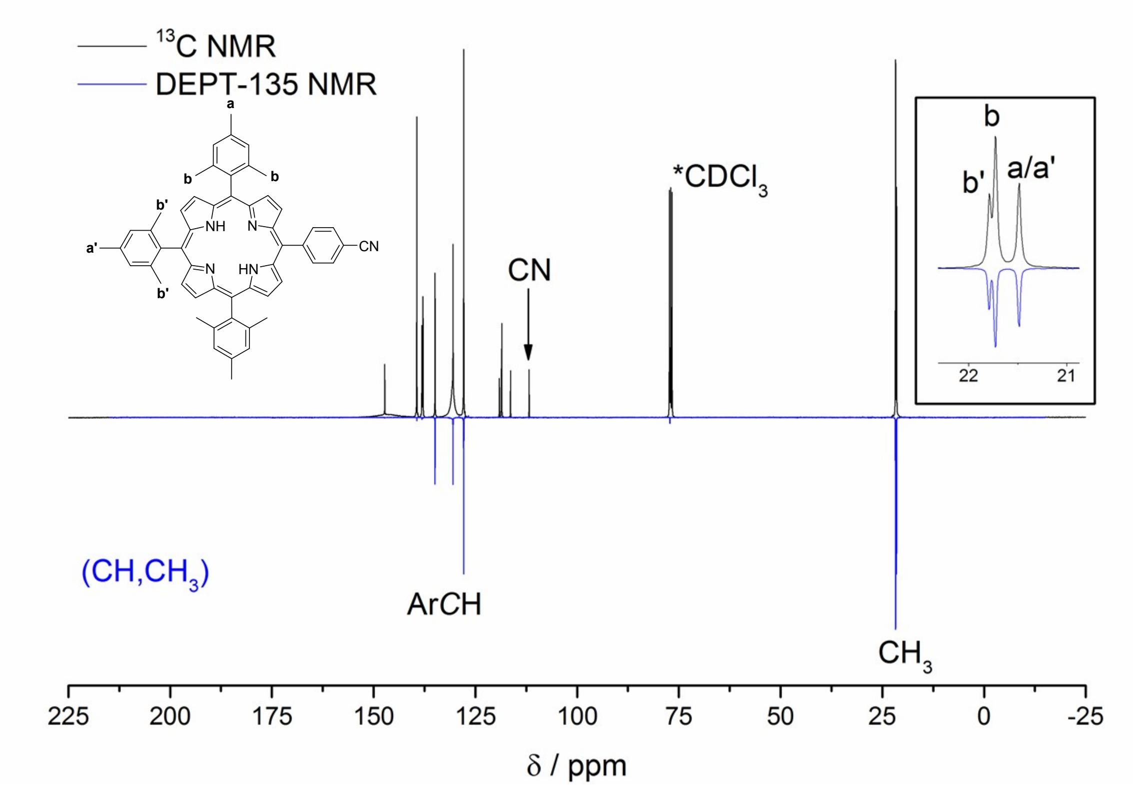

Figure S3. ^{13}C- and DEPT 135- NMR spectra (100 MHz; $CDCl_3$) of H_2P-CN (* denotes the triplet signal of the $CDCl_3$ solvent). The DEPT 135 spectrum was inverted for better comparability with the ^{13}C spectrum.

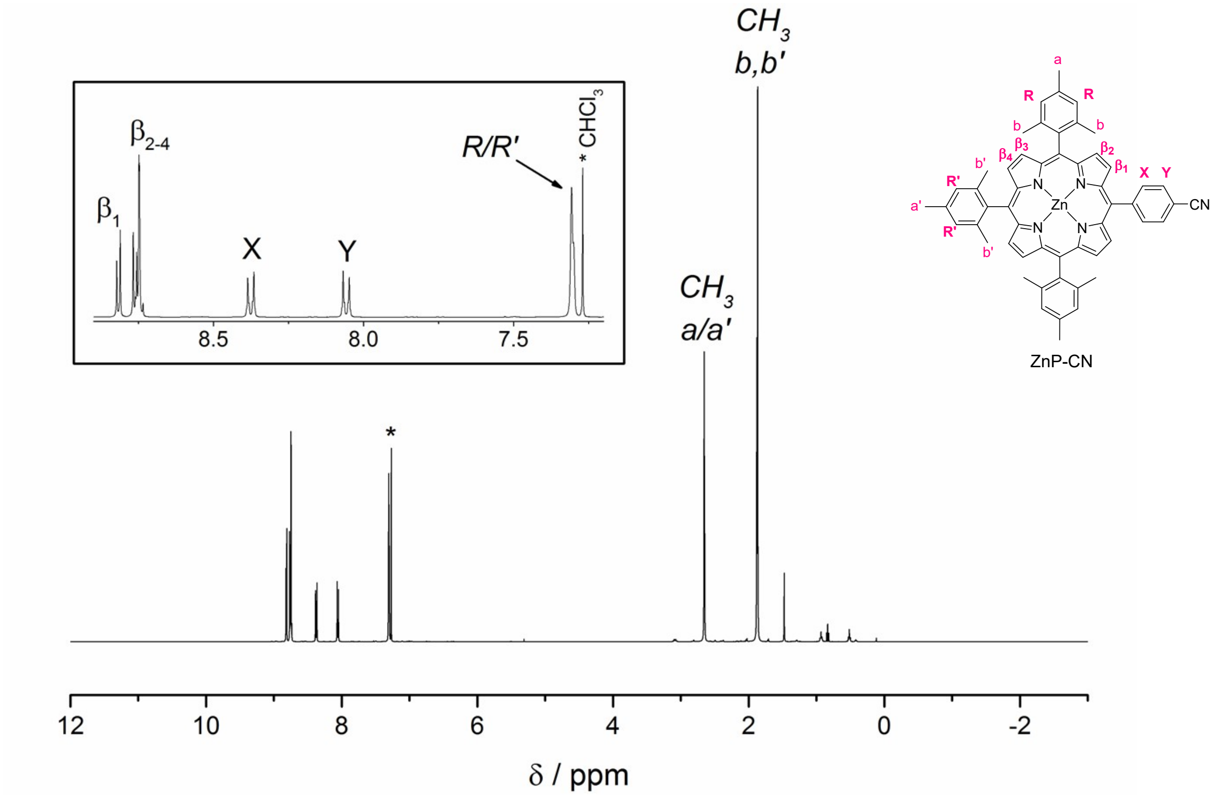

Figure S4. ^{1}H NMR (400 MHz; $CDCl_3$) of ZnP-CN (* denotes the signal of residual $CHCl_3$ from the $CDCl_3$ solvent).

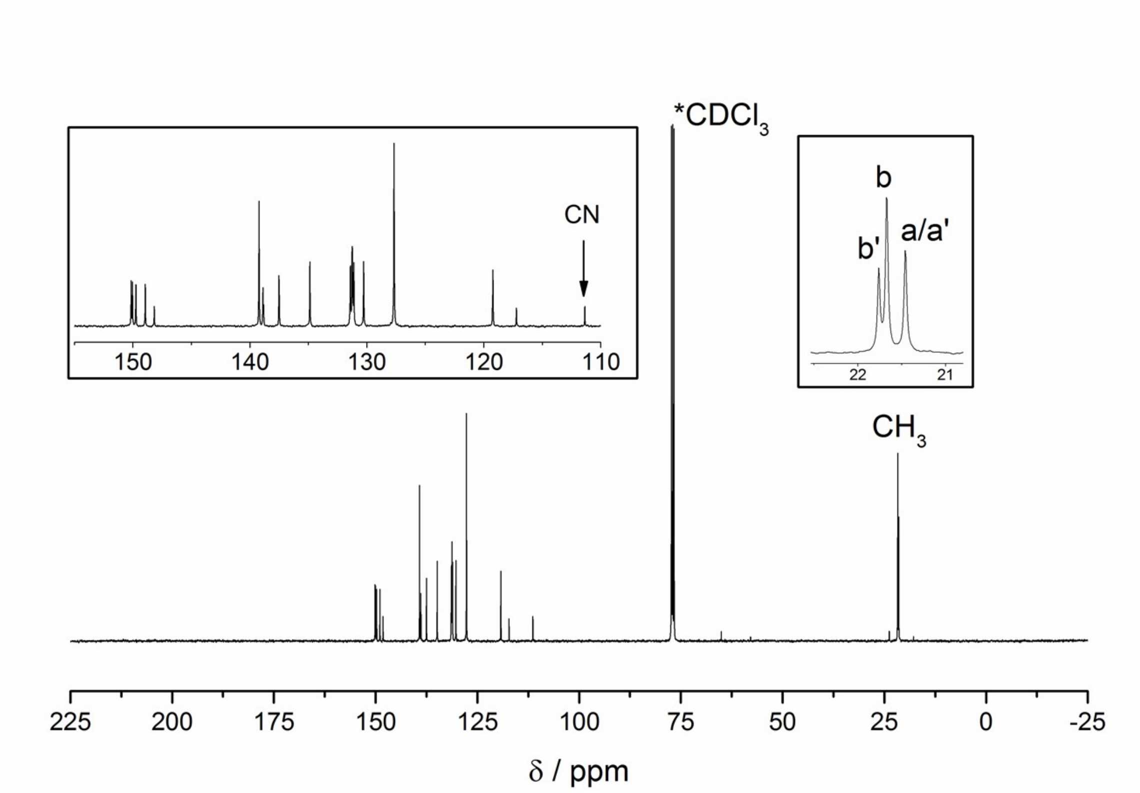

Figure S5. ^{13}C NMR (100 MHz; $CDCl_3$) of ZnP-CN (* denotes the triplet signal of the $CDCl_3$ solvent).

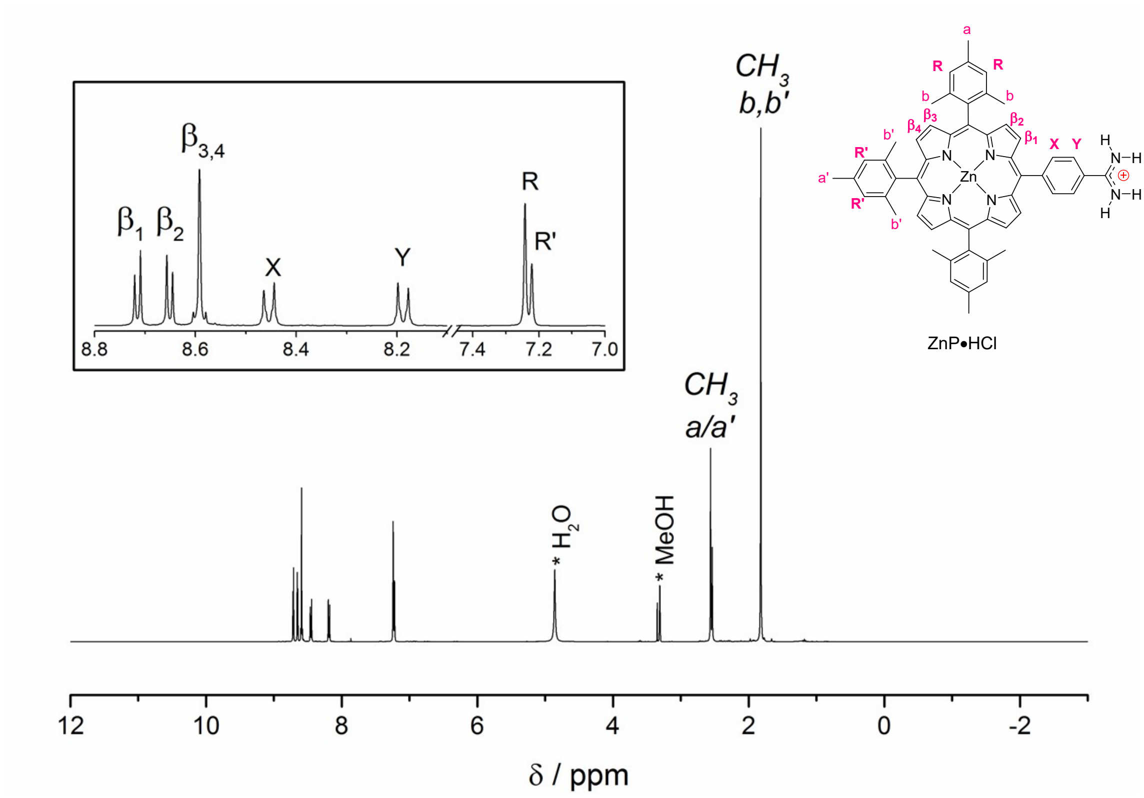

Figure S6. ^{1}H NMR (400 MHz; MeOD-d4) of ZnP•HCl (* denotes the signals of residual MeOH and H_2O from the MeOD-d4 solvent).

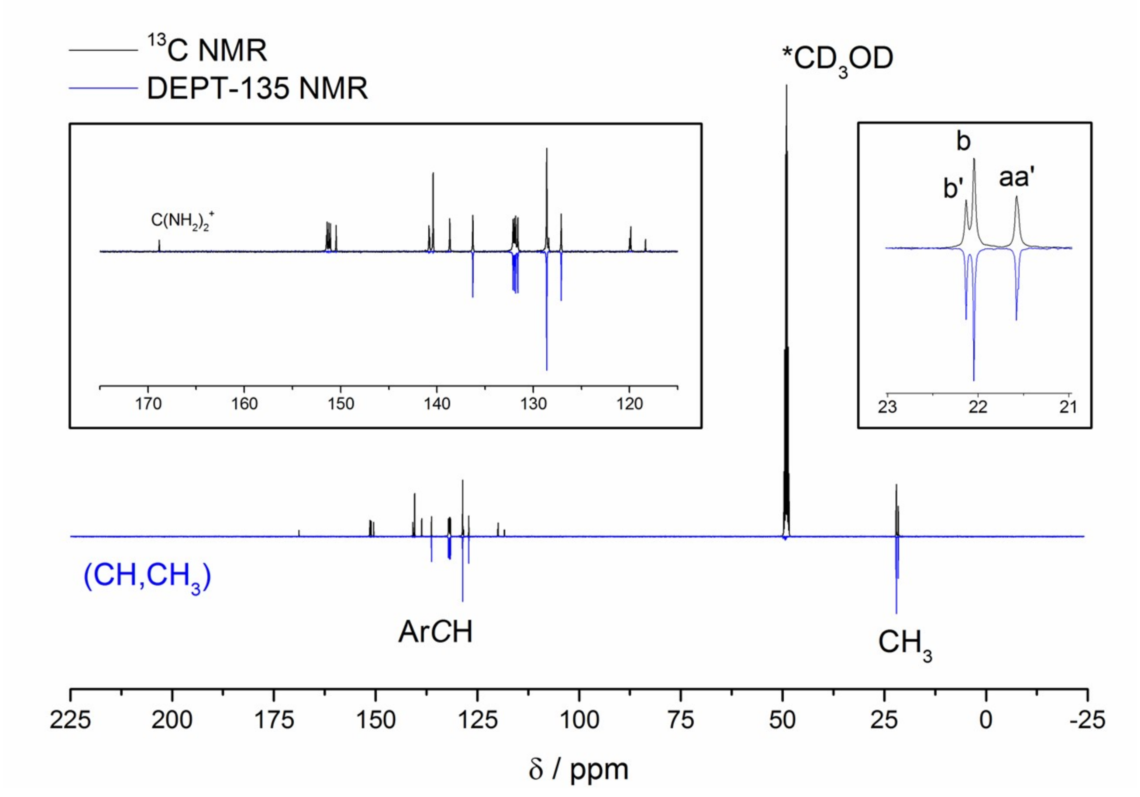

Figure S7. ^{13}C- and DEPT 135-NMR spectra (100 MHz; MeOD-d4) of ZnP•HCl (*denotes the septuplet signal of the MeOD-d4 solvent). The DEPT 135 spectrum was inverted for better comparability with the ^{13}C spectrum.

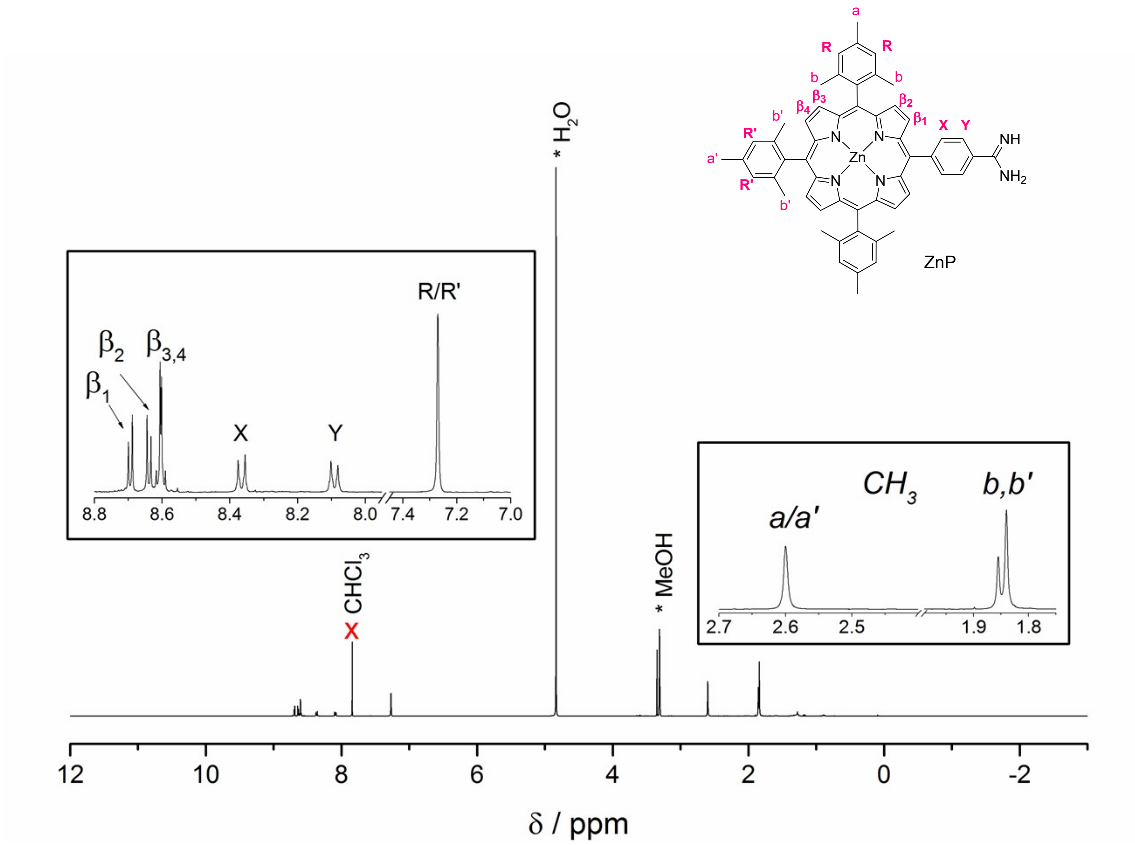

Figure S8. ^{1}H NMR (400 MHz; MeOD-d4) of ZnP (*denote the signals of residual MeOH and H_2O from the MeOD-d4 solvent; “x” denotes to a signal of residual $CHCl_3$ solvent from purification).

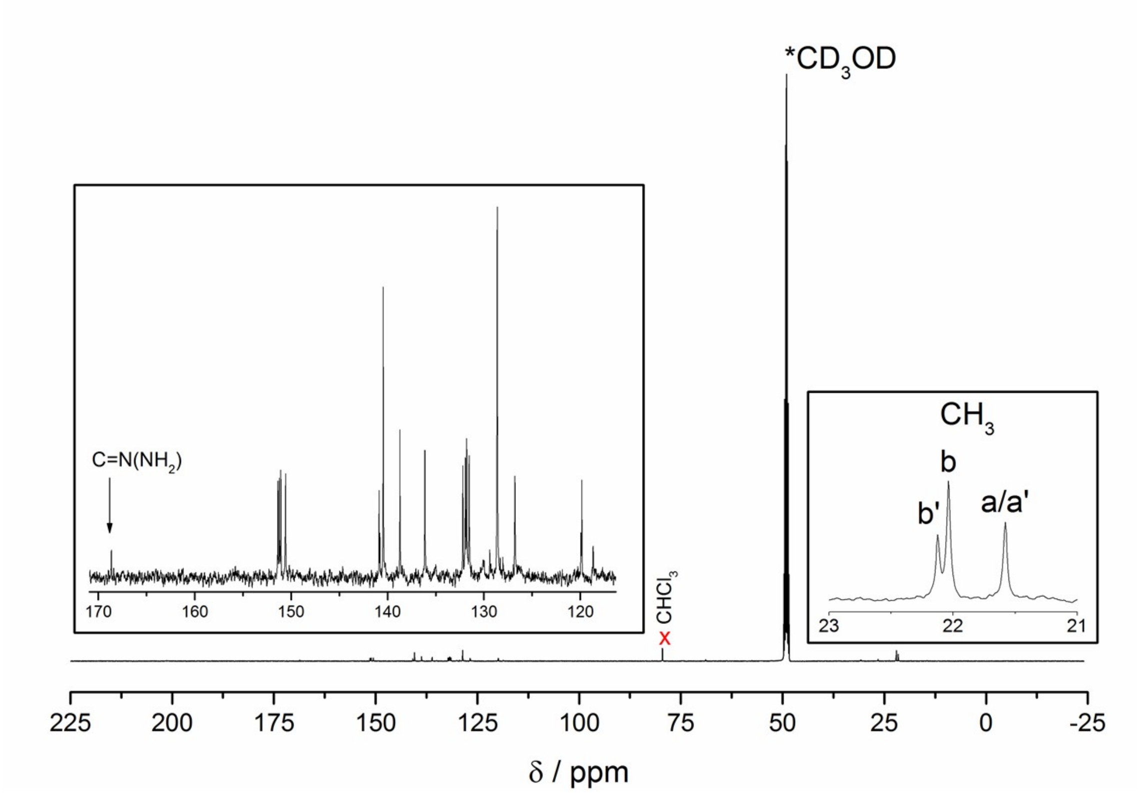

Figure S9. ^{13}C NMR spectrum (100 MHz; MeOD-d4) of ZnP (*denotes the septuplet signal of the MeOD-d4 solvent; “x” denotes the signal of residual $CHCl_3$ solvent from purification).

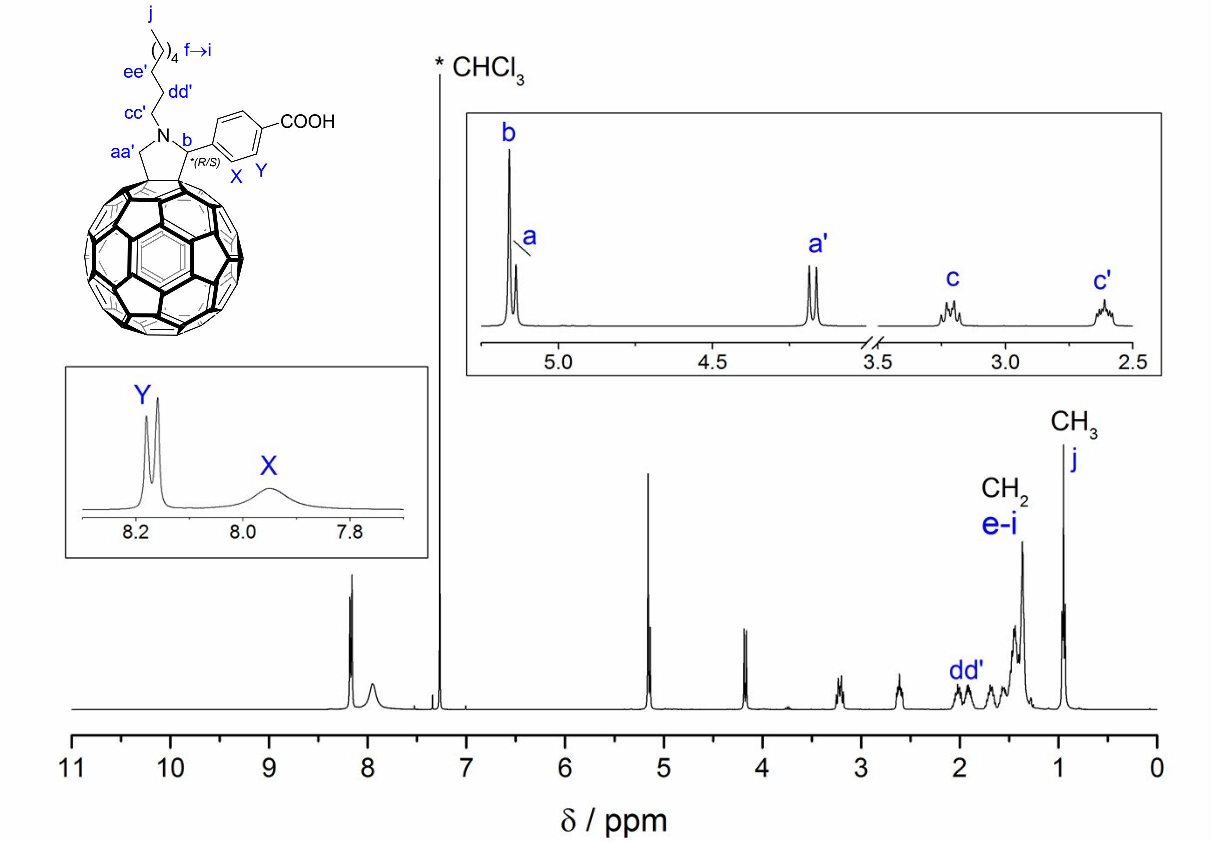

Figure S10. ^{1}H NMR (400 MHz; $CS_2/CDCl_3$ (1:1) spectrum of *N*-Octyl-fulleropyrrolidine-benzoic acid.

4 Mass Spectra

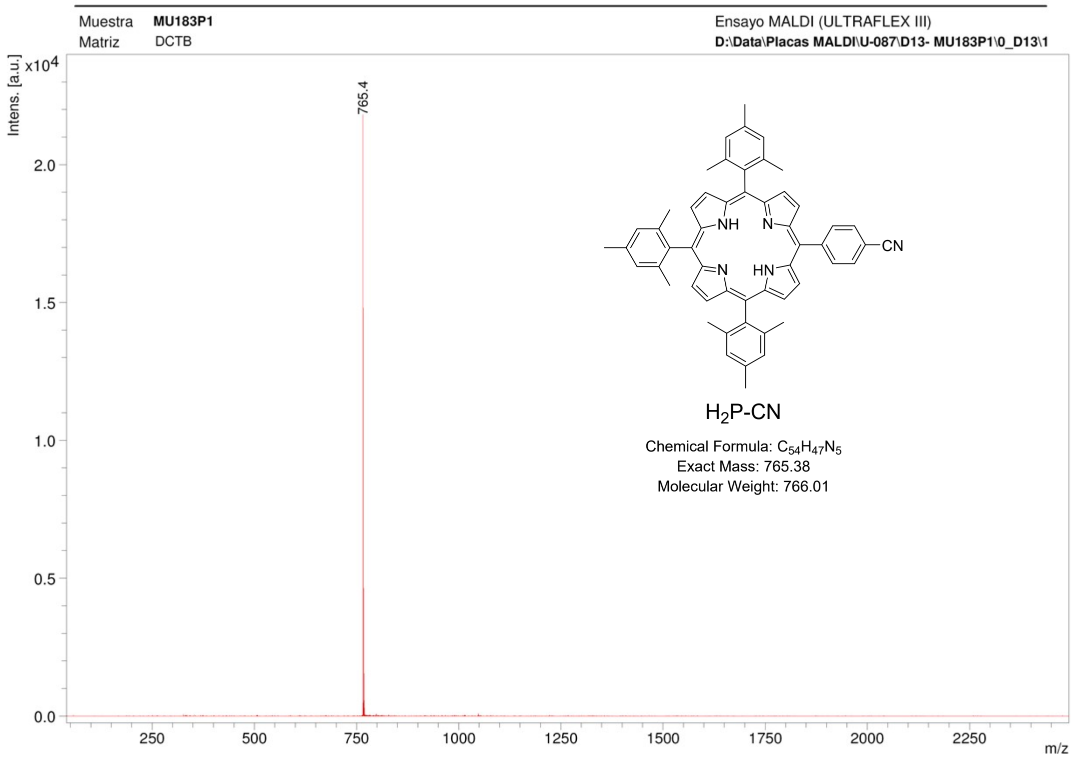

Figure S11. MS spectrum of H_2P-CN (MALDI-TOF$^+$; Matrix: DCTB).

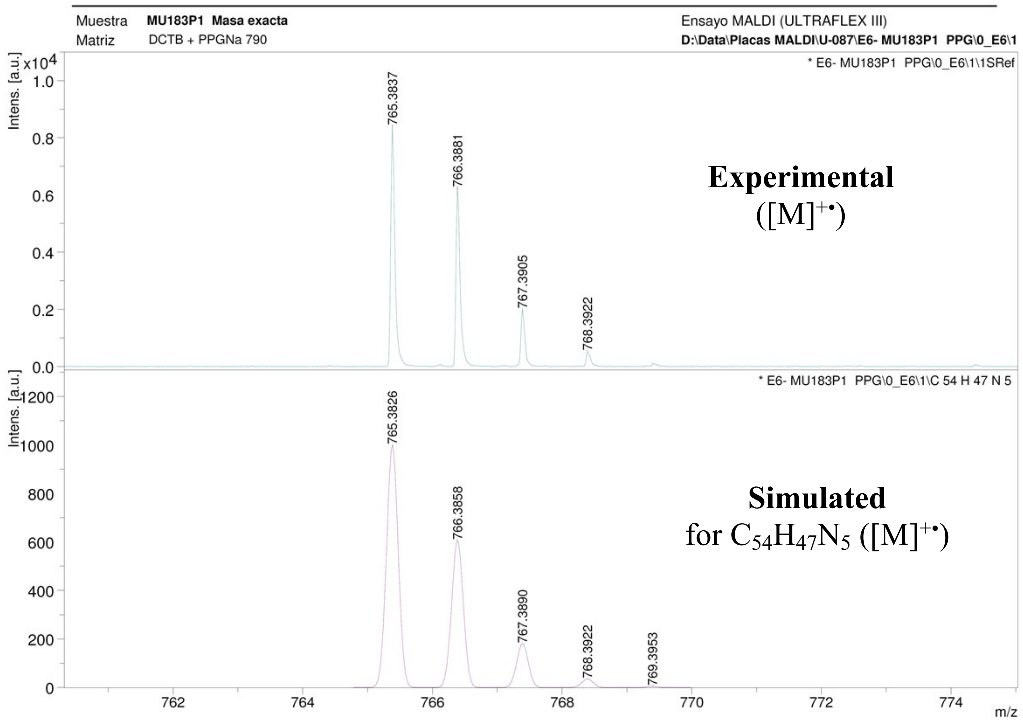

Figure S12. HRMS spectrum of H_2P-CN (top inset; MALDI-TOF$^+$; Matrix: DCTB + PPGNa 790) and isotopic distribution simulated for $[M]^{+\bullet}$ (bottom inset).

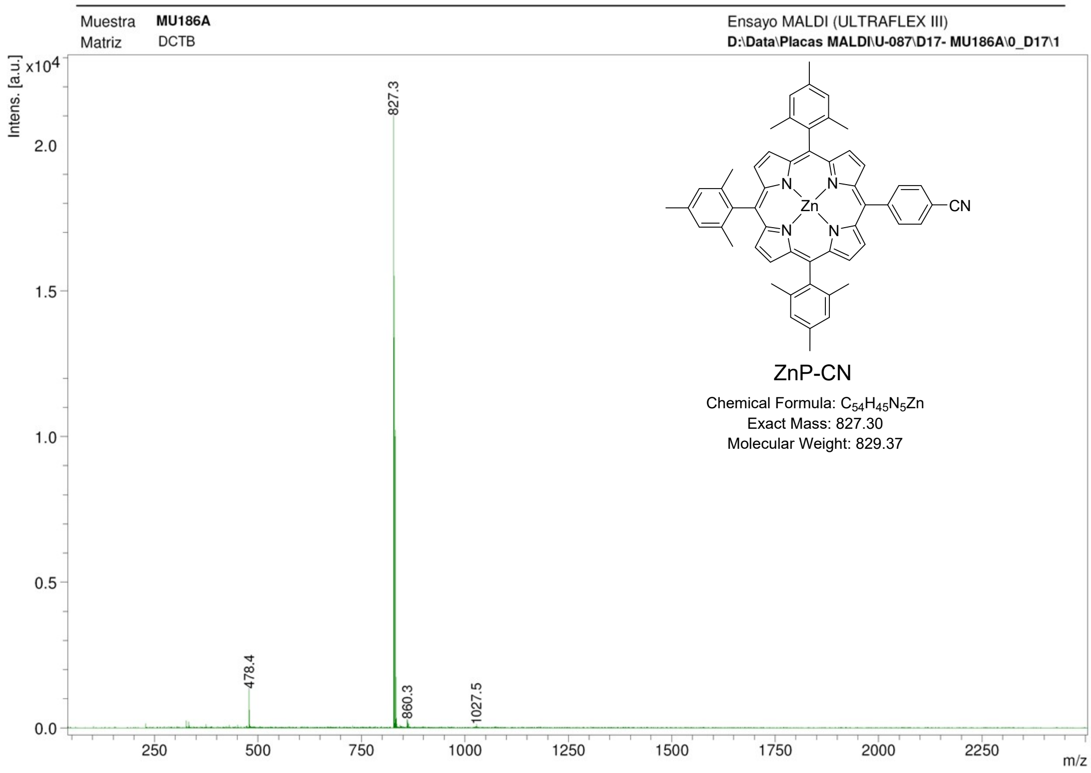

Figure S13. MS spectrum of ZnP-CN (MALDI-TOF$^+$; Matrix: DCTB).

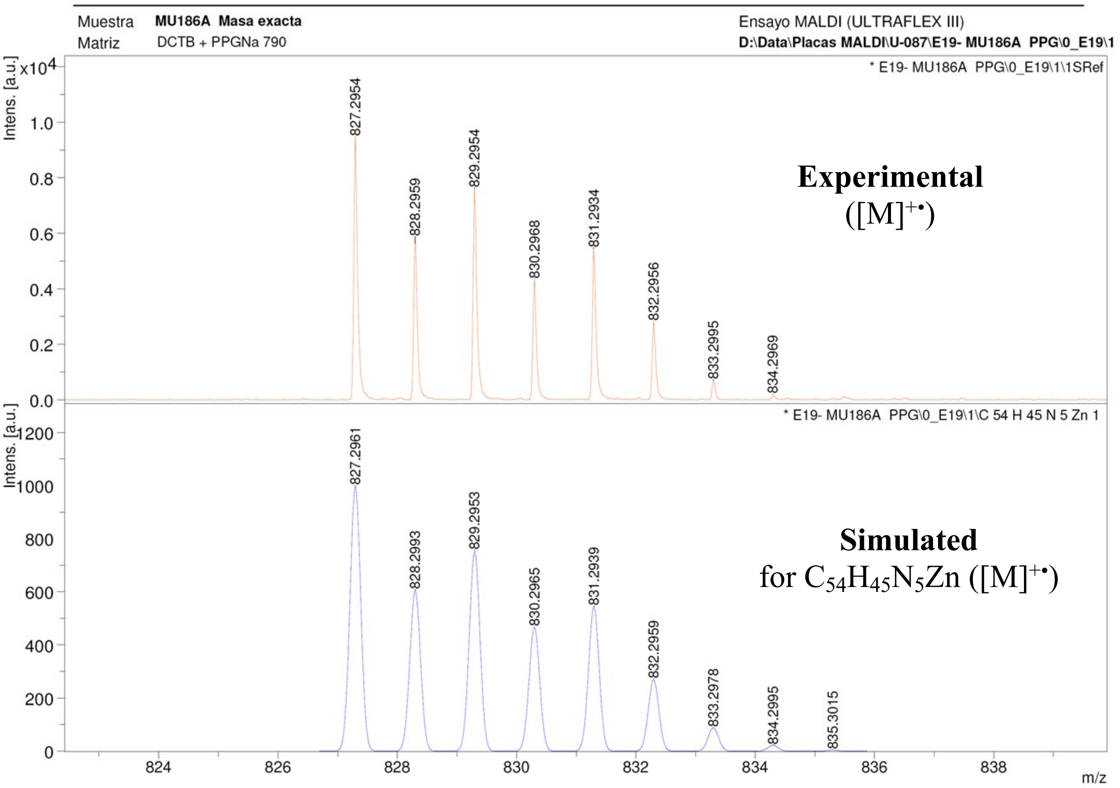

Figure S14. HRMS spectrum of ZnP-CN (top inset; MALDI-TOF$^+$; Matrix: DCTB + PPGNa 790) and isotopic distribution simulated for $[M]^{+\bullet}$ (bottom inset).

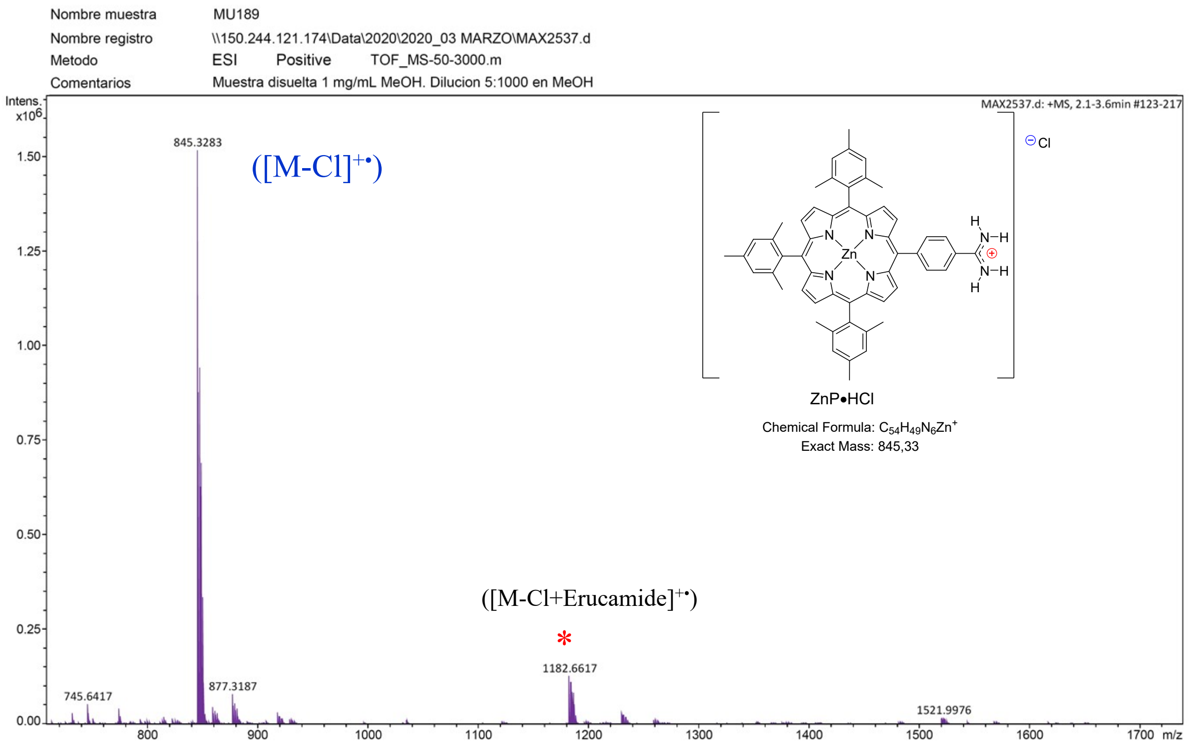

Figure S15. HRMS spectrum of ZnP•HCl (ESI$^+$-TOF; Matrix: MeOH; * denotes the association with Erucamide (+337da), which is a common plastic additive/contaminant from ESI MS experiments).

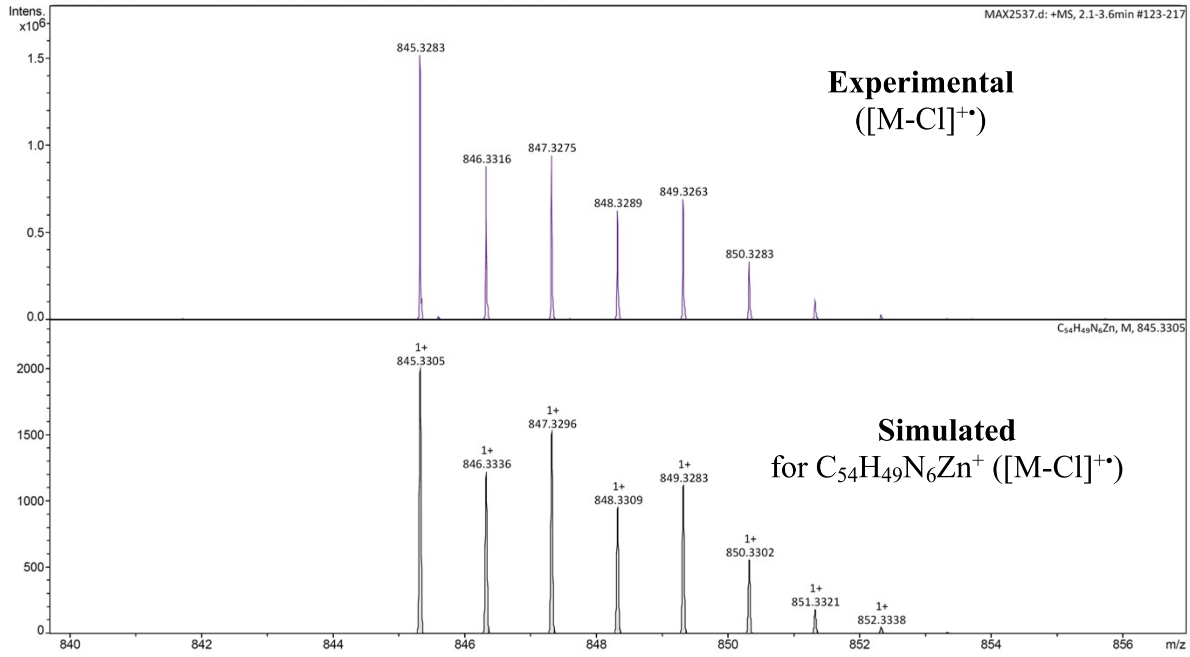

Figure S16. HRMS spectra of ZnP•HCl (top inset; ESI$^+$-TOF; matrix: MeOH) and simulated isotopic distribution for [M-Cl]$^{+\bullet}$ (bottom inset).

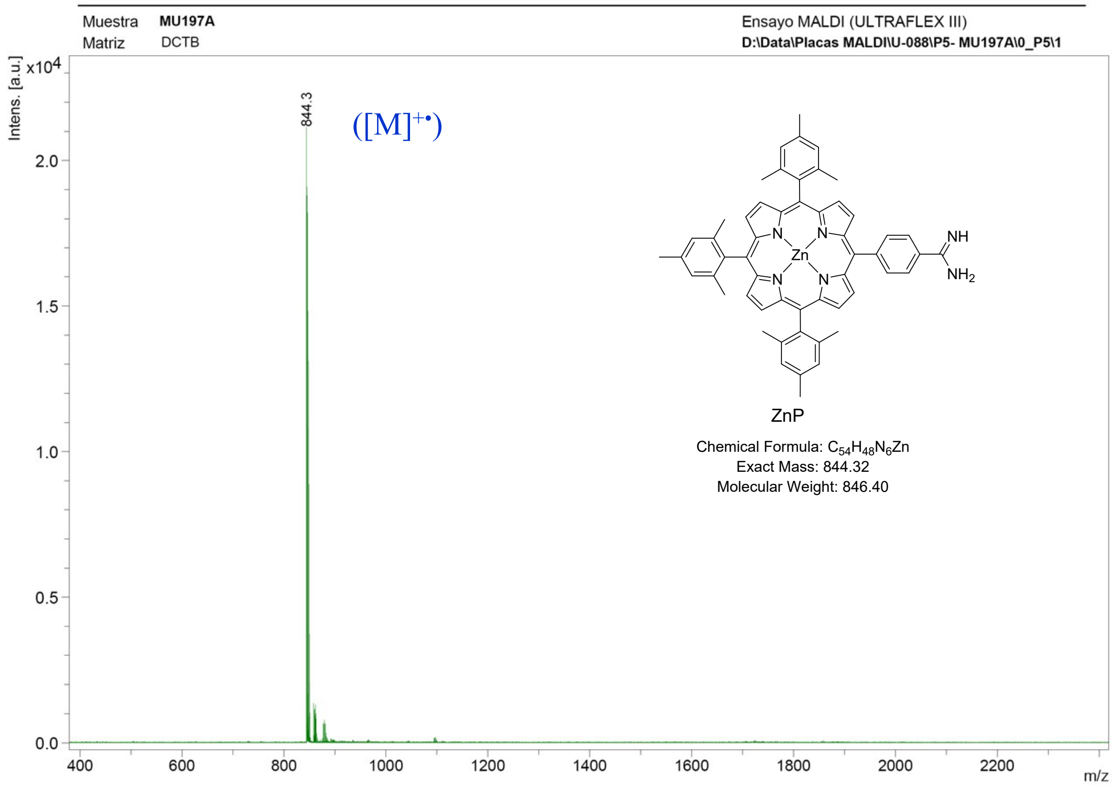

Figure S17. MS spectrum of ZnP (MALDI^{+}-TOF; Matrix: DCTB).

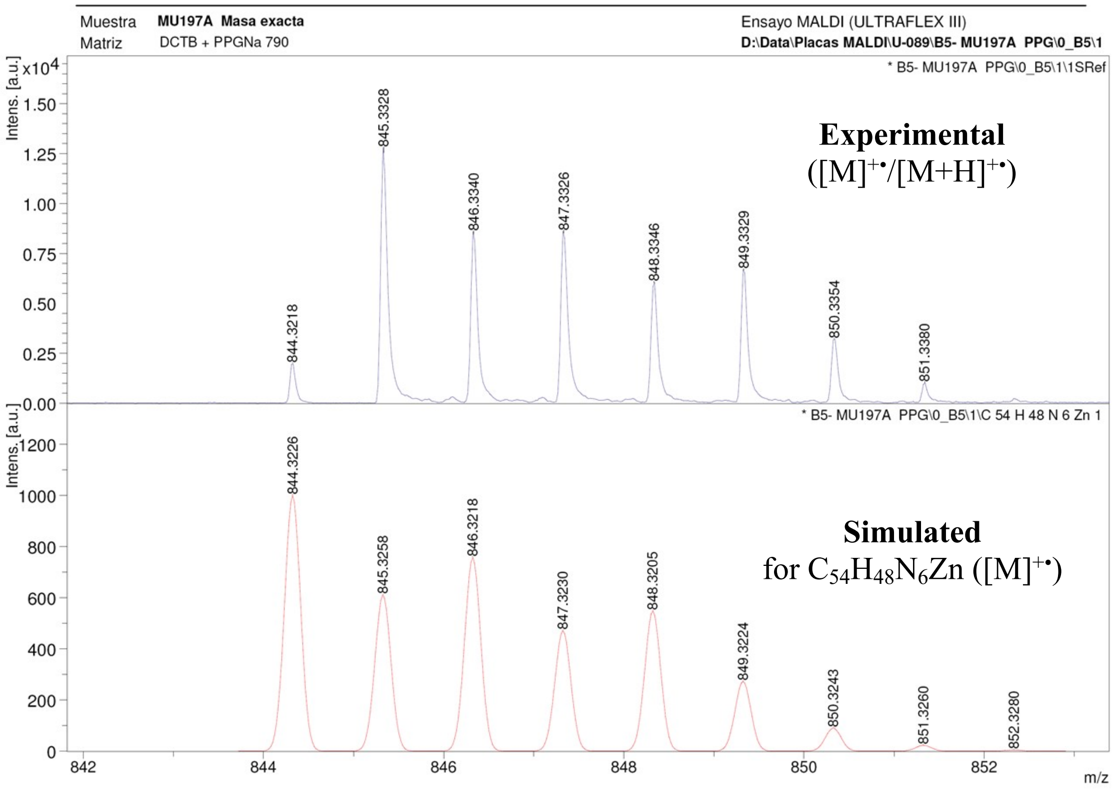

Figure S18. HRMS spectrum of ZnP (top inset; MALDI^{+}-TOF; Matrix: DCTB + PPGNa 790) and isotopic distribution simulated for $[M]^{+\bullet}$ (bottom inset).

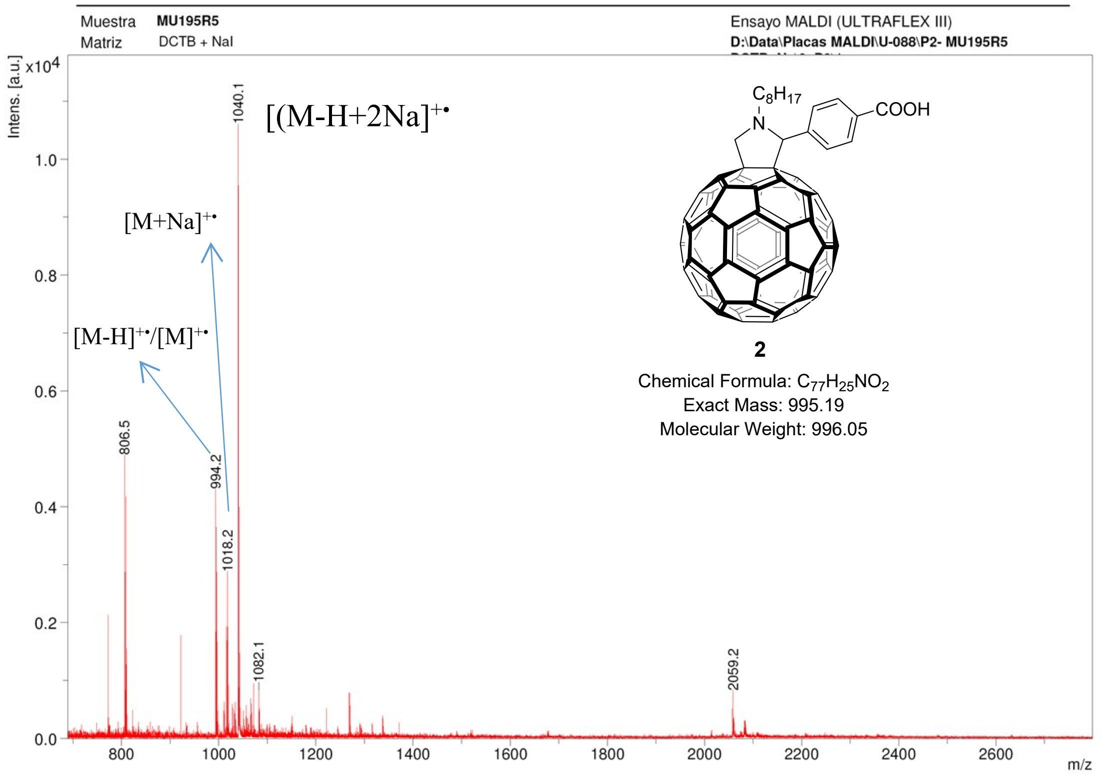

Figure S19. MS spectrum of *N*-Octyl-fulleropyrrolidine-benzoic acid (**2**) (MALDI^{+}-TOF; Matrix: DCTB + NaI).

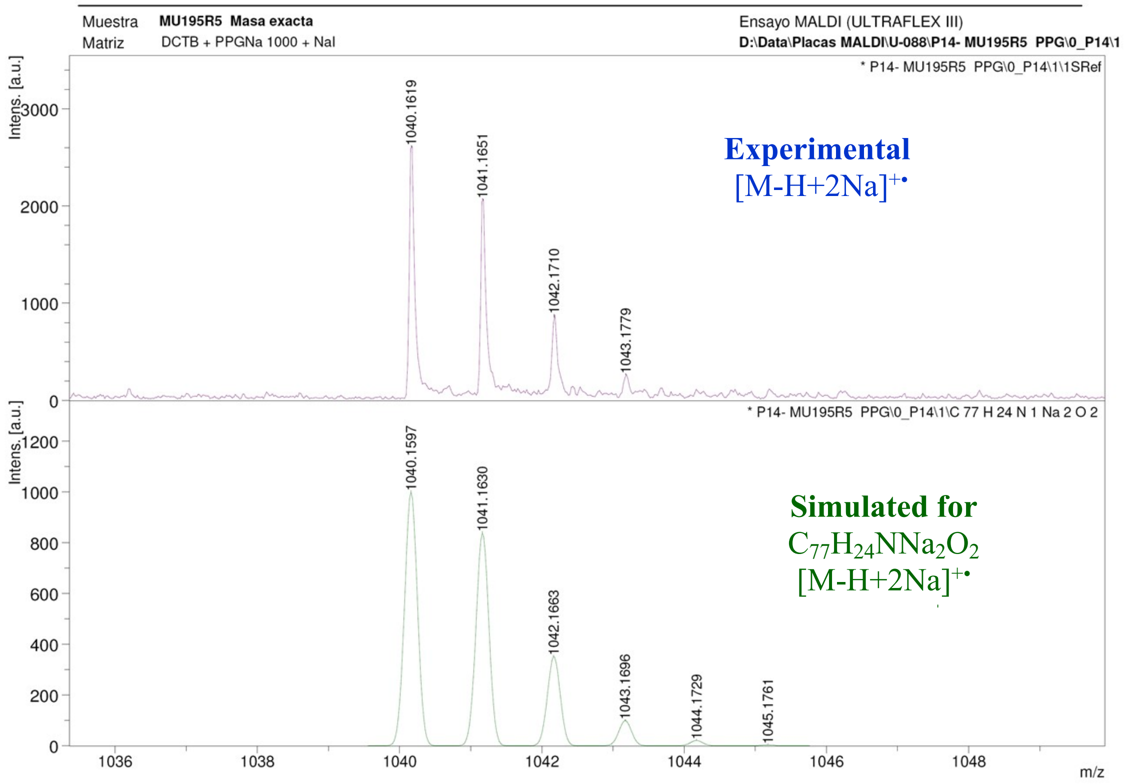

Figure S20. HRMS spectrum of *N*-Octyl-fulleropyrrolidine-benzoic acid (**2**) (top inset; MALDI^{+}-TOF; matrix: DCTB + NaI + PPGNa 1000) and simulated isotopic distribution for $[(M+2Na-H)]^{+\bullet}$ (bottom inset).

5 Atmospheric Pressure Absorption and Emission Spectra

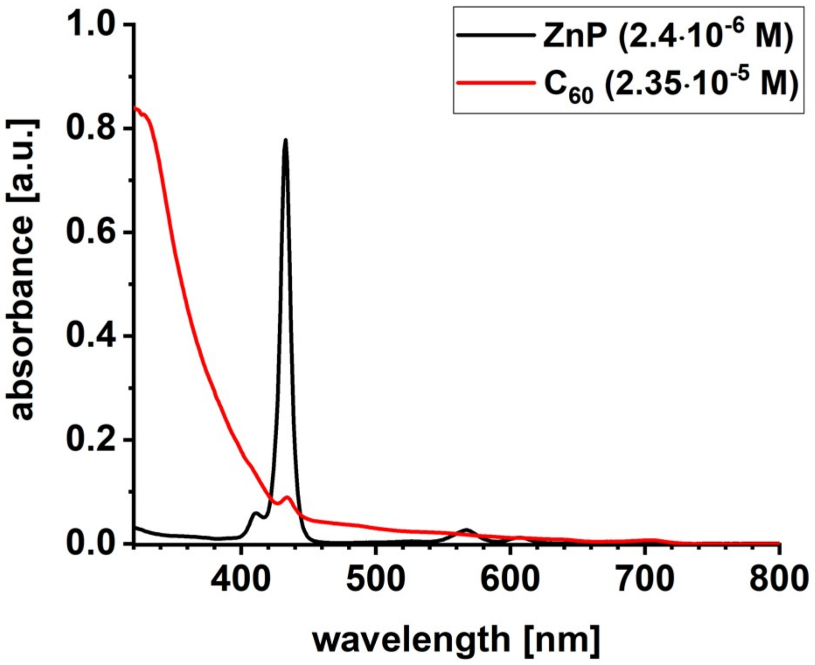

Figure S21. Absorption spectra of ZnP and *N*-Octyl-fulleropyrrolidine-benzoic acid in anisole. A 1000-fold excess of DMAP *vs*. ZnP was used in all optical spectroscopy experiments. The spectra were recorded using the FS5 spectrofluorometer from Edinburgh Instruments with an optical pathlength of 10 mm.

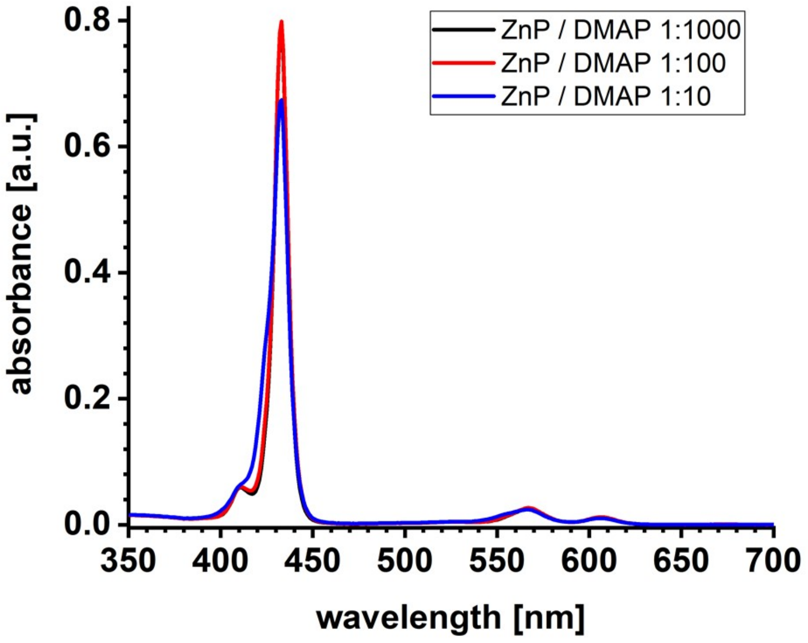

Figure S22. Absorption spectra of a 2.4 x 10^{-6} M solution of ZnP in anisole with various molar ratios of 4-dimethylaminopyridine (DMAP). The apical binding of DMAP to the Zn-center suppresses π-stacking of ZnP, observable through narrowing of the Soret band. The spectra were recorded using the FS5 spectrofluorometer from Edinburgh Instruments with an optical pathlength of 10 mm.

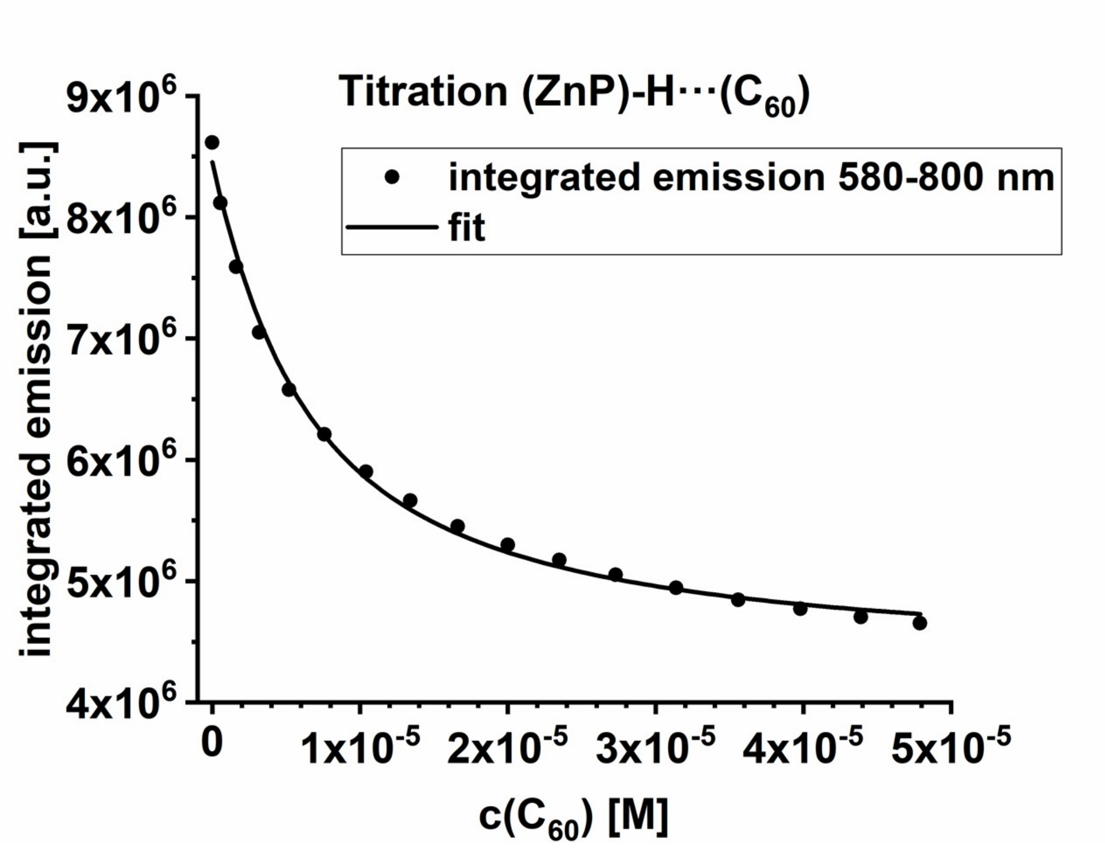

Figure S23. Titration of ZnP with C_{60} for binding constant determination from the fluorescence spectra following 567 nm photoexcitation. A 1000-fold excess of DMAP *vs*. ZnP was used during all optical spectroscopy experiments.

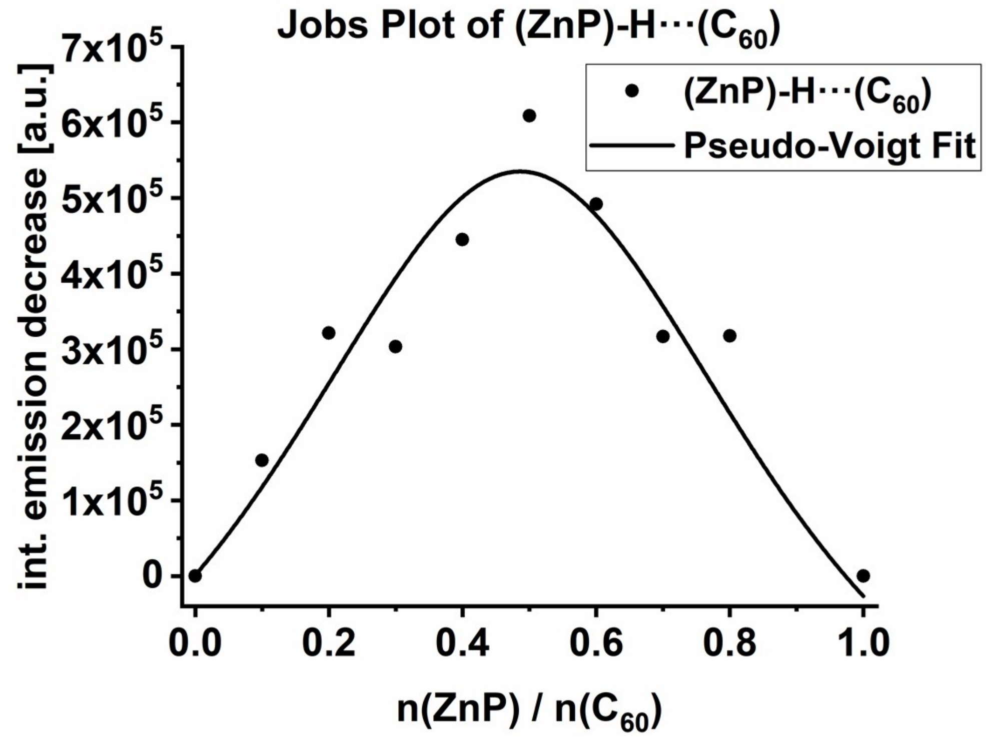

Figure S24. Jobs plot form fluorescence quenching of ZnP and C_{60} recorded with 567 nm photoexcitation, with a total concentration of 2.4 x 10^6 M. A 1000-fold excess of DMAP *vs*. ZnP was used in all optical spectroscopy experiments.

6 Theoretical Calculations

All quantum chemical simulations were performed using Gaussian 16. Density functional theory (DFT) using the CAM-B3LYP functional[7] and the def2-SVP basis set[8] was used to optimize the geometry of the free monomers, homodimers, and heterodimers. Solvent effects were included by means of the polarizable continuum model (PCM) for anisole[9]. Since only differences related to trends were interpreted from the calculated values, optimizations at the CAM-B3LYP/def2-SVP level were considered sufficiently accurate. To correct the results for the basis set superposition error, counterpoise calculations[10] were performed at the CAM-B3LYP/def2-SVP and CAM-B3LYP/def2-TZVP[7] levels.

The excited-state properties, namely vertical transition energies (VEEs), oscillator strengths (f), and the character of electronic transitions for ZnP(DMAP) monomer and the heterodimers ZnP(DMAP)-H···C_{60} and ZnP(DMAP)···H-C_{60}, were calculated at time-dependent density functional theory (TD-DFT) level of theory. For these computations, DMAP was coordinated to the ZnP for better agreement with the experimental results. Here, the CAM-B3LYP/def2-TZVP//CAM-B3LYP/def2-SVP basis set and functional were used. The lowest 50 singlet-singlet or 100 triplet-triplet excitations were calculated within the respective multiplicity of the electronic ground state (S_0 and T_1 state of ZnP(DMAP)-H···C_{60} and only S_0 otherwise). The non-equilibrium solvation model was employed in the computation of the excited-state properties. All calculations were performed including Grimme D3 dispersion correction with Becke-Johnson damping (GD3BJ).

a) b)

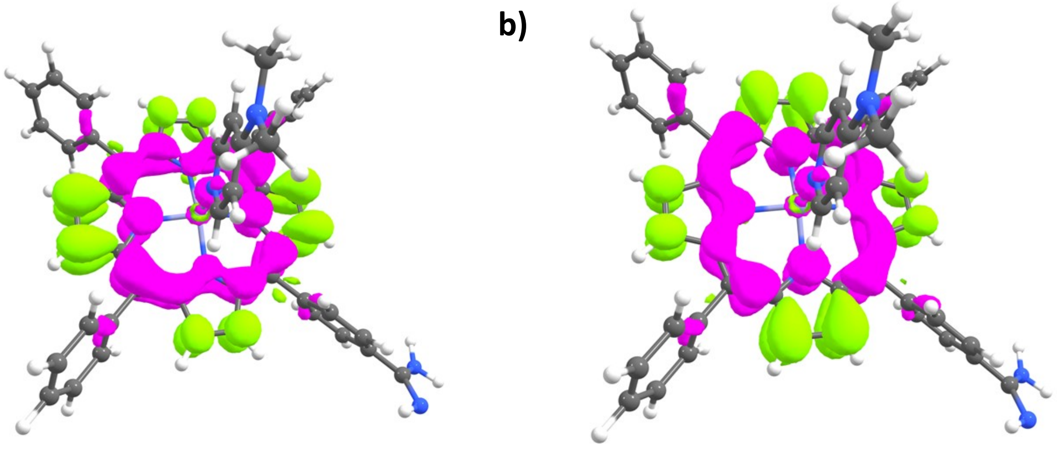

S_1
574 nm (2.16 eV)
f = 0.012

S_2
570 nm (2.17 eV)
f = 0.014

Figure S25. Charge density difference (CDD), transition energies (eV and nm) and oscillator strength of the vertical excitation into the a) S_1 and b) S_2 of ZnP(DMAP) comprising the Q-band of the absorption spectrum, with increasing (green) und decreasing (magenta) excited state density at a Isovalue of 0.0003.

a) b)

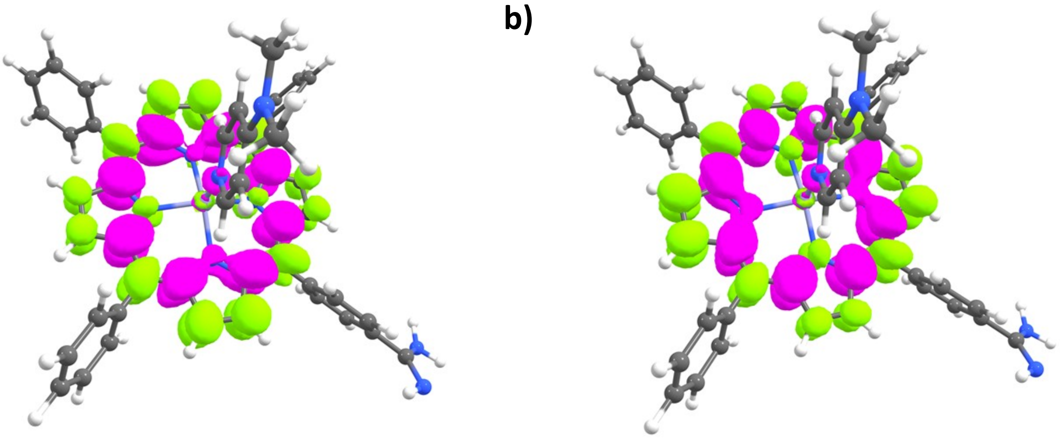

S_3
399 nm (3.11 eV)
f = 1.620

S_4
398 nm (3.12 eV)
f = 1.649

Figure S26. Charge density difference (CDD), transition energies (eV and nm) and oscillator strength of the vertical excitation into the a) S_3 and b) S_4 of ZnP(DMAP) comprising the B-band of the absorption spectrum, with increasing (green) und decreasing (magenta) excited state density at a Isovalue of 0.0003.

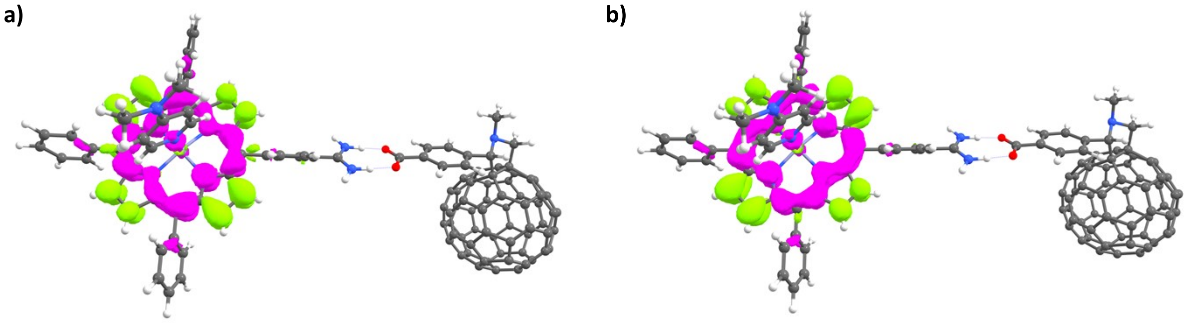

S_1
574 nm (2.16 eV)
f = 0.013

S_2
570 nm (2.17 eV)
f = 0.013

Figure S27. Charge density difference (CDD), transition energies (eV and nm) and oscillator strength of the vertical excitation into the a) S_1 and b) S_2 of ZnP(DMAP)-H···C_{60} comprising the Q-band of the absorption spectrum, with increasing (green) und decreasing (magenta) excited state density at a Isovalue of 0.0003.

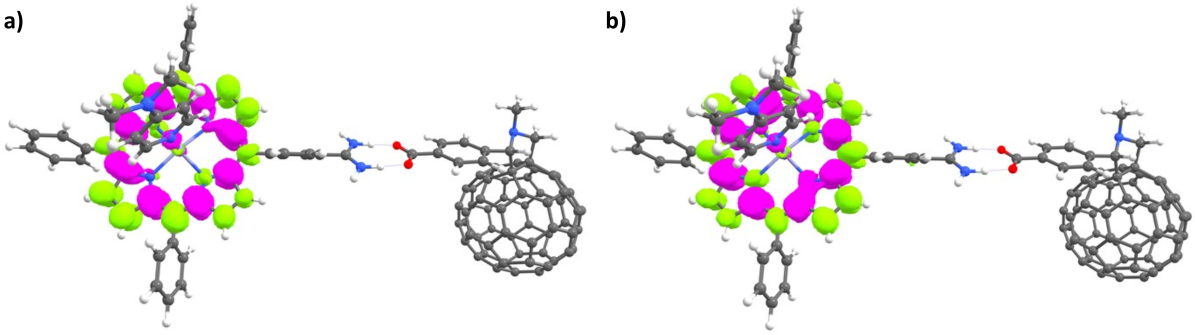

S_{15}
399 nm (3.11 eV)
f = 1.702

S_{16}
398 nm (3.12 eV)
f = 1.647

Figure S28. Charge density difference (CDD), transition energies (eV and nm) and oscillator strength of the vertical excitation into the a) S_{15} and b) S_{16} of ZnP(DMAP)-H···C_{60} comprising the B-band of the absorption spectrum, with increasing (green) und decreasing (magenta) excited state density at a Isovalue of 0.0003.

To compute the formation enthalpies, we first optimized the ground-state equilibrium geometries of the monomers ZnP and C_{60}, their respective homodimers, namely ZnP···ZnP and C_{60}···C_{60} (see Figure S29), and their heterodimers, namely ZnP-H···C_{60} and ZnP···H-C_{60} (*cf.* Figure S30). Based on the sum of the energy of two monomers or the energy of the homodimers with respect to the homo- and heterodimers, we can compute the reaction enthalpies of the dimer formation from the monomers (*cf.* Table S1) and the heterodimer formation from the homdimers (*cf.* Table S2).

The reaction enthalpies of the heterodimer formation from the free monomers show that the configuration with both bridging protons on the porphyrin side (ZnP-H···C_{60}) is the most favorable configuration among the studied dimers (*cf.* Table S1). The formation of the heterodimer with one proton on both, the ZnP and C_{60} (ZnP···H-C_{60}) side is enthalpically less favorable. The same accounts for a formation of the respective homodimers C_{60}···C_{60} and ZnP···ZnP. Similarly, the reaction enthalpy for the formation of the heterodimer from the homodimers show again, that ZnP-H···C_{60} with both protons on the porphyrin side are enthalpically the most favorable product (*cf.* Table S2).

The reaction enthalpies of the proton transfer reaction across the amidinium-carboxylate bridge were calculated. For this purpose, one of the bridging protons was relocated from the equilibrated configuration towards the transition state, where it is located in an equidistant position between the amidine-NH group and the carboxylic acid-CO group. This process was found to be enthalpically endothermic for all homodimers and all heterodimers, where both bridging protons were initially located at the porphyrin side. On the other hand, it was found to be exothermic when the proton movement in the heterodimers started at the C_{60} side from the ZnP···H-C_{60} configuration (*cf.* Table S3). From these results it can be deduced that ZnP in solution with C_{60} appear as shown in Figure 1 (main manuscript).

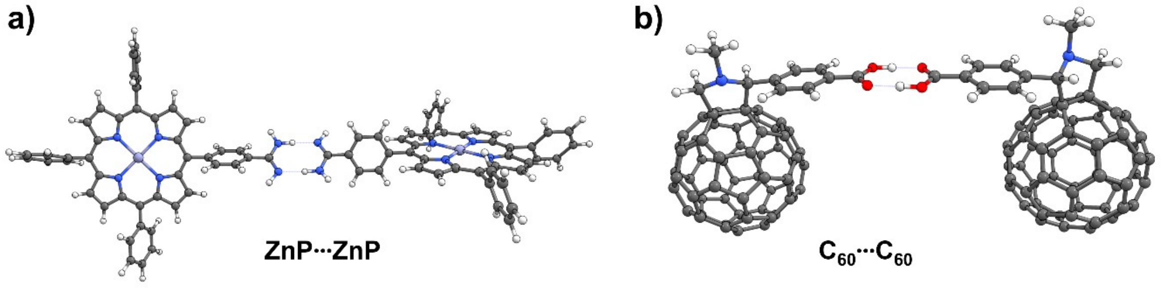

Figure S29. Depiction of the molecular conformation, used for calculating the energies for the homodimerization. a) zinc porphyrin-zinc porphyrin (ZnP···ZnP) dimer, b) fullerene-fullerene (C_{60}···C_{60}) dimer.

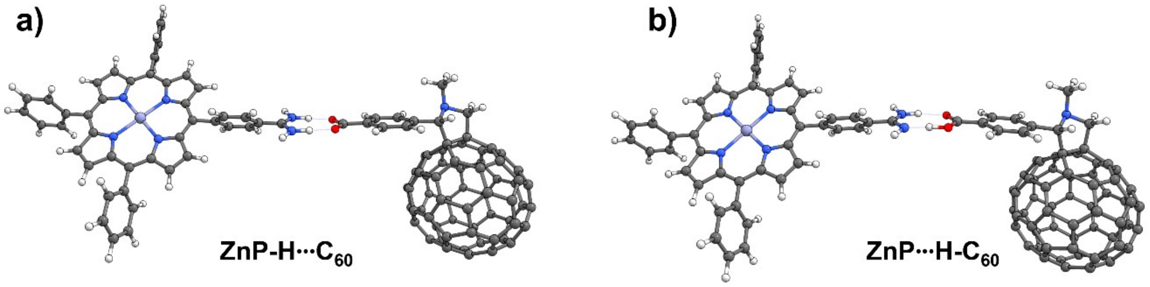

Figure S30. Depiction of the molecular conformation, used for calculating the energies for the heterodimerization. a) ZnP-H···C_{60} with both bridging H-atoms located at the amidine side, forming a charged amidinium-carboxylate bridge. b) ZnP···H-C_{60} with the bridging H-atoms on either side, forming a charge-neutral amidine-carboxylic acid bridge.

Table S1. Enthalpy of formation of hetero- and homodimers from the free monomers at CP CAM-B3LYP/def2-TZVP//CAM-B3LYP/def2-SVP level.

Complex	ΔE [kcal/mol]
C_{60}···C_{60}	-25.83
ZnP···ZnP	-18.47
ZnP-H···C_{60}	-117.91
ZnP···H-C_{60}	-31.36

Table S2. Enthalpy of formation of heterodimer from the homodimers at CAM-B3LYP/def2-SVP level.

Complex	ΔE [kcal/mol]
ZnP-H···C_{60}	-14.71
ZnP···H-C_{60}	-10.41

Table S3. Enthalpy of formation of the transition state for the proton transfer across the bridging unit from hetero and homo dimers at CAM-B3LYP/def2-SVP level. All molecular subunits are in their respective electronic ground state. The transition state was defined by repositioning one of the bridging H-atoms from the equilibrated configuration (*vide supra*) to the equidistant position along the bridge. The position of the H-label in the abbreviated nomenclature represents the starting position of the H-atom, from where it was moved to the equidistant position.

Start Configuration	ΔE [kcal/mol]
C_{60}···C_{60}	0.15
ZnP···ZnP	6.90
ZnP-H···C_{60}	0.51
ZnP···H-C_{60}	-1.63

7 Pressure Dependent Steady State Experiments

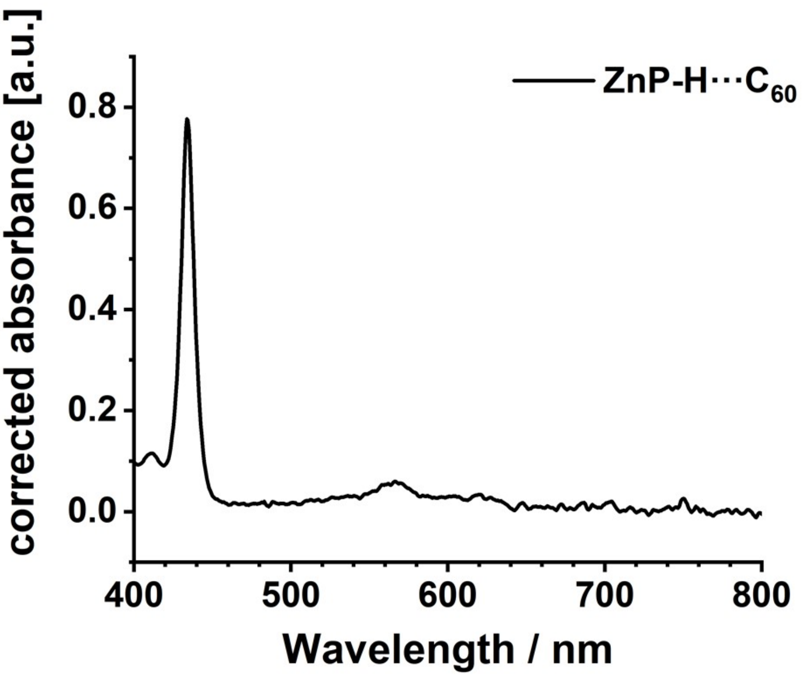

Figure S31. **A**bsorption spectrum of ZnP-H···C_{60} recorded at 120 MPa in anisole. The absorption spectrum of C_{60} under identical condition was subtracted to pinpoint the ZnP-centered absorption change induced by pressure, as depicted in Figure 4 in the main manuscript. A 1000-fold excess of DMAP vs. ZnP was used in all optical spectroscopy experiments.

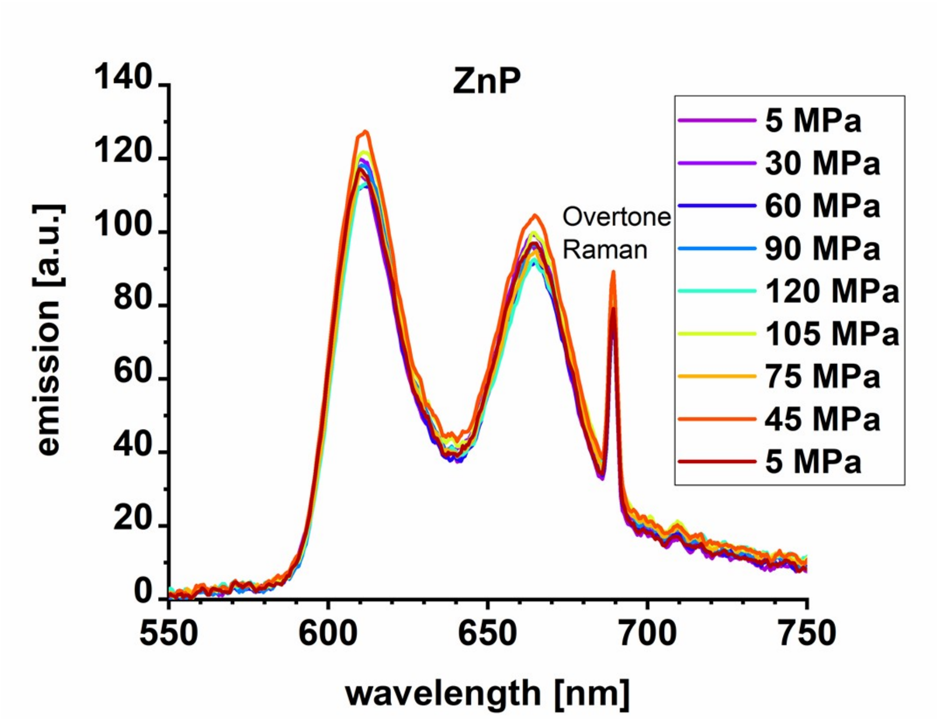

Figure S32. Unmodified pressure dependent fluorescence spectra of ZnP following Soret band photoexcitation at 430 nm. The linear emission detection setup of the pressure dependent emission experiments allowed for Soret band photoexcitation while avoiding primary inner filter effect. See method section in the main manuscript for more details. The integrated range of 550-682 nm was used to calculate the quenching factor as described in the main manuscript. A 1000-fold excess of DMAP vs. ZnP was used in all optical spectroscopy experiments.

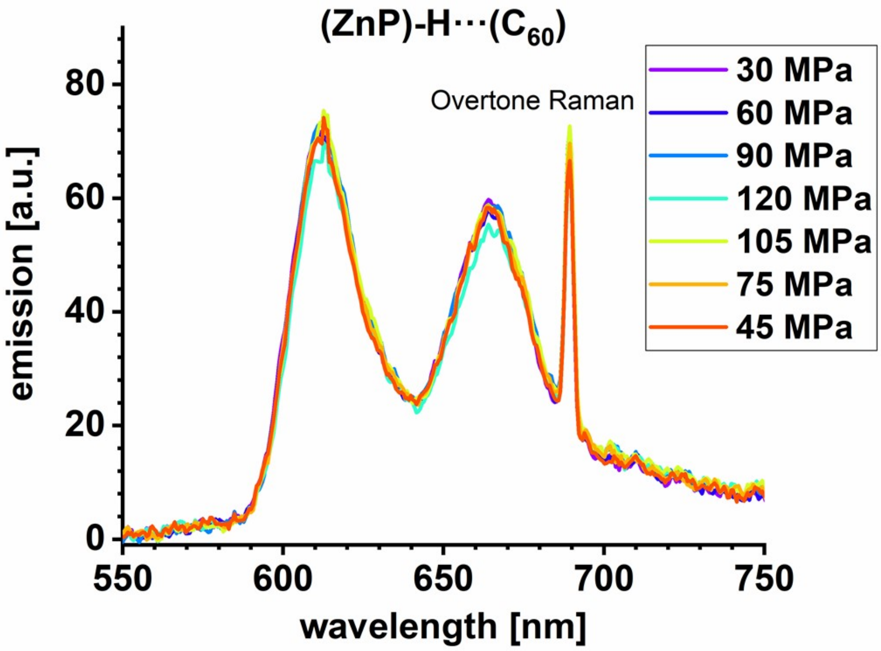

Figure S33. Unmodified pressure dependent fluorescence spectra of ZnP-H···C_{60} after Soret band photoexcitation at 430 nm. The linear emission detection setup of the pressure dependent emission experiments allowed for Soret band photoexcitation while avoiding primary inner filter effect. See methods section in main manuscript for more details. The integrated range of 550-682 nm was used to calculate the quenching factor as described in the main manuscript. A 1000-fold excess of DMAP vs. ZnP was used in all optical spectroscopy experiments.

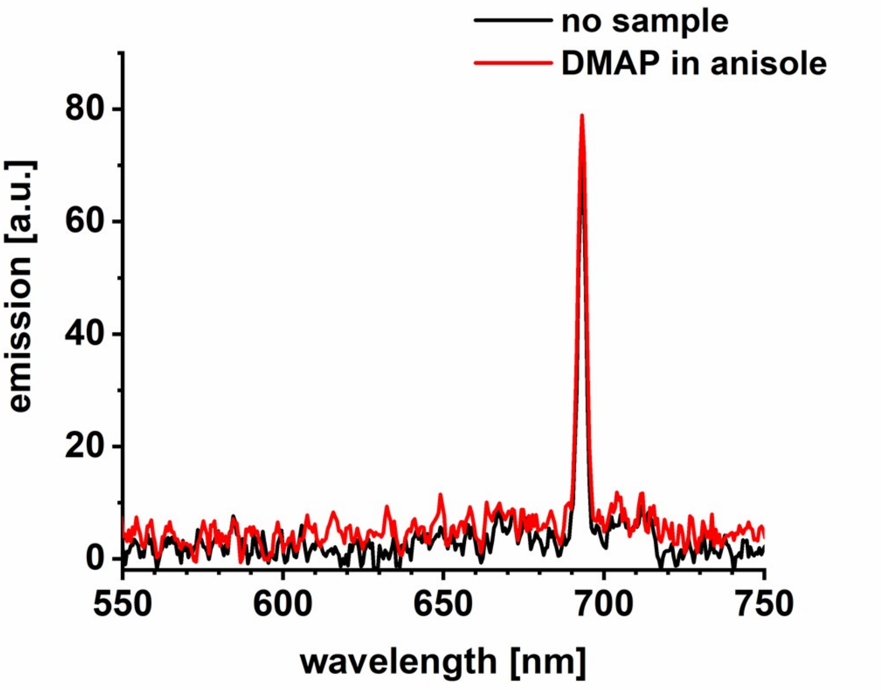

Figure S34. Emission spectrum recorded with the pressure apparatus using photoexcitation at 430 nm. The black spectrum was recorded using the pressure cell without a cuvette placed in the sample chamber, containing only water as the pressure medium at 5 MPa. The red spectrum was recorded with a cuvette placed in the pressure medium at 5 MPa, containing DMAP dissolved in anisole in identical concentration as used for all other experiments. The appearance of the overtone Raman band is distinguishable at the identical position, as observed for the emission experiments with ZnP and ZnP-H···C_{60}. Consequently, this band can be excluded for evaluating the pressure effect of the emission data, as it originates as an artefact from the experimental setup.

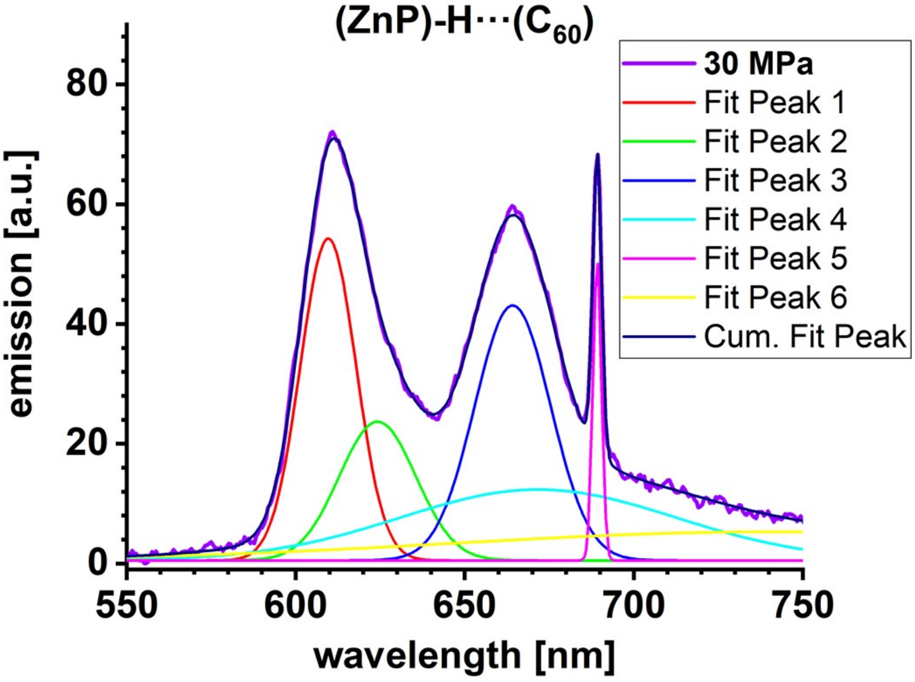

Figure S35. Emission spectrum of ZnP-H···C_{60} at 30 MPa after Soret band photoexcitation at 430 nm and the results obtained after gaussian fitting of the emission spectrum.

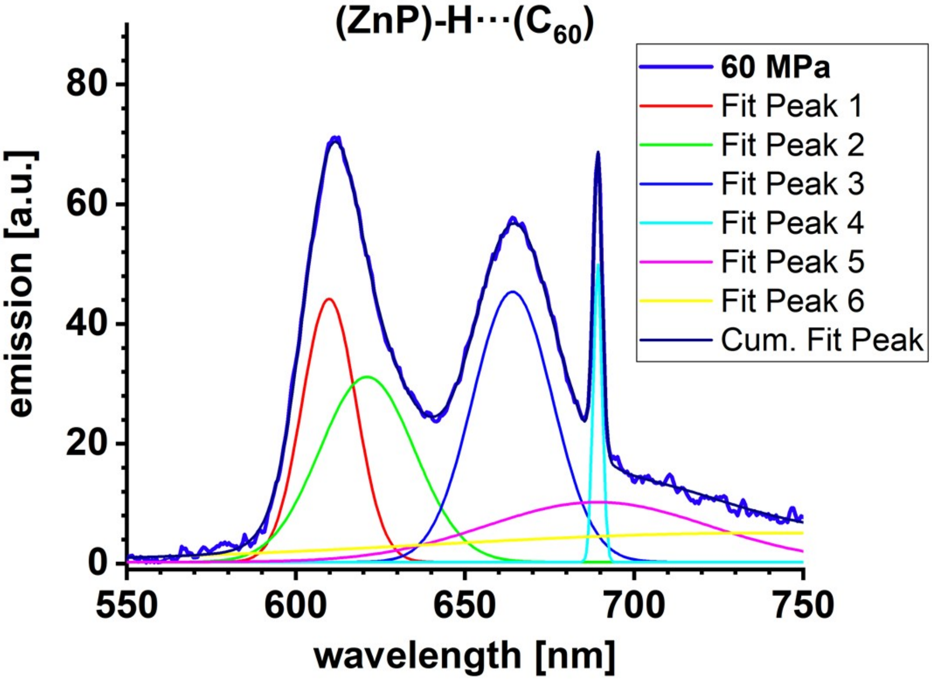

Figure S36. Emission spectrum of ZnP-H···C_{60} at 60 MPa after Soret band photoexcitation at 430 nm and the results obtained after gaussian fitting of the emission spectrum.

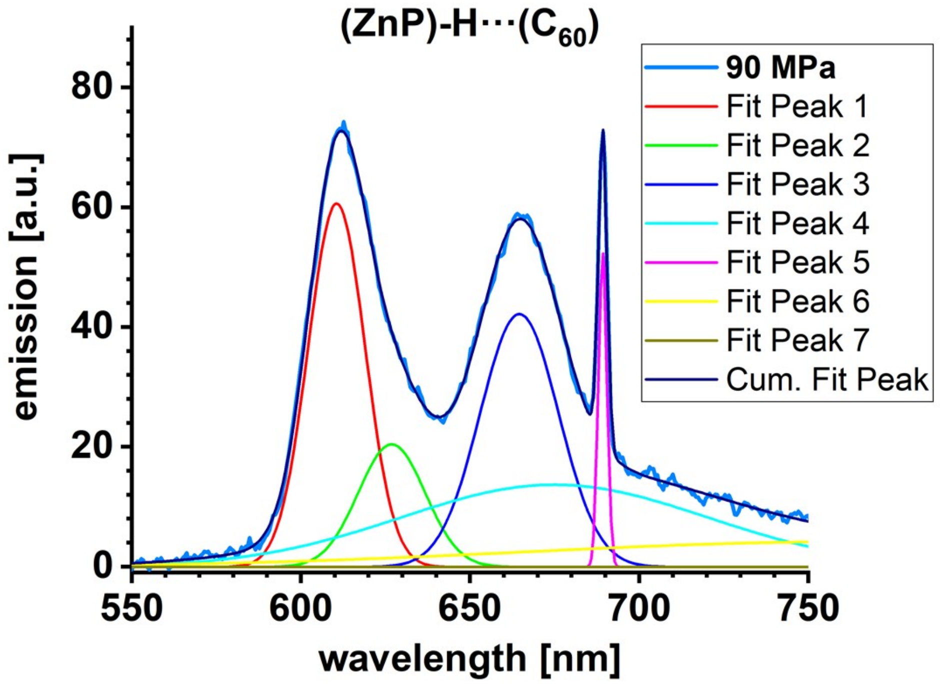

Figure S37. Emission spectrum of ZnP-H···C_{60} at 90 MPa after Soret band photoexcitation at 430 nm and the results obtained after gaussian fitting of the emission spectrum.

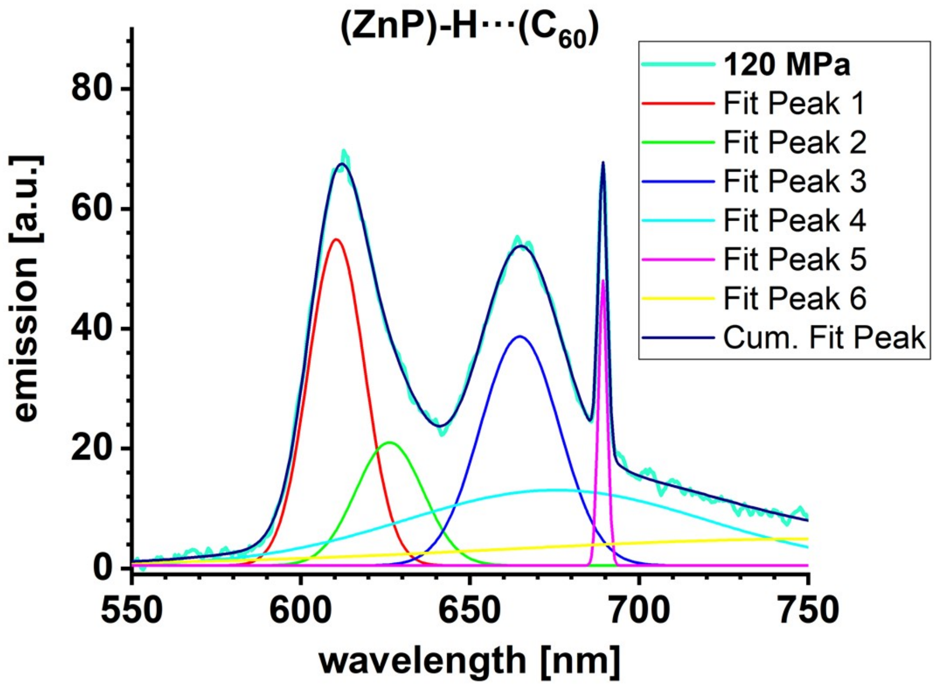

Figure S38. Emission spectrum of ZnP-H···C_{60} at 120 MPa after Soret band photoexcitation at 430 nm and the results obtained after gaussian fitting of the emission spectrum.

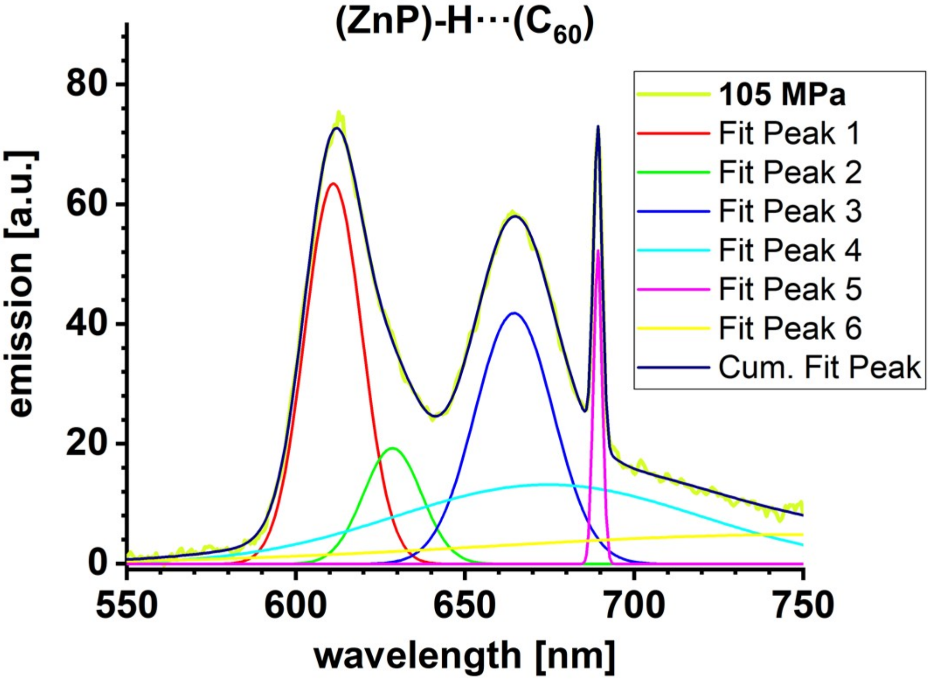

Figure S39. Emission spectrum of ZnP-H···C_{60} at 105 MPa after Soret band photoexcitation at 430 nm and the results obtained after gaussian fitting of the emission spectrum.

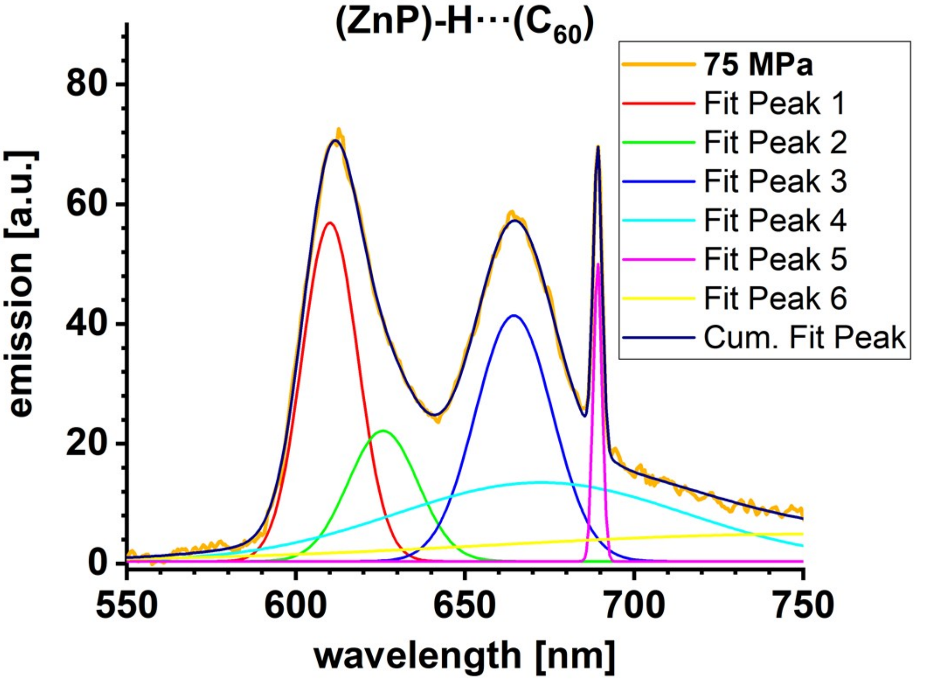

Figure S40. Emission spectrum of ZnP-H···C_{60} at 75 MPa after Soret band photoexcitation at 430 nm and the results obtained after gaussian fitting of the emission spectrum.

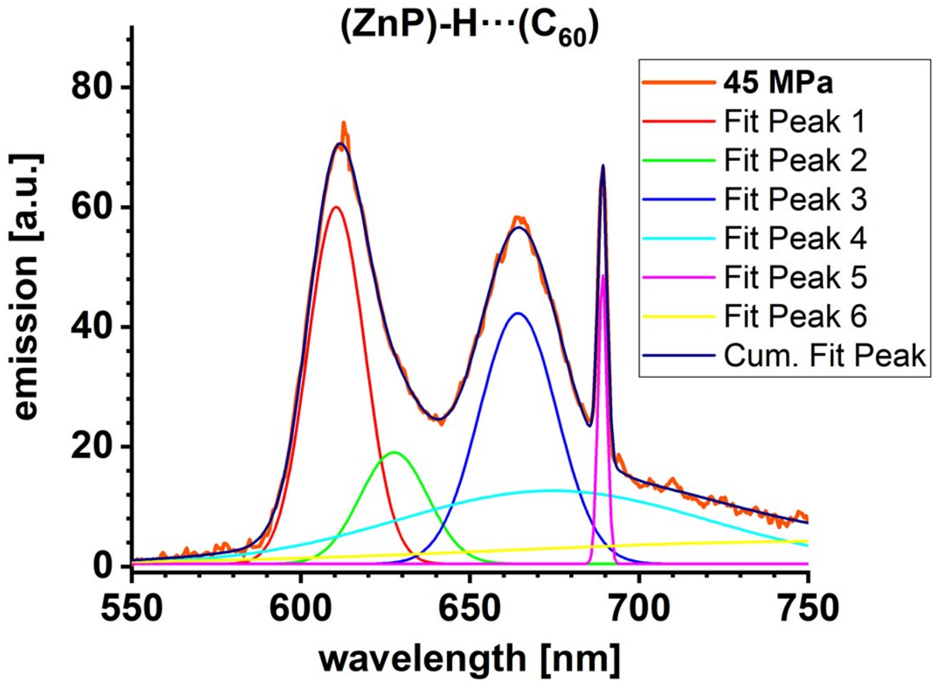

Figure S41. Emission spectrum of ZnP-H···C_{60} at 45 MPa after Soret band photoexcitation at 430 nm and the results obtained after gaussian fitting of the emission spectrum.

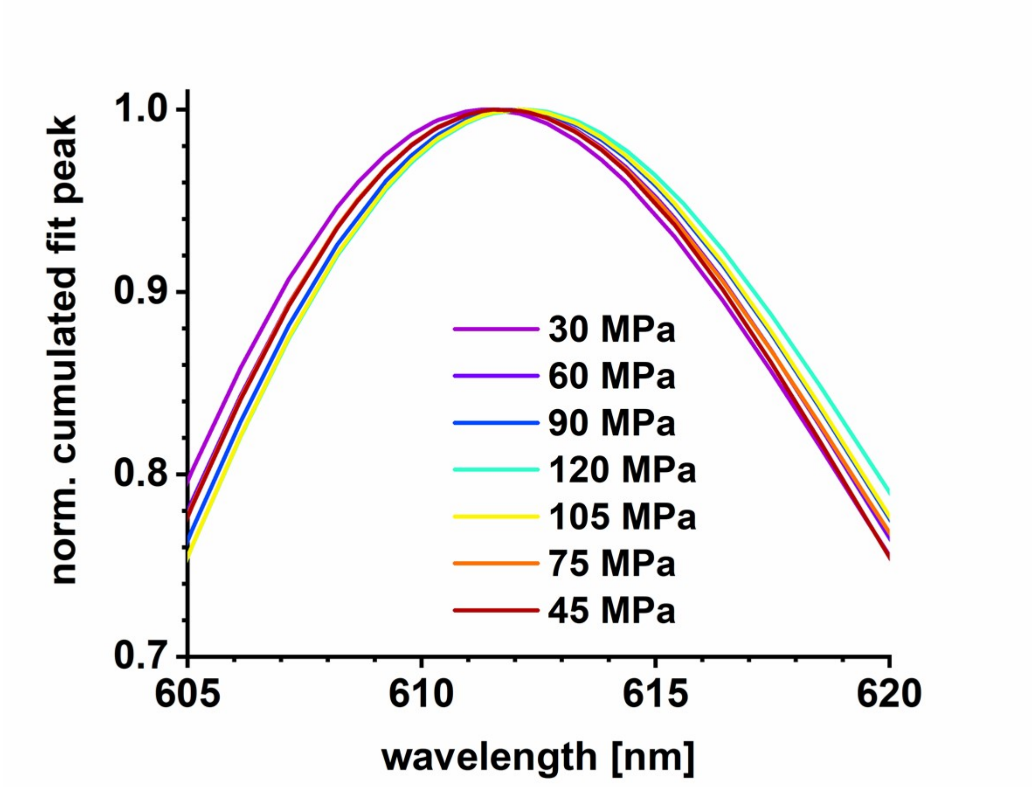

Figure S42. Normalization of the cumulated fit peaks from gaussian fitting of the pressure-dependent emission experiments.

8 Pressure Dependent Transient Absorption Spectroscopy

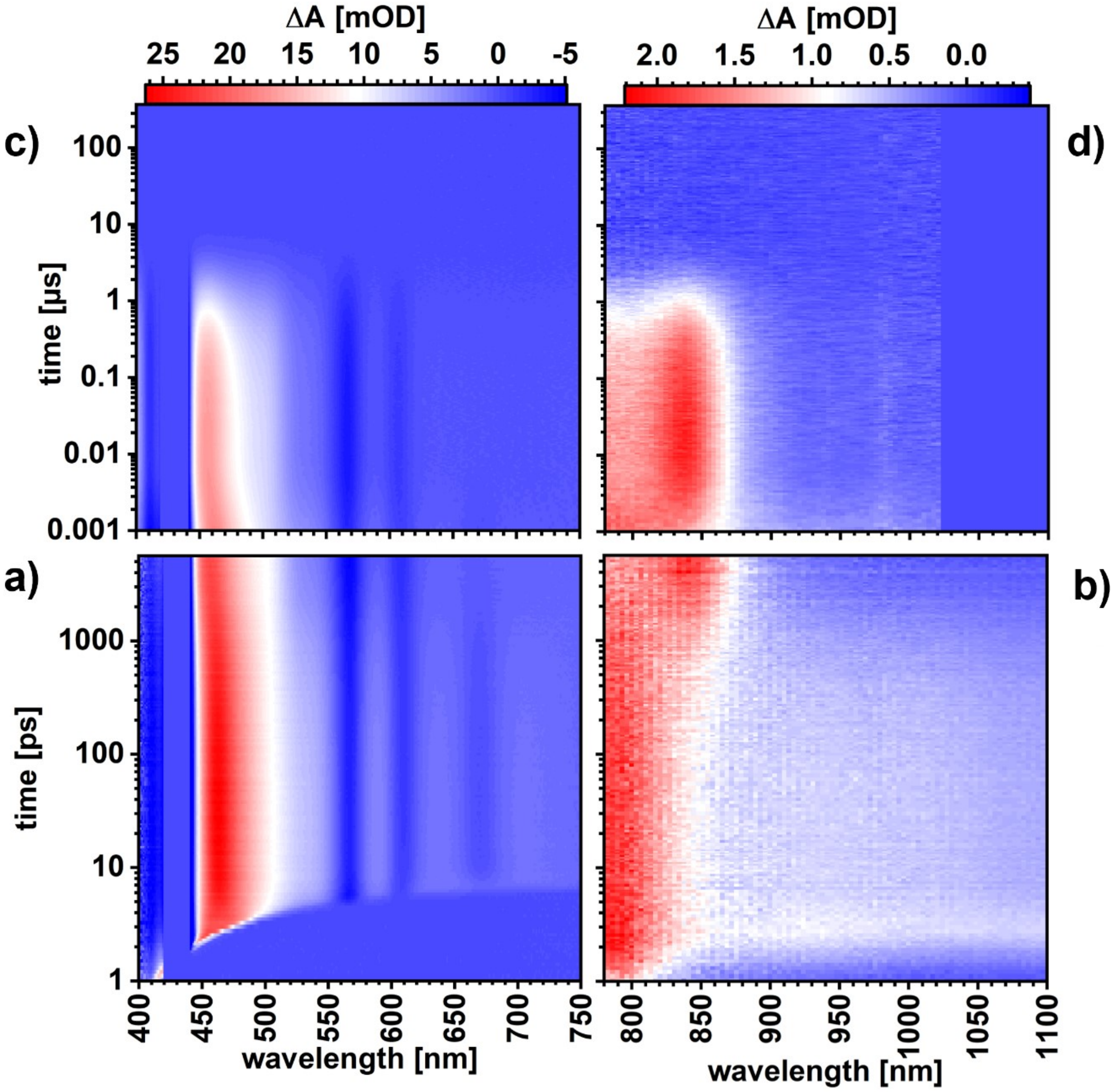

Figure S43. Femtosecond transient absorption spectra in the visible (a) and NIR region (b) of ZnP, after photoexcitation at 430 nm with 200 nJ pulse energy. Nanosecond transient absorption spectra in the visible (c) and NIR (d) under identical condition. Depicted spectra were recorded in anisole at 293 K and 5 MPa.

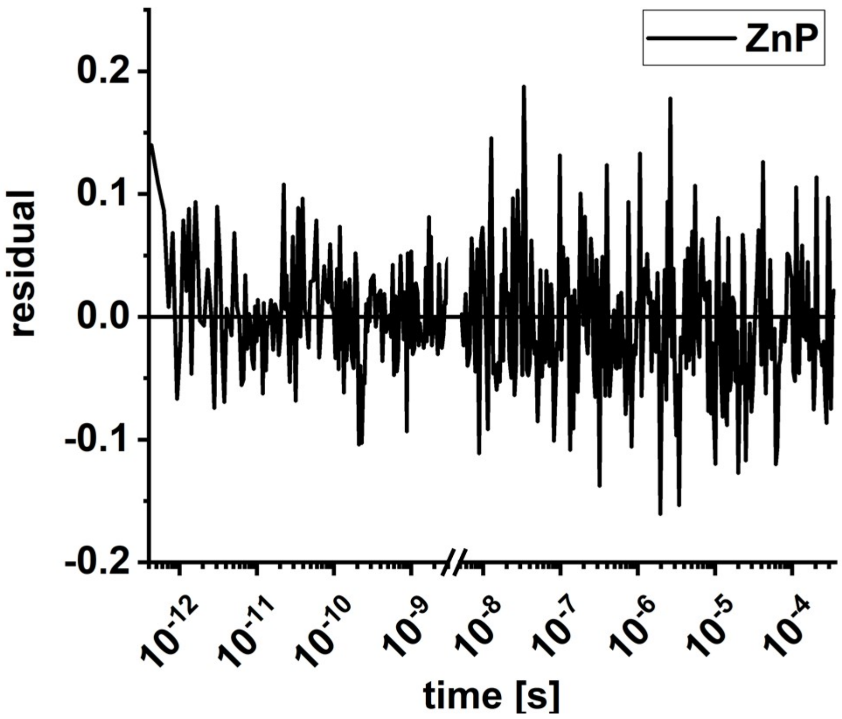

Figure S44. Residual trace after fitting the visible region of the fs- and ns-TA spectra of ZnP at 5 MPa (Figure S8.1a and c). The sequential global analysis model depicted in Figure 5 in the main manuscript was used. A common set of kinetic parameters was applied to model both, the visible and NIR region of the respective fs- and ns-TA spectra. Statistical noise without any observable residual kinetic trace represents a good quality of fit.

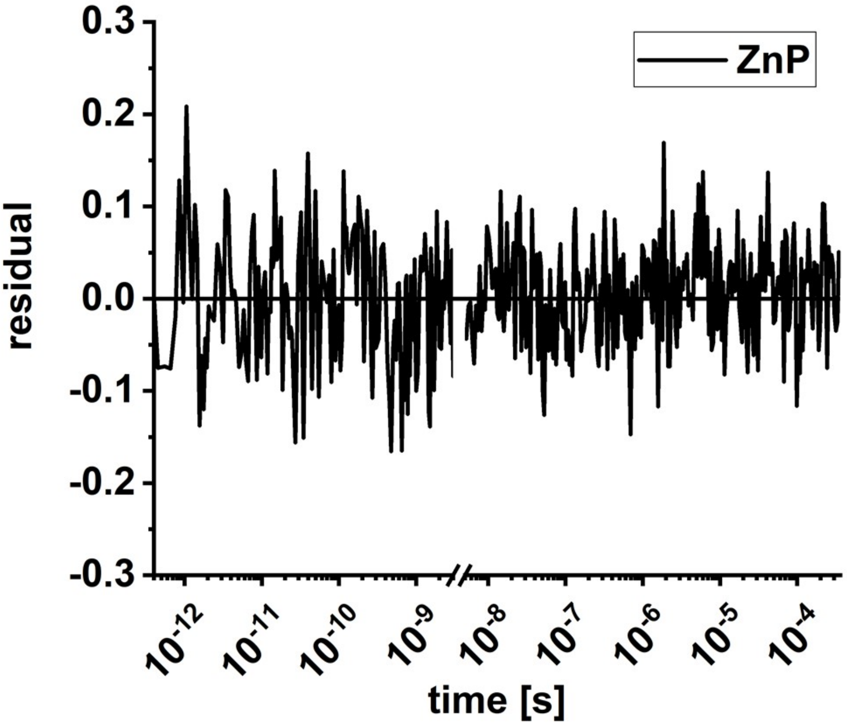

Figure S45. Residual trace after fitting the NIR region of the fs- and ns-TA spectra of ZnP at 5 MPa (Figure S8.1b and d). The sequential global analysis model depicted in Figure 5 in the main manuscript was used. A common set of kinetic parameters was applied to model both the visible and NIR region of the respective fs- and ns-TA spectra. Statistical noise without any observable residual kinetic trace represents a good quality of fit.

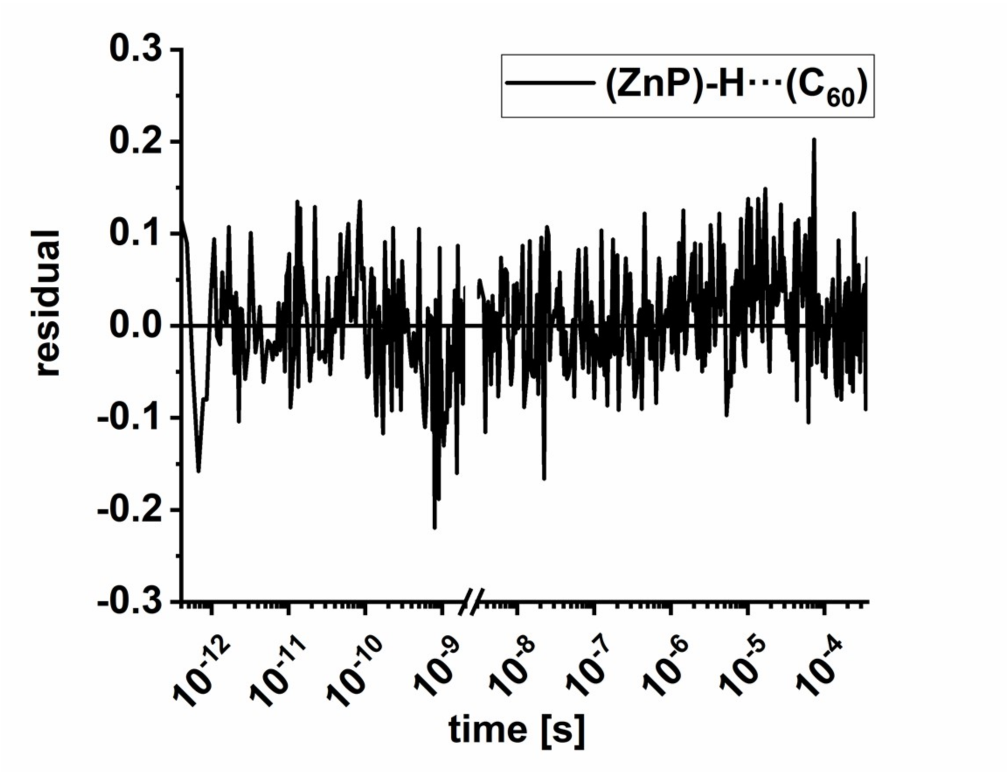

Figure S46. Residual trace after fitting the visible region of the fs- and ns-TA spectra of ZnP-H···C_{60} at 5 MPa (Figure 4a and c in the main manuscript). The branched target analysis model depicted in Figure 5 in the main manuscript was used. A common set of kinetic parameters was applied to model both the visible and NIR region of the respective fs- and ns-TA spectra. Statistical noise without any observable residual kinetic trace represents a good quality of fit.

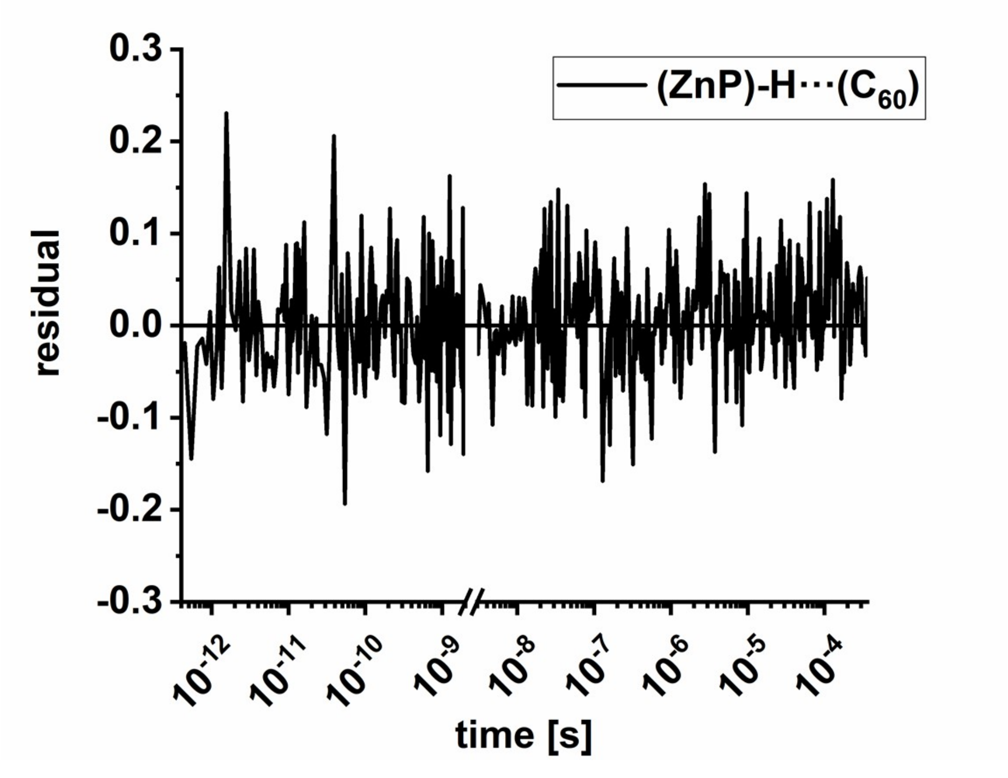

Figure S47. Residual trace after fitting the NIR region of the fs- and ns-TA spectra of ZnP-H···C_{60} at 5 MPa (Figure 4b and d in the main manuscript). The branched target analysis model depicted in Figure 5 in the main manuscript was used. A common set of kinetic parameters was applied to model both the visible and NIR region of the respective fs- and ns-TA spectra. Statistical noise without any observable residual kinetic trace represents a good quality of fit.

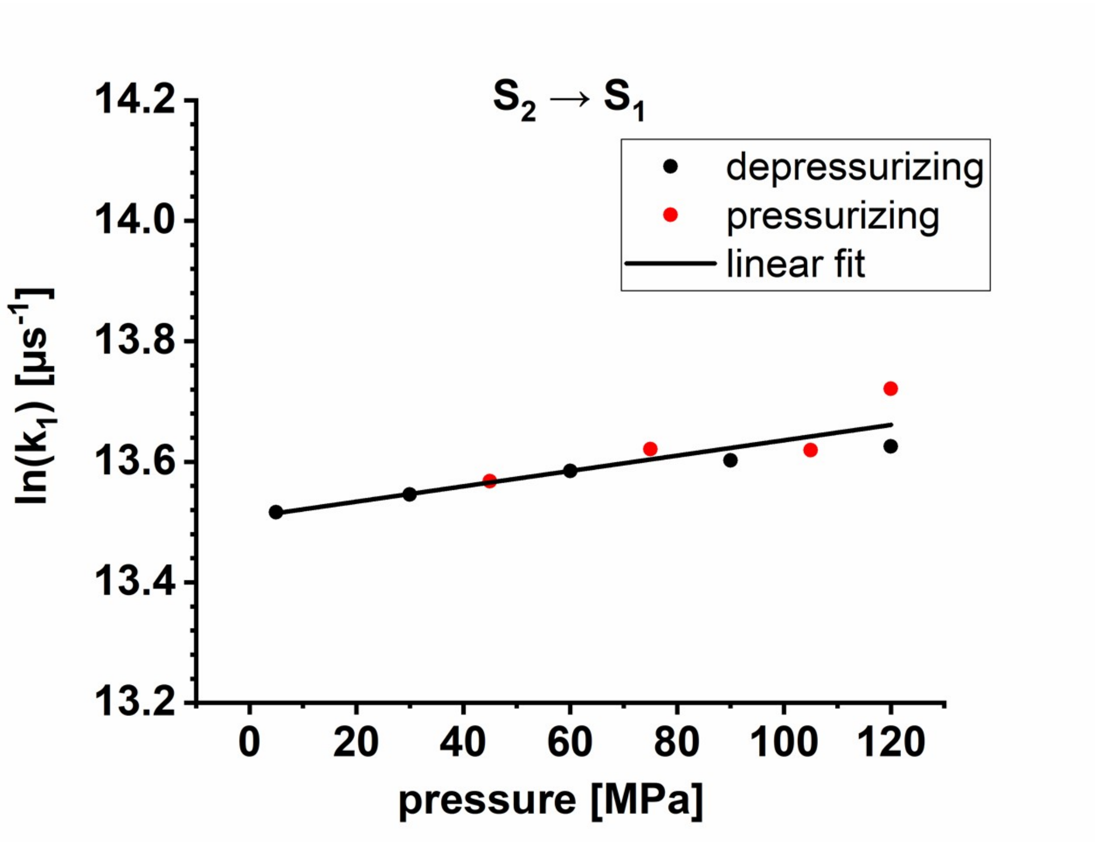

Figure S48. Pressure effect on the reaction rate k_1 of ZnP associated with S_2-S_1 conversion.

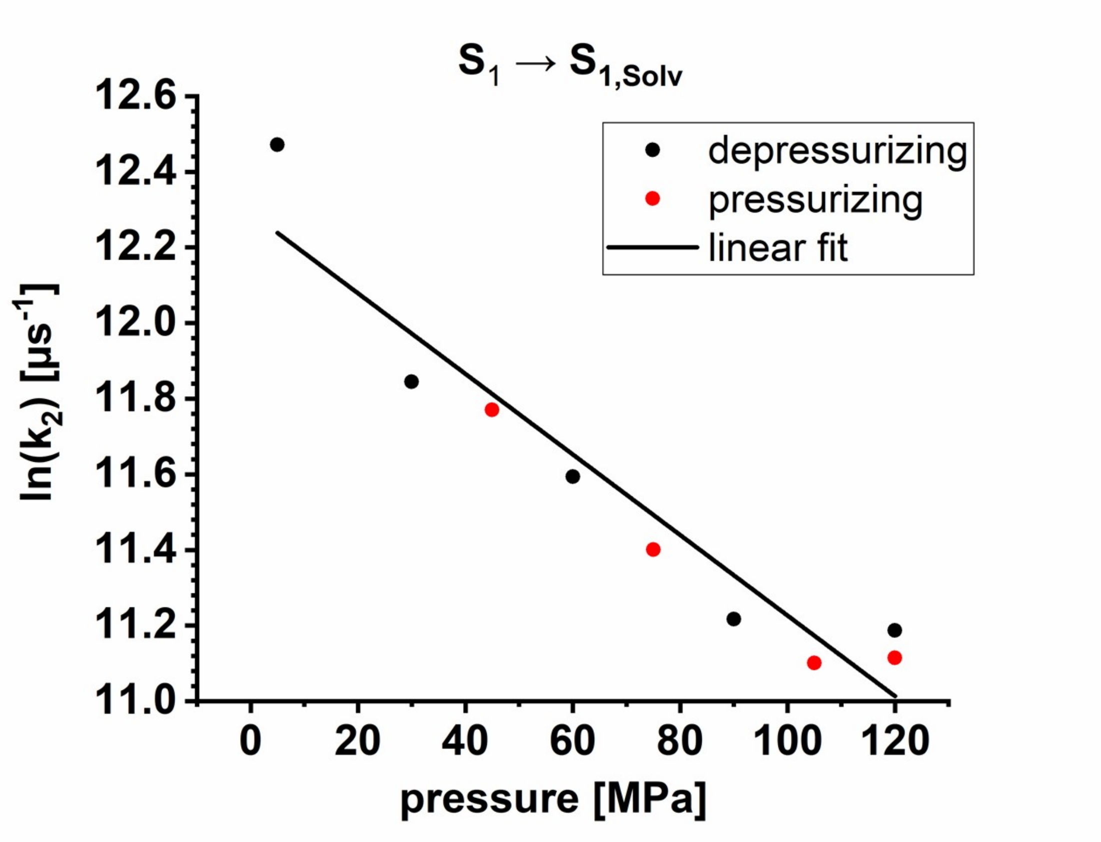

Figure S49. Pressure effect on k_2 of ZnP associated with solvent relaxation from S_1 to the solvated $S_{1,Solv}$.

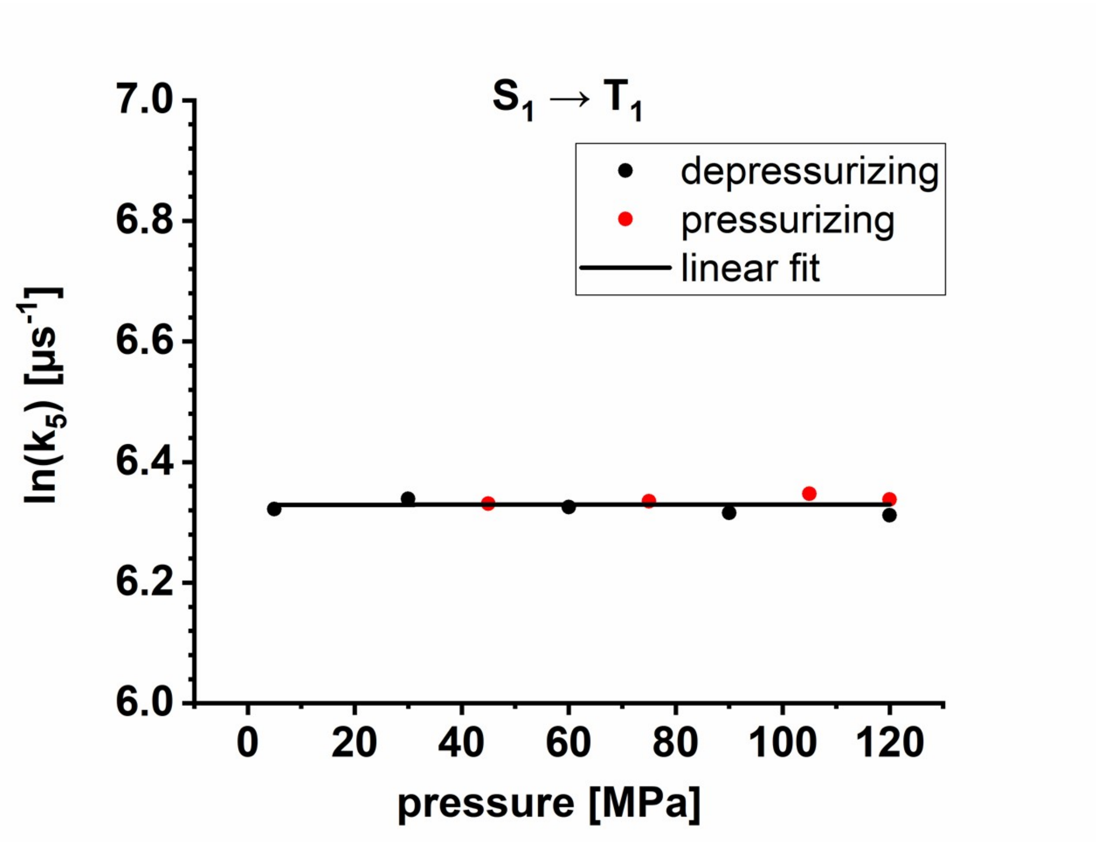

Figure S50. Pressure effect on k_5 of ZnP associated with the intersystem crossing from $S_{1,Solv}$ towards T_1.

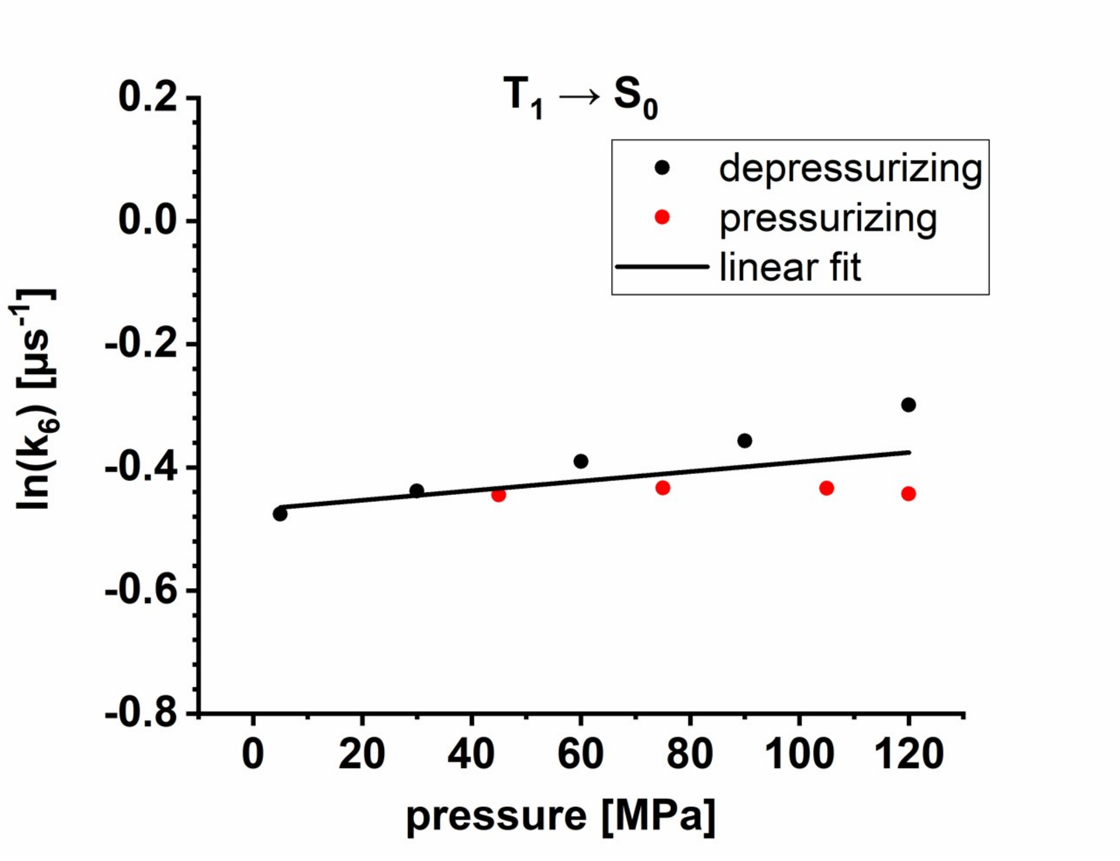

Figure S51. Pressure effect on k_6 of ZnP associated with the ground-state deactivation through oxygen quenching from T_1 to S_0.

9 Literature

(1) J. J. Snellenburg, S. Laptenok, R. Seger, K. M. Mullen and I. H. M. van Stokkum, *J. Stat. Softw.* 2012, **49**, 1 – 22.
(2) M. Spitzer, F. Gartig and R. van Eldik, *Rev. Sci. Instrum*. 1988, **59**, 2092-2093.
(3) I. V. Sazanovich, A. Balakumar, K. Muthukumaran, E. Hindin, C. Kirmaier, J. R. Diers, J. S. Lindsey, D. F. Bocian and D. Holten, D. *Inorg. Chem.* 2003, **42**, 6616-6628.
(4) M. Segura, L. Sánchez, J. de Mendoza, N. Martín and D. M. Guldi, *J. Am. Chem. Soc.* 2003, **125**, 15093-15100.
(5) K.-y. Tomizaki, L. Yu, L. Wei, D. F. Bocian and J. S. Lindsey, *J. Org. Chem.* 2003, **68**, 8199-8207.
(6) J. Otsuki, K. Iwasaki, Y. Nakano, M. Itou, Y. Araki and O. Ito, *Chem. Eur. J.* 2004, **10**, 3461-3466.
(7) T. Yanai, D. P. Tew and N. C. A. Handy, *Chem. Phys. Lett.* 2004, **393**, 51-57.
(8) F. Weigend and R. Ahlrichs, *Phys. Chem. Chem. Phys.* 2005, **7**, 3297-3305.
(9) J. Tomasi, B. Mennucci and R. Cammi, *Chem. Rev.* 2005, **105**, 2999-3094.
(10) S. Boys and F. Bernardi, *Mol. Phys.* 1970, **19**, 553-566.
